# Supplementary material for: Extended-spectrum β-lactamase-producing Enterobacterales among people living with human immunodeficiency virus across the globe: A systematic review and meta-analysis
Source: PLoS One. 2025 Jun 10;20(6):e0321873. doi: 10.1371/journal.pone.0321873 (PMC12151346; doi:10.1371/journal.pone.0321873)
Supplement: SF 2 — (DOCX) [file pone.0321873.s002.docx]

Table, Supplementary file 2, for quality assessment of the studies included in the systematic review and meta-analysis

| Author, Year  (**Cross-sectional**) | Q1 | Q2 | Q3 | Q4 | Q5 | | Q6 | | Q7 | | Q8 | | Q9 | Score out of 9 | Date of extraction | Author who extracted | Remark |
| --- | --- | --- | --- | --- | --- | --- | --- | --- | --- | --- | --- | --- | --- | --- | --- | --- | --- |
| Bayleyegn et al, 2021 [44] | Yes | No | No | Yes | Yes | | Yes | | Yes | | Yes | | UN | 6 | 06 May 2024 | MT | Included |
| Nwokolo et al, 2022 [28] | Yes | No | Yes | No | Yes | | Yes | | Yes | | Yes | | Yes | 7 | 06 May 2024 | MT | Included |
| Endalamaw et al, 2020 [19] | UN | Yes | Yes | Yes | Yes | | Yes | | Yes | | Yes | | Yes | 8 | 06 May 2024 | MT | Included |
| Jerry et al, 2021 [40] | Yes | No | No | Yes | Yes | | Yes | | Yes | | Yes | | No | 6 | 06 May 2024 | MT | Included |
| John-Onwe et al, 2022 [26] | Yes | No | No | Yes | Yes | | Yes | | Yes | | Yes | | Yes | 7 | 07 May 2024 | MT | Included |
| M.R. Rameshkumar et al, 2021 [20] | Yes | Yes | No | No | Yes | | Yes | | Yes | | Yes | | UN | 6 | 07 May 2024 | MT | Included |
| Maharjan et al, 2022 [27] | Yes | No | No | Yes | Yes | | Yes | | Yes | | Yes | | No | 6 | 07 May 2024 | MT | Included |
| Osazuwa et al, 2011 [32] | Yes | Yes | Yes | Yes | Yes | | Yes | | Yes | | UN | | Yes | 8 | 06 May 2024 | KT | Included |
| Zemtsa et al, 2022 [43] | Yes | No | No | Yes | Yes | | Yes | | Yes | | Yes | | No | 6 | 06 May 2024 | KT | Included |
| Dimani et al, 2023 [21] | NA | Yes | No | Yes | Yes | | Yes | | Yes | | Yes | | UN | 6 | 06 May 2024 | KT | Included |
| Falodun et al, 2021 [23] | Yes | No | No | Yes | Yes | | Yes | | Yes | | Yes | | Yes | 7 | 06 May 2024 | KT | Included |
| Manyahi et al, 2020 [24] | Yes | No | Yes | Yes | Yes | | Yes | | Yes | | Yes | | UN | 7 | 07 May 2024 | KT | Included |
| Subramanya et al, 2019 [31] | Yes | No | No | Yes | Yes | | Yes | | Yes | | Yes | | Yes | 7 | 07 May 2024 | KT | Included |
| Surgers et al, 2022 [25] | Yes | No | Yes | Yes | Yes | | Yes | | Yes | | Yes | | UN | 7 | 07 May 2024 | KT | Included |
| Wilmore et al, 2017 [30] | Yes | No | No | Yes | Yes | | Yes | | Yes | | Yes | | Yes | 7 | 08 May 2024 | KT | Included |
| Said et al, 2022 [41] | No | No | No | Yes | Yes | | Yes | | Yes | | Yes | | No | 5 | 08 May 2024 | KT | Included |
| Simeneh et al, 2022 [42] | Yes | Yes | No | Yes | Yes | | Yes | | Yes | | Yes | | UN | 7 | 09 May 2024 | AB | Included |
| Singh et al, 2020 [29] | Yes | No | No | Yes | Yes | | Yes | | Yes | | Yes | | Yes | 7 | 09 May 2024 | AB | Included |
| **Case-control studies** | Q1 | Q2 | Q3 | Q4 | | Q5 | | Q6 | Q7 | Q8 | | Q9 | Q10 |  | - |  |  |
| Reinheimer et al, 2017 [45] | Yes | Yes | Yes | Yes | | Yes | | No | No | Yes | | Yes | Yes | 8 | 10 May 2024 | MT | Included |
| Padmavathy et al, 2011 [46] | Yes | Yes | Yes | Yes | | Yes | | No | No | Yes | | No | Yes | 8 | 10 May 2024 | MT | Included |
| Author, Year  (**Cross-sectional**) | | Q1 | Q2 | Q3 | | Q4 | | Q5 | Q6 | Q7 | | Q8 | Q9 | Score out of 9 | Date of extraction | Author who extracted | Remark |
| Udoh et al, 2018 | | No | No | Yes | | Yes | | Yes | Yes | No | | No | No | 4 | 06 May 2024 | MT | Excluded |
| Padmavathy et al, 2013 | | No | No | No | | Yes | | Yes | Yes | Yes | | No | UN | 4 | 06 May 2024 | MT | Excluded |
| Omo-Omorodion et al, 2021 | | No | No | No | | Yes | | Yes | Yes | Yes | | No | UN | 4 | 08 May 2024 | KT | Excluded |
| Olaru et al, 2021 | | No | No | No | | Yes | | Yes | Yes | Yes | | No | UN | 4 | 08 May 2024 | KT | Excluded |
| Ali et al, 2020 | | No | No | No | | Yes | | Yes | Yes | Yes | | No | UN | 4 | 10 May 2024 | MT | Excluded |
| Adeyemi et al, 2023 | | No | No | No | | Yes | | Yes | Yes | Yes | | No | UN | 4 | 10 May 2024 | MT | Excluded |

Note: UN=unclear, NA= Not avalible, Q= question,

The overall score is calculated by counting the number of Yes’s in each row.

Q1: Was the sample frame appropriate to address the target population?, Q2: Were study participants sampled in an appropriate way?, Q3: Was the sample size adequate?, Q4: Were the study subjects and the setting described in detail? Q5: Was the data analysis conducted with sufficient coverage of the identified sample?, Q6 : Were valid methods used for the identification of the condition?, Q7: Was the condition measured in a standard reliable way for all participants? Q8: Was there appropriate statistical analysis?, and Q9: Was the response rate adequate, and if not, was the low response rate managed appropriately were for cross-sectional studies.

**The following questions were for case-control studies.**

Q1: Were the groups comparable other than the presence of disease in cases or the absence of disease in controls?, Q2: Were cases and controls matched appropriately?, Q3: Were the same criteria used for identification of cases and controls?, Q4: Was exposure measured in a standard, valid and reliable way?, Q5: Was exposure measured in the same way for cases and controls?, Q6, Were confounding factors identified?,Q7: Were strategies to deal with confounding factors stated?, Q8: Were outcomes assessed in a standard, valid and reliable way for cases and controls?, Q9: Was the exposure period of interest long enough to be meaningful?, Q10: Was appropriate statistical analysis used?
