# Supplementary material for: Extended-spectrum β-lactamase-producing Enterobacterales among people living with human immunodeficiency virus across the globe: A systematic review and meta-analysis
Source: PLoS One. 2025 Jun 10;20(6):e0321873. doi: 10.1371/journal.pone.0321873 (PMC12151346; doi:10.1371/journal.pone.0321873)

SF 4. Supplementary file

SF Table. Egger’s test for ESBL-producing *Enterobacterales* among HIV patients


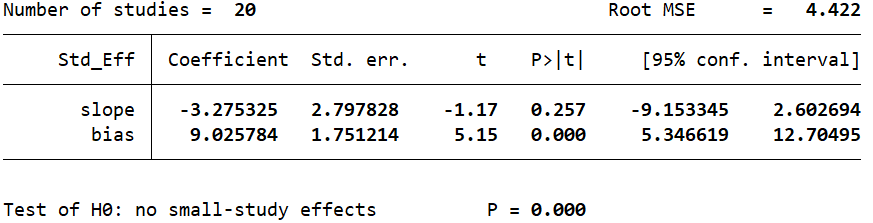


SF Table. Nonparametric trim-and-fill analysis of publication bias imputing on the left


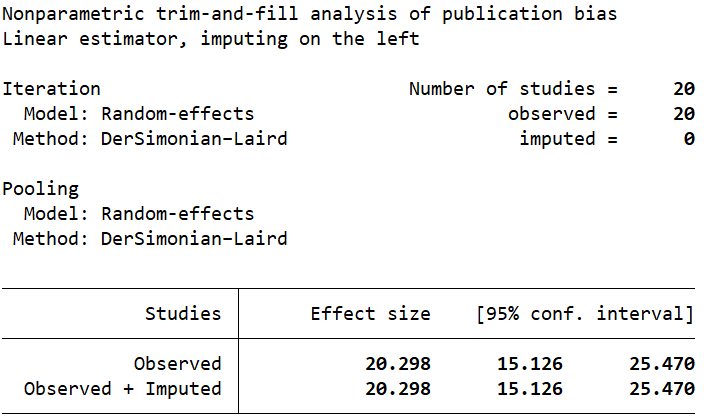


SF Table. Nonparametric trim-and-fill analysis of publication bias imputing on the right


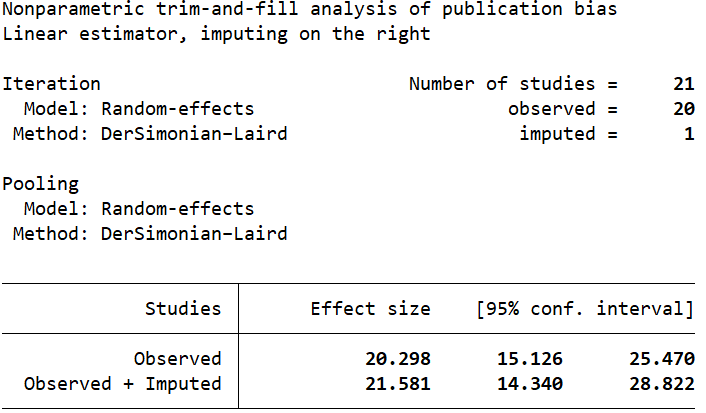


**The following fig and table showed that Sensitivity analysis**

SF, Fig. Sensitivity analysis of ESBL-producing *Enterobacterales* among HIV-positive individuals

SF, Table. Sensitivity analysis of ESBL-producing *Enterobacterales* among HIV-positive individuals


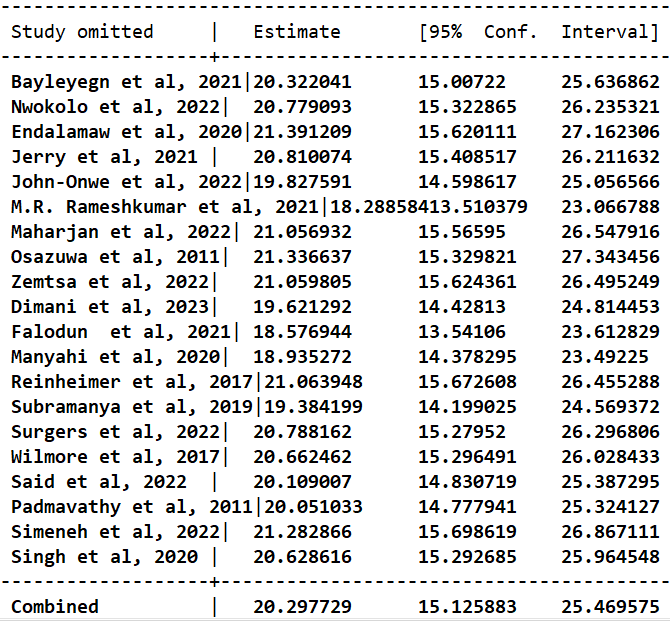

Supplement: SF 3 — (DOCX) [file pone.0321873.s003.docx]
