# Supplementary material for: Extended-spectrum β-lactamase-producing Enterobacterales among people living with human immunodeficiency virus across the globe: A systematic review and meta-analysis
Source: PLoS One. 2025 Jun 10;20(6):e0321873. doi: 10.1371/journal.pone.0321873 (PMC12151346; doi:10.1371/journal.pone.0321873)
Supplement: SF 4 — (DOCX) [file pone.0321873.s004.docx]

**Pooled prevalence of ESBL-producing *Enterobacterales* species among HIV patients**

**Fig. 1 Pooled prevalence of E. coli among HIV patients**

**Fig. 2 Pooled prevalence of** ESBL-producing ***K. pneumonia*e among HIV patients**

**Fig. 3 Pooled prevalence of** ESBL-producing *Proteus* species among HIV patients

**Fig. 4 Pooled prevalence of** ESBL-producing *Salmonella* species among HIV patients

**Fig. 5 Pooled prevalence of** ESBL-producing ***Enterobacter* species among HIV patients**

**Fig. 6 Pooled prevalence of** ESBL-producing ***Serratia* species among HIV patients**

**Fig. 7 Pooled prevalence of ESBL-producing *Citrobacter* species among HIV patients**

**Fig.8** Colonization with ESBL-producing *E. coli* among HIV patients

**Fig. 10** Colonization with ESBL-producing *K. pneumoniae* among HIV patients

**Fig. 11** Infection with ESBL-producing *E. coli* among HIV patients

Fig. 12 Infection with ESBL-producing *K. pneumoniae* among HIV patients
