# Supplementary material for: Extended-spectrum β-lactamase-producing Enterobacterales among people living with human immunodeficiency virus across the globe: A systematic review and meta-analysis
Source: PLoS One. 2025 Jun 10;20(6):e0321873. doi: 10.1371/journal.pone.0321873 (PMC12151346; doi:10.1371/journal.pone.0321873)
Supplement: SF 5 — (DOCX) [file pone.0321873.s005.docx]

Subgroup analysis

Africa

Asia

Europe

By continent

By publication year

By status

By methods

By design

By age

By investigation methods
