# Supplementary material for: Extended-spectrum β-lactamase-producing Enterobacterales among people living with human immunodeficiency virus across the globe: A systematic review and meta-analysis
Source: PLoS One. 2025 Jun 10;20(6):e0321873. doi: 10.1371/journal.pone.0321873 (PMC12151346; doi:10.1371/journal.pone.0321873)
Supplement: SF 6 — (DOCX) [file pone.0321873.s006.docx]

Table for all studies identified in the literature search

| **Excluded articles** | **Reason for exclusion** |
| --- | --- |
| [1-147] | Unrelated topic (n=147) |
| [148-343] | Other than HIV patients (n=196) |
| [344-386] | Mixed/non-human population (n=43) |
| [387-392] | Review and meta-analysis (n=6) |
| [393, 394] | Pilot study (n=1) and Preprint (n=1) |
| [395, 396] | Dissertation and Abstract only (n=2) |
| [397-405] | Wrong period (n=9) |
| [406-444] | None *Enterobacterals* or other pathogens were mixed (n=39) |
| [445-455] | Outcome (ESBL) not reported, either it mixed with other outcome or difficult to separate ESBL by HIV status (n=11) |
| [456-461] | Not fulfilled the quality criteria (6) |
| **Included articles** | **Reason for inclusion** |
| [462-481] | Fulfilled both the inclusion and quality criteria (n=20) |

1. Abali H, Ortakoylu MG. A rare case series of HIV-negative patients with early relapsing cervical tuberculosis lymphadenitis. *Int J Mycobacteriol*. 2022;**11**(4):448-53. 10.4103/ijmy.ijmy_165_22

2. Abdul J, Adegbite BR, Ndanga MED, Edoa JR, Mevyann RC, Mfoumbi G, et al. Resistance patterns among drug-resistant tuberculosis patients and trends-over-time analysis of national surveillance data in Gabon, Central Africa. *Infection*. 2023;**51**(3):697-704. 10.1007/s15010-022-01941-5

3. Aghokeng AF, Ngo-Giang-Huong N, Huynh THK, Dagnra AY, D'Aquin Toni T, Maiga AI, et al. Prevalence of pretreatment HIV resistance to integrase inhibitors in West African and Southeast Asian countries. *J Antimicrob Chemother*. 2024;**79**(5):1164-8. 10.1093/jac/dkae087

4. Altamirano-Beltrán A, Chiara-Chilet C, López JW, Pons MJ, Maquera-Afaray J. Brevundimonas spp. infection in immunocompromised children: Two case reports. *Germs*. 2023;**13**(1):72-9. 10.18683/germs.2023.1369

5. Anang S, Richard J, Bourassa C, Goyette G, Chiu TJ, Chen HC, et al. Characterization of Human Immunodeficiency Virus (HIV-1) Envelope Glycoprotein Variants Selected for Resistance to a CD4-Mimetic Compound. *J Virol*. 2022;**96**(17):e0063622. 10.1128/jvi.00636-22

6. Anang S, Zhang S, Fritschi C, Chiu TJ, Yang D, Smith Iii AB, et al. V3 tip determinants of susceptibility to inhibition by CD4-mimetic compounds in natural clade A human immunodeficiency virus (HIV-1) envelope glycoproteins. *J Virol*. 2023;**97**(11):e0117123. 10.1128/jvi.01171-23

7. Andrés C, González-Sánchez A, Jiménez M, Márquez-Algaba E, Piñana M, Fernández-Naval C, et al. Emergence of Delta and Omicron variants carrying resistance-associated mutations in immunocompromised patients undergoing sotrovimab treatment with long-term viral excretion. *Clin Microbiol Infect*. 2023;**29**(2):240-6. 10.1016/j.cmi.2022.08.021

8. Apolisi I, Cox H, Tyeku N, Daniels J, Mathee S, Cariem R, et al. Tuberculosis Diagnosis and Preventive Monotherapy Among Children and Adolescents Exposed to Rifampicin-Resistant Tuberculosis in the Household. *Open Forum Infect Dis*. 2023;**10**(3):ofad087. 10.1093/ofid/ofad087

9. Aulicino PC, Momin Z, Rozenszajn M, Monzon A, Arazi-Caillaud S, Bologna R, et al. HIV-1 subtype F integrase polymorphisms external to the catalytic core domain contribute to severe loss of replication capacity in context of the integrase inhibitor resistance mutation Q148H. *J Antimicrob Chemother*. 2022;**77**(10):2793-802. 10.1093/jac/dkac238

10. Balduck M, Laumen JGE, Abdellati S, De Baetselier I, de Block T, Manoharan-Basil SS, et al. Tolerance to Ceftriaxone in Neisseria gonorrhoeae: Rapid Induction in WHO P Reference Strain and Detection in Clinical Isolates. *Antibiotics (Basel)*. 2022;**11**(11). 10.3390/antibiotics11111480

11. Bartlett JGJIdcoNA. Pneumonia in the patient with HIV infection. 1998;**12**(3):807-20.

12. Beesham I, Parikh UM, Mellors JW, Joseph Davey DL, Heffron R, Palanee-Phillips T, et al. High Levels of Pretreatment HIV-1 Drug Resistance Mutations Among South African Women Who Acquired HIV During a Prospective Study. *J Acquir Immune Defic Syndr*. 2022;**91**(2):130-7. 10.1097/qai.0000000000003027

13. Black CA, Benavides R, Bandy SM, Dallas SD, Gawrys G, So W, et al. Diverse Role of bla(CTX-M) and Porins in Mediating Ertapenem Resistance among Carbapenem-Resistant Enterobacterales. *Antibiotics (Basel)*. 2024;**13**(2). 10.3390/antibiotics13020185

14. Bwire GM, Aiko BG, Mosha IH, Kilapilo MS, Mangara A, Kazonda P, et al. High viral suppression and detection of dolutegravir-resistance associated mutations in treatment-experienced Tanzanian adults living with HIV-1 in Dar es Salaam. *Sci Rep*. 2023;**13**(1):20493. 10.1038/s41598-023-47795-1

15. Carey ME, Dyson ZA, Ingle DJ, Amir A, Aworh MK, Chattaway MA, et al. Global diversity and antimicrobial resistance of typhoid fever pathogens: Insights from a meta-analysis of 13,000 Salmonella Typhi genomes. *Elife*. 2023;**12**. 10.7554/eLife.85867

16. Cella E, Giovanetti M, Benedetti F, Scarpa F, Johnston C, Borsetti A, et al. Joining Forces against Antibiotic Resistance: The One Health Solution. *Pathogens*. 2023;**12**(9). 10.3390/pathogens12091074

17. Ceña-Diez R, Spetz AL, Sönnerborg A. Synergistic antiviral activity against drug-resistant HIV-1 by naturally occurring dipeptide and A single-stranded oligonucleotide. *Drug Resist Updat*. 2023;**68**:100955. 10.1016/j.drup.2023.100955

18. Charani E, Mendelson M, Pallett SJC, Ahmad R, Mpundu M, Mbamalu O, et al. An analysis of existing national action plans for antimicrobial resistance-gaps and opportunities in strategies optimising antibiotic use in human populations. *Lancet Glob Health*. 2023;**11**(3):e466-e74. 10.1016/s2214-109x(23)00019-0

19. Charles H, Prochazka M, Thorley K, Crewdson A, Greig DR, Jenkins C, et al. Outbreak of sexually transmitted, extensively drug-resistant Shigella sonnei in the UK, 2021-22: a descriptive epidemiological study. *Lancet Infect Dis*. 2022;**22**(10):1503-10. 10.1016/s1473-3099(22)00370-x

20. Chen GJ, Cheng CY, Yang CJ, Lee NY, Tang HJ, Huang SH, et al. Trends of pre-treatment drug resistance in antiretroviral-naïve people with HIV-1 in the era of second-generation integrase strand-transfer inhibitors in Taiwan. *J Antimicrob Chemother*. 2024;**79**(5):1157-63. 10.1093/jac/dkae086

21. Cheng Q, Ma Z, Gong Z, Liang Y, Guo J, Ye X, et al. Whole-genome sequencing analysis of Klebsiella aerogenes among men who have sex with men in Guangzhou, China. *Front Microbiol*. 2023;**14**:1102907. 10.3389/fmicb.2023.1102907

22. Chohan BH, Kingston H, Tseng AS, Sambai B, Guthrie BL, Wilkinson E, et al. Virologic Nonsuppression and HIV Drug Resistance Among People Who Inject Drugs and Their Sexual and Injecting Partners in Kenya. *AIDS Res Hum Retroviruses*. 2024;**40**(5):240-50. 10.1089/aid.2023.0068

23. Cilento ME, Wen X, Reeve AB, Ukah OB, Snyder AA, Carrillo CM, et al. HIV-1 Resistance to Islatravir/Tenofovir Combination Therapy in Wild-Type or NRTI-Resistant Strains of Diverse HIV-1 Subtypes. *Viruses*. 2023;**15**(10). 10.3390/v15101990

24. De Francesco MA, Gargiulo F, Zaltron S, Spinetti A, Castelli F, Caruso A. DAA Treatment Failure in a HIV/HBV/HCV Co-Infected Patient Carrying a Chimeric HCV Genotype 4/1b. *Int J Environ Res Public Health*. 2022;**19**(18). 10.3390/ijerph191811655

25. De Koster S, Ringenier M, Xavier BB, Lammens C, De Coninck D, De Bruyne K, et al. Genetic characterization of ESBL-producing and ciprofloxacin-resistant Escherichia coli from Belgian broilers and pigs. *Front Microbiol*. 2023;**14**:1150470. 10.3389/fmicb.2023.1150470

26. De Koster S, Xavier BB, Lammens C, Perales Selva N, van Kleef-van Koeveringe S, Coenen S, et al. One Health surveillance of colistin-resistant Enterobacterales in Belgium and the Netherlands between 2017 and 2019. *PLoS One*. 2024;**19**(2):e0298096. 10.1371/journal.pone.0298096

27. de Salazar A, Viñuela L, Fuentes A, Teyssou E, Charpentier C, Lambert-Niclot S, et al. Transmitted Drug Resistance to Integrase-Based First-Line Human Immunodeficiency Virus Antiretroviral Regimens in Mediterranean Europe. *Clin Infect Dis*. 2023;**76**(9):1628-35. 10.1093/cid/ciac972

28. Dekhil N, Mardassi H. On the onset and dispersal of a major MDR TB clone among HIV-negative patients, Tunisia. *Antimicrob Resist Infect Control*. 2024;**13**(1):18. 10.1186/s13756-023-01360-7

29. Dhabaan G, Jamal H, Ouellette D, Alexander S, Arane K, Campigotto A, et al. Detection of OXA-181 Carbapenemase in Shigella flexneri. *Emerg Infect Dis*. 2024;**30**(5):1048-50. 10.3201/eid3005.231558

30. Dong S, Lu X, Wang Y, An N, Liu M, Li Y, et al. Near-Full-Length Genome Analysis of a HIV-1 CRF01_AE/B Recombinant Strain Among Men Who Have Sex with Men in Chengde City, China. *AIDS Res Hum Retroviruses*. 2024;**40**(5):257-62. 10.1089/aid.2023.0110

31. Doolan JA, Williams GT, Hilton KLF, Chaudhari R, Fossey JS, Goult BT, et al. Advancements in antimicrobial nanoscale materials and self-assembling systems. *Chem Soc Rev*. 2022;**51**(20):8696-755. 10.1039/d1cs00915j

32. Epalza C, Valadés-Alcaraz A, González-Alba JM, Beltrán-Pavez C, Gutiérrez-López M, Rubio-Garrido M, et al. Transmitted Drug Resistance and HIV Diversity Among Adolescents Newly Diagnosed With HIV in Spain. *Pediatr Infect Dis J*. 2024;**43**(1):40-8. 10.1097/inf.0000000000004138

33. Erfaninejad M, Zarei Mahmoudabadi A, Maraghi E, Hashemzadeh M, Fatahinia M. Low level of antifungal resistance in Candida species recovered from Iranian HIV-associated oral infection. *Lett Appl Microbiol*. 2023;**76**(3). 10.1093/lambio/ovad029

34. Faraci G, Park SY, Dubé MP, Lee HY. Full-spectrum HIV drug resistance mutation detection by high-resolution complete pol gene sequencing. *J Clin Virol*. 2023;**164**:105491. 10.1016/j.jcv.2023.105491

35. Freitas VAQ, Santos AS, Zara A, Costa CR, Godoy CSM, Soares RBA, et al. Distribution and antifungal susceptibility profiles of Candida species isolated from people living with HIV/AIDS in a public hospital in Goiânia, GO, Brazil. *Braz J Microbiol*. 2023;**54**(1):125-33. 10.1007/s42770-022-00851-w

36. Gill J, Busca A, Cinatti N, Passera R, Dellacasa CM, Giaccone L, et al. Bacterial Bloodstream Infections after Allogeneic Hematopoietic Stem Cell Transplantation: Etiology, Risk Factors and Outcome in a Single-Center Study. *Microorganisms*. 2023;**11**(3). 10.3390/microorganisms11030742

37. Goletti D, Pisapia R, Fusco FM, Aiello A, Van Crevel R. Epidemiology, pathogenesis, clinical presentation and management of TB in patients with HIV and diabetes. *Int J Tuberc Lung Dis*. 2023;**27**(4):284-90. 10.5588/ijtld.22.0685

38. Golparian D, Bazzo ML, Ahlstrand J, Schörner MA, Gaspar PC, de Melo Machado H, et al. Recent dynamics in Neisseria gonorrhoeae genomic epidemiology in Brazil: antimicrobial resistance and genomic lineages in 2017-20 compared to 2015-16. *J Antimicrob Chemother*. 2024;**79**(5):1081-92. 10.1093/jac/dkae075

39. González N, Abdellati S, De Baetselier I, Laumen JGE, Van Dijck C, Block T, et al. Ciprofloxacin Concentrations 1/1000th the MIC Can Select for Antimicrobial Resistance in N. gonorrhoeae-Important Implications for Maximum Residue Limits in Food. *Antibiotics (Basel)*. 2022;**11**(10). 10.3390/antibiotics11101430

40. Hadavandsiri F, Shafaati M, Mohammad Nejad S, Ebrahimzadeh Mousavi M, Najafi A, Mirzaei M, et al. Non-communicable disease comorbidities in HIV patients: diabetes, hypertension, heart disease, and obstructive sleep apnea as a neglected issue. *Sci Rep*. 2023;**13**(1):12730. 10.1038/s41598-023-39828-6

41. Hamers RL, de Wit TFR, Holmes CBJTLH. HIV drug resistance in low-income and middle-income countries. 2018;**5**(10):e588-e96.

42. Hayakawa K, Matsumura Y, Uemura K, Tsuzuki S, Sakurai A, Tanizaki R, et al. Effectiveness of cefmetazole versus meropenem for invasive urinary tract infections caused by extended-spectrum β-lactamase-producing Escherichia coli. *Antimicrob Agents Chemother*. 2023;**67**(10):e0051023. 10.1128/aac.00510-23

43. Hermans LE, Centner CM, Morel CM, Mbamalu O, Bonaconsa C, Ferreyra C, et al. Point-of-care diagnostics for infection and antimicrobial resistance in sub-Saharan Africa: A narrative review. *Int J Infect Dis*. 2024;**142**:106907. 10.1016/j.ijid.2023.11.027

44. Hermans LE, Umunnakwe CN, Lalla-Edward ST, Hebel SK, Tempelman HA, Nijhuis M, et al. Point-of-Care Tenofovir Urine Testing for the Prediction of Treatment Failure and Drug Resistance During Initial Treatment for Human Immunodeficiency Virus Type 1 (HIV-1) Infection. *Clin Infect Dis*. 2023;**76**(3):e553-e60. 10.1093/cid/ciac755

45. Hinay AA, Jr., Kanai K, Tsuneki-Tokunaga A, Komatsu M, Telan EO, Kageyama S. In Vitro Susceptibility of HIV Isolates with High Growth Capability to Antiretroviral Drugs. *Int J Mol Sci*. 2022;**23**(23). 10.3390/ijms232315380

46. Houri H, Aghdaei HA, Firuzabadi S, Khorsand B, Soltanpoor F, Rafieepoor M, et al. High Prevalence Rate of Microbial Contamination in Patient-Ready Gastrointestinal Endoscopes in Tehran, Iran: an Alarming Sign for the Occurrence of Severe Outbreaks. *Microbiol Spectr*. 2022;**10**(5):e0189722. 10.1128/spectrum.01897-22

47. Isac R, Costa R, Frandes M, Lazureanu VE, Stroescu RF, Steflea RM, et al. Renal Impairment Impact and Survival Analysis in a Romanian Cohort of HIV-1(F1)-Infected Children and Adolescents. *Life (Basel)*. 2023;**13**(4). 10.3390/life13040888

48. Jünger C, Imkamp F, Balakrishna S, Gysin M, Haldimann K, Brugger SD, et al. Phenotypic and genotypic characterization of Neisseria gonorrhoeae isolates among individuals at high risk for sexually transmitted diseases in Zurich, Switzerland. *Int J STD AIDS*. 2024;**35**(6):462-70. 10.1177/09564624241230266

49. Ka'e AC, Fokam J, Togna Pabo WLR, Nanfack A, Ngoufack Jagni Semengue E, Bouba Y, et al. Evaluation of archived drug resistance mutations in HIV-1 DNA among vertically infected adolescents under antiretroviral treatment in Cameroon: Findings during the COVID-19 pandemic. *HIV Med*. 2023;**24**(6):691-702. 10.1111/hiv.13459

50. Kakooza F, Golparian D, Matoga M, Maseko V, Lamorde M, Krysiak R, et al. Genomic surveillance and antimicrobial resistance determinants in Neisseria gonorrhoeae isolates from Uganda, Malawi and South Africa, 2015-20. *J Antimicrob Chemother*. 2023;**78**(8):1982-91. 10.1093/jac/dkad193

51. Kaur H, Singh I, Modgil V, Singh N, Mohan B, Taneja N. Genome sequence of pan drug-resistant enteroaggregative Escherichia coli belonging to ST38 clone from India, an emerging EAEC/UPEC hybrid pathotype. *Indian J Med Microbiol*. 2024;**49**:100606. 10.1016/j.ijmmb.2024.100606

52. Kc R, Adhikari S, Bastola A, Devkota L, Bhandari P, Ghimire P, et al. Opportunistic Respiratory Infections in HIV Patients Attending Sukraraj Tropical and Infectious Diseases Hospital in Kathmandu, Nepal. *HIV AIDS (Auckl)*. 2019;**11**:357-67. 10.2147/hiv.S229531

53. Kemp SA, Kamelian K, Cuadros DF, Cheng MTK, Okango E, Hanekom W, et al. HIV transmission dynamics and population-wide drug resistance in rural South Africa. *Nat Commun*. 2024;**15**(1):3644. 10.1038/s41467-024-47254-z

54. Kenyon C. Doxycycline post-exposure prophylaxis could theoretically select for resistance to various antimicrobials in 19 pathobionts: an in silico analysis. *Int J Infect Dis*. 2024;**142**:106974. 10.1016/j.ijid.2024.02.017

55. Kenyon C, Gestels Z, Vanbaelen T, Abdellati S, Van Den Bossche D, De Baetselier I, et al. Doxycycline PEP can induce doxycycline resistance in Klebsiella pneumoniae in a Galleria mellonella model of PEP. *Front Microbiol*. 2023;**14**:1208014. 10.3389/fmicb.2023.1208014

56. Kim S, Hesseling AC, Wu X, Hughes MD, Shah NS, Gaikwad S, et al. Factors associated with prevalent Mycobacterium tuberculosis infection and disease among adolescents and adults exposed to rifampin-resistant tuberculosis in the household. *PLoS One*. 2023;**18**(3):e0283290. 10.1371/journal.pone.0283290

57. Kirichenko A, Kireev D, Lapovok I, Shlykova A, Lopatukhin A, Pokrovskaya A, et al. HIV-1 Drug Resistance among Treatment-Naïve Patients in Russia: Analysis of the National Database, 2006-2022. *Viruses*. 2023;**15**(4). 10.3390/v15040991

58. Kiros M, Biset S, Gebremariam B, Yalew GT, Abegaz WE, Geteneh A. Trends in HIV-1 pretreatment drug resistance and HIV-1 variant dynamics among antiretroviral therapy-naive Ethiopians from 2003 to 2018: a pooled sequence analysis. *Virol J*. 2023;**20**(1):243. 10.1186/s12985-023-02205-w

59. Kiros M, Tefera DA, Andualem H, Geteneh A, Tesfaye A, Woldemichael TS, et al. Low level of HIV-1C integrase strand transfer inhibitor resistance mutations among recently diagnosed ART-naive Ethiopians. *Sci Rep*. 2023;**13**(1):6546. 10.1038/s41598-023-33850-4

60. Kountchou CL, Noubom M, Ndezo Bisso B, Ngouana Kammalac T, Ekpo AI, Ngueguim Dougue A, et al. Antifungal Resistance Profile, Biofilm Formation, and Virulence Factor Production in Candida krusei Isolates From HIV-Infected Patients in Cameroon. *Cureus*. 2023;**15**(8):e44213. 10.7759/cureus.44213

61. Kuo JY, Yeh CS, Wang SM, Chen SH, Wang JR, Chen TY, et al. Acyclovir-resistant HSV-1 isolates among immunocompromised patients in southern Taiwan: Low prevalence and novel mutations. *J Med Virol*. 2023;**95**(8):e28985. 10.1002/jmv.28985

62. Le X, Qian X, Liu L, Sun J, Song W, Qi T, et al. Trends in and Risk Factors for Drug Resistance in Mycobacterium tuberculosis in HIV-Infected Patients. *Viruses*. 2024;**16**(4). 10.3390/v16040627

63. Li S, Wang Z, Song S, Tang Y, Zhou J, Liu X, et al. Membrane-Active All-Hydrocarbon-Stapled α-Helical Amphiphilic Tat Peptides: Broad-Spectrum Antibacterial Activity and Low Incidence of Drug Resistance. *ACS Infect Dis*. 2024;**10**(5):1839-55. 10.1021/acsinfecdis.4c00173

64. Li Z, Li Y, Li S, Li Z, Mai Y, Cheng J, et al. Identification of a novel drug-resistant community-acquired Nocardia spp. in a patient with bronchiectasis. *Emerg Microbes Infect*. 2022;**11**(1):1346-55. 10.1080/22221751.2022.2069514

65. Liew KC, O'Keeffe J, Rajandas H, Lee YP, Harris O, Parimannan S, et al. Insights into the Evolution of P. aeruginosa Antimicrobial Resistance in a Patient Undergoing Intensive Therapy. *Antibiotics (Basel)*. 2023;**12**(3). 10.3390/antibiotics12030483

66. Lin TH, Wu CC, Tseng CY, Fang JH, Lin CT. Effects of gallic acid on capsular polysaccharide biosynthesis in Klebsiella pneumoniae. *J Microbiol Immunol Infect*. 2022;**55**(6 Pt 2):1255-62. 10.1016/j.jmii.2021.07.002

67. Liu J, Li C, Sun Y, Fu C, Wei S, Zhang X, et al. Characteristics of drug resistance mutations in ART-experienced HIV-1 patients with low-level viremia in Zhengzhou City, China. *Sci Rep*. 2024;**14**(1):10620. 10.1038/s41598-024-60965-z

68. Liu Y, Huang L, Cai J, Zhu H, Li J, Yu Y, et al. Clinical characteristics of respiratory tract infection caused by Klebsiella pneumoniae in immunocompromised patients: a retrospective cohort study. *Front Cell Infect Microbiol*. 2023;**13**:1137664. 10.3389/fcimb.2023.1137664

69. Lu Y, Pang W, Zhang MD, Song JH, Shen F, He WQ, et al. A Novel Vpu Adaptive Mutation of HIV-1 Degrades Tetherin in Northern Pig-Tailed Macaques (Macaca leonina) Mainly via the Ubiquitin-Proteasome Pathway and Increases Viral Release. *J Virol*. 2023;**97**(4):e0020023. 10.1128/jvi.00200-23

70. Lynch RM, Bar KJ. Development of screening assays for use of broadly neutralizing antibodies in people with HIV. *Curr Opin HIV AIDS*. 2023;**18**(4):171-7. 10.1097/coh.0000000000000798

71. Madaan K, Bari VK. Emerging Role of Sphingolipids in Amphotericin B Drug Resistance. *Microb Drug Resist*. 2023;**29**(8):319-32. 10.1089/mdr.2022.0353

72. Magrini E, Rando E, Del Giacomo P, Matteini E, Leanza GM, Sanmartin F, et al. Cerebrospinal fluid drain infection caused by pandrug-resistant Staphylococcus epidermidis successfully treated with ceftaroline in combination with fosfomycin and vancomycin. *Diagn Microbiol Infect Dis*. 2024;**109**(1):116205. 10.1016/j.diagmicrobio.2024.116205

73. Mangala C, Maulot-Bangola D, Moutsinga A, Wamba AG, Okolongo-Mayani SC, Fokam J. Current Resistance of HIV-1 Strains Isolated in Volunteer Blood Donors in Gabon. *AIDS Res Hum Retroviruses*. 2024;**40**(5):341-6. 10.1089/aid.2023.0080

74. Mansouri M, Rumrill S, Dawson S, Johnson A, Pinson JA, Gunzburg MJ, et al. Targeting HIV-1 Reverse Transcriptase Using a Fragment-Based Approach. *Molecules*. 2023;**28**(7). 10.3390/molecules28073103

75. Manyahi J, Moyo SJ, Langeland N, Blomberg B. Genetic determinants of macrolide and tetracycline resistance in penicillin non-susceptible Streptococcus pneumoniae isolates from people living with HIV in Dar es Salaam, Tanzania. *Ann Clin Microbiol Antimicrob*. 2023;**22**(1):16. 10.1186/s12941-023-00565-3

76. Martin K, Dziva Chikwari C, Dauya E, Mackworth-Young CRS, Bath D, Tucker J, et al. Investigating point-of-care diagnostics for sexually transmitted infections and antimicrobial resistance in antenatal care in Zimbabwe (IPSAZ): protocol for a mixed-methods study. *BMJ Open*. 2023;**13**(4):e070889. 10.1136/bmjopen-2022-070889

77. Martin-Blondel G, Marcelin AG, Soulié C, Kaisaridi S, Lusivika-Nzinga C, Zafilaza K, et al. Time to negative PCR conversion amongst high-risk patients with mild-to-moderate Omicron BA.1 and BA.2 COVID-19 treated with sotrovimab or nirmatrelvir. *Clin Microbiol Infect*. 2023;**29**(4):543.e5-.e9. 10.1016/j.cmi.2022.12.016

78. Martinson T, Nwogu-Attah J, Spinelli M, Gandhi M. Low-cost urine tenofovir assay to triage dolutegravir resistance testing. *Lancet HIV*. 2024;**11**(5):e282-e3. 10.1016/s2352-3018(24)00060-2

79. Mathur S, Smuk M, Evans C, Wedderburn CJ, Gibb DM, Penazzato M, et al. Estimating the impact of alternative programmatic cotrimoxazole strategies on mortality among children born to mothers with HIV: A modelling study. *PLoS Med*. 2024;**21**(2):e1004334. 10.1371/journal.pmed.1004334

80. Matran YM, Al-Haddad AM, Kour A, Al-Shehabi H, Sharma S, Suttee A, et al. Streptococcus pneumoniae among the children of Aden, Yemen: a cross-sectional report of post-pneumococcal conjugate vaccine. *J Infect Dev Ctries*. 2024;**18**(4):579-86. 10.3855/jidc.18935

81. Maurer FP, Shubladze N, Kalmambetova G, Felker I, Kuchukhidze G, Köser CU, et al. Diagnostic Capacities for Multidrug-Resistant Tuberculosis in the World Health Organization European Region: Action is Needed by all Member States. *J Mol Diagn*. 2022;**24**(11):1189-94. 10.1016/j.jmoldx.2022.07.005

82. Mbamalu O, Surendran S, Nampoothiri V, Bonaconsa C, Edathadathil F, Zhu N, et al. Survey of healthcare worker perceptions of changes in infection control and antimicrobial stewardship practices in India and South Africa during the COVID-19 pandemic. *IJID Reg*. 2023;**6**:90-8. 10.1016/j.ijregi.2022.11.010

83. Morgan JA, Hankins ME, Callais NA, Albritton CW, Vanchiere JA, Betcher RE, et al. Group B Streptococcus Rectovaginal Colonization and Resistance Patterns in HIV-Positive Compared to HIV-Negative Pregnant Patients. *Am J Perinatol*. 2023;**40**(14):1573-8. 10.1055/s-0041-1739356

84. Mulinge MM, Oluoch JO, Abisi HK, Otieno LE, Anzala O, Wamalwa DC, et al. Age and CD4+ T cell counts are inversely associated with HIV drug resistance mutations in treatment naive female sex workers. *Medicine (Baltimore)*. 2023;**102**(24):e34060. 10.1097/md.0000000000034060

85. Nastri BM, Pagliano P, Zannella C, Folliero V, Masullo A, Rinaldi L, et al. HIV and Drug-Resistant Subtypes. *Microorganisms*. 2023;**11**(1). 10.3390/microorganisms11010221

86. Ndashimye E, Reyes PS, Arts EJ. New antiretroviral inhibitors and HIV-1 drug resistance: more focus on 90% HIV-1 isolates? *FEMS Microbiol Rev*. 2023;**47**(1). 10.1093/femsre/fuac040

87. Ngobese B, Swe Swe-Han K, Tinarwo P, Abbai NS. Low prevalence of macrolide resistance in Mycoplasma genitalium infections in a cohort of pregnant women living with human immunodeficiency virus. *Int J STD AIDS*. 2022;**33**(14):1174-82. 10.1177/09564624221129412

88. Nielsen KE, St Cyr SB, Pham CD, Kreisel KM. Assessing the National Representativeness of Estimates of Antimicrobial-Resistant Urogenital Neisseria gonorrhoeae in US Men, Gonococcal Isolate Surveillance Project, 2008-2018. *Sex Transm Dis*. 2023;**50**(4):196-202. 10.1097/olq.0000000000001755

89. Ninyio N, Schmitt K, Sergon G, Nilsson C, Andersson S, Scherbak N. Stable expression of HIV-1 MPER extended epitope on the surface of the recombinant probiotic bacteria Escherichia Coli Nissle 1917 using CRISPR/Cas9. *Microb Cell Fact*. 2024;**23**(1):39. 10.1186/s12934-023-02290-0

90. Noskin GA, Glassroth JJCicm. Bacterial pneumonia associated with HIV-1 infection. 1996;**17**(4):713-23.

91. Nurjannah, Jayanti S, Tanoerahardjo FS, Al Musyahadah US, Sukowati CHC, Massi MN. Major Drug Resistance Mutations on Reverse Transcriptase Gene in Human Immunodeficiency Virus Type-1 in Indonesia: A Systematic Review. *Curr HIV/AIDS Rep*. 2024;**21**(2):31-9. 10.1007/s11904-023-00687-5

92. Nyamankolly E, Bellecave P, Wittkop L, Le Marec F, Duffau P, Lazaro E, et al. Long-term follow-up of HIV-1 multi-drug-resistant treatment-experienced participants treated with etravirine, raltegravir and boosted darunavir: towards drug-reduced regimen? ANRS CO3 Aquitaine Cohort 2007-2018. *Int J Antimicrob Agents*. 2023;**61**(1):106696. 10.1016/j.ijantimicag.2022.106696

93. Nzimande B, Kumalo HM, Ndlovu SI, Mkhwanazi NP. Secondary metabolites produced by endophytic fungi, Alternaria alternata, as potential inhibitors of the human immunodeficiency virus. *Front Genet*. 2022;**13**:1077159. 10.3389/fgene.2022.1077159

94. Obanda BA, Gibbons CL, Fèvre EM, Bebora L, Gitao G, Ogara W, et al. Multi-Drug Resistant Staphylococcus aureus Carriage in Abattoir Workers in Busia, Kenya. *Antibiotics (Basel)*. 2022;**11**(12). 10.3390/antibiotics11121726

95. Ogwang MO, Diero L, Ng'ong'a F, Magoma G, Mutharia L, Imbuga M, et al. Strain structure analysis of Mycobacterium tuberculosis circulating among HIV negative, positive and drug resistant TB patients attending chest clinics in Western Kenya. *BMC Pulm Med*. 2023;**23**(1):497. 10.1186/s12890-023-02802-z

96. Omondi EO, Muigai A, Ngayo MO, Mungiria J, Lihana R. Nevirapine plasma concentration is associated with virologic failure and the emergence of drug-resistant mutations among HIV patients in Kenya: A cross sectional study. *Medicine (Baltimore)*. 2022;**101**(50):e32346. 10.1097/md.0000000000032346

97. Omran BA, Tseng BS, Baek KH. Nanocomposites against Pseudomonas aeruginosa biofilms: Recent advances, challenges, and future prospects. *Microbiol Res*. 2024;**282**:127656. 10.1016/j.micres.2024.127656

98. Ouyang F, Yuan D, Zhai W, Liu S, Zhou Y, Yang H. HIV-1 Drug Resistance Detected by Next-Generation Sequencing among ART-Naïve Individuals: A Systematic Review and Meta-Analysis. *Viruses*. 2024;**16**(2). 10.3390/v16020239

99. Paccoud O, Shuping L, Mashau R, Greene G, Quan V, Meiring S, et al. Impact of prior cryptococcal antigen screening on in-hospital mortality in cryptococcal meningitis or fungaemia among HIV-seropositive individuals in South Africa: a cross-sectional observational study. *Clin Microbiol Infect*. 2023;**29**(8):1063-9. 10.1016/j.cmi.2023.04.016

100. Pahuja I, Verma A, Ghoshal A, Mukhopadhyay S, Kumari A, Shaji A, et al. Biapenem, a Carbapenem Antibiotic, Elicits Mycobacteria Specific Immune Responses and Reduces the Recurrence of Tuberculosis. *Microbiol Spectr*. 2023;**11**(4):e0085823. 10.1128/spectrum.00858-23

101. Pang X, He Q, Tang K, Huang J, Fang N, Xie H, et al. Drug resistance and influencing factors in HIV-1-infected individuals under antiretroviral therapy in Guangxi, China. *J Antimicrob Chemother*. 2024;**79**(5):1142-52. 10.1093/jac/dkae084

102. Panza F, Saladini F, Bartolini N, Gianmarino F, Montagnani F, Spagnuolo V, et al. PRESTIGIO RING "a 59-year-old man with multidrug resistant HIV-1 infection failing a regimen including dolutegravir, rilpivirine, atazanavir/cobicistat: successful treatment tailoring based on genotypic and phenotypic resistance tests". *New Microbiol*. 2024;**47**(1):116-22.

103. Park SY, Faraci G, Ganesh K, Dubé MP, Lee HY. Portable Nanopore sequencing solution for next-generation HIV drug resistance testing. *J Clin Virol*. 2024;**171**:105639. 10.1016/j.jcv.2024.105639

104. Perovic O, Singh A. Nosocomial Infections in patients with human immunodeficiency virus (HIV). HIV Infection in the Era of Highly Active Antiretroviral Treatment and Some of Its Associated Complications: IntechOpen; 2011.

105. Petkov S, Kilpeläinen A, Bayurova E, Latanova A, Mezale D, Fridrihsone I, et al. HIV-1 Protease as DNA Immunogen against Drug Resistance in HIV-1 Infection: DNA Immunization with Drug Resistant HIV-1 Protease Protects Mice from Challenge with Protease-Expressing Cells. *Cancers (Basel)*. 2022;**15**(1). 10.3390/cancers15010238

106. Phinius BB, Anderson M, Gobe I, Mokomane M, Choga WT, Phakedi B, et al. High Prevalence of Hepatitis B Virus Drug Resistance Mutations to Lamivudine among People with HIV/HBV Coinfection in Rural and Peri-Urban Communities in Botswana. *Viruses*. 2024;**16**(4). 10.3390/v16040592

107. Pietersen E, Anderson K, Cox H, Dheda K, Bian A, Shepherd BE, et al. Variation in missed doses and reasons for discontinuation of anti-tuberculosis drugs during hospital treatment for drug-resistant tuberculosis in South Africa. *PLoS One*. 2023;**18**(2):e0281097. 10.1371/journal.pone.0281097

108. Pimentel V, Pingarilho M, Sebastião CS, Miranda M, Gonçalves F, Cabanas J, et al. Applying Next-Generation Sequencing to Track HIV-1 Drug Resistance Mutations Circulating in Portugal. *Viruses*. 2024;**16**(4). 10.3390/v16040622

109. Pinto L, Shastry RP, Alva S, Rao RSP, Ghate SD. Functional network analysis identifies multiple virulence and antibiotic resistance targets in Stenotrophomonas maltophilia. *Microb Pathog*. 2023;**183**:106314. 10.1016/j.micpath.2023.106314

110. Reimche JL, Clemons AA, Chivukula VL, Joseph SJ, Schmerer MW, Pham CD, et al. Genomic analysis of 1710 surveillance-based Neisseria gonorrhoeae isolates from the USA in 2019 identifies predominant strain types and chromosomal antimicrobial-resistance determinants. *Microb Genom*. 2023;**9**(5). 10.1099/mgen.0.001006

111. Reyes-Gualito A, Macías AE, Reyes-Escogido L, Mendoza-Macías CL, Álvarez-Canales JA. Association between phenotypic characteristics of Escherichia coli and UTI recurrence in immunocompromised patients: A case-control study. *Am J Infect Control*. 2024;**52**(1):61-5. 10.1016/j.ajic.2023.08.016

112. Rodríguez-Galet A, Ventosa-Cubillo J, Bendomo V, Eyene M, Mikue-Owono T, Nzang J, et al. High Drug Resistance Levels Compromise the Control of HIV Infection in Pediatric and Adult Populations in Bata, Equatorial Guinea. *Viruses*. 2022;**15**(1). 10.3390/v15010027

113. Rowlinson E, Soge OO, Hughes JP, Berzkalns A, Thibault C, Kerani RP, et al. Prior Exposure to Azithromycin and Azithromycin Resistance Among Persons Diagnosed With Neisseria gonorrhoeae Infection at a Sexual Health Clinic: 2012-2019. *Clin Infect Dis*. 2023;**76**(3):e1270-e6. 10.1093/cid/ciac682

114. Rugemalila J, Kamori D, Kunambi P, Mizinduko M, Sabasaba A, Masoud S, et al. HIV virologic response, patterns of drug resistance mutations and correlates among adolescents and young adults: A cross-sectional study in Tanzania. *PLoS One*. 2023;**18**(2):e0281528. 10.1371/journal.pone.0281528

115. Saikaew S, Thongprachum A, Pongsararuk R, Thanraka A, Kunyanone N, Chaiyasirinroje B, et al. Genotypic Distribution and the Epidemiology of Multidrug Resistant Tuberculosis in Upper Northern Thailand. *Antibiotics (Basel)*. 2022;**11**(12). 10.3390/antibiotics11121733

116. Salehi M, Khalili H, Seifi A, Davoudi H, Darazam IA, Jahangard-Rafsanjani Z, et al. Antibiotic use during the first 6 months of COVID-19 pandemic in Iran: A large-scale multi-centre study. *J Clin Pharm Ther*. 2022;**47**(12):2140-51. 10.1111/jcpt.13761

117. Sardzikova S, Andrijkova K, Svec P, Beke G, Klucar L, Minarik G, et al. High Diversity but Monodominance of Multidrug-Resistant Bacteria in Immunocompromised Pediatric Patients with Acute Lymphoblastic Leukemia Developing GVHD Are Not Associated with Changes in Gut Mycobiome. *Antibiotics (Basel)*. 2023;**12**(12). 10.3390/antibiotics12121667

118. Sayan M, Sultanoglu N, Sanlidag T. Dynamics of Rilpivirine Resistance-Associated Mutation: E138 in Reverse Transcriptase among Antiretroviral-Naive HIV-1-Infected Individuals in Turkey. *AIDS Res Hum Retroviruses*. 2023;**39**(2):84-90. 10.1089/aid.2022.0065

119. Sertoz R, Tekin D, Erensoy S, Biceroglu S, Kaptan F, Köse S, et al. Prevalence of Transmitted Drug Resistance among HIV-1 Patients in the Aegean Region: Results from the Western Part of Turkey. *Curr HIV Res*. 2023;**21**(2):109-16. 10.2174/1570162x21666230525145529

120. Sharma D, Singh A. Editorial: Pathogenesis, diagnostics, treatments of Mycobacterium tuberculosis and its co-infection with HIV or SARS-CoV-2. *Front Cell Infect Microbiol*. 2024;**14**:1359356. 10.3389/fcimb.2024.1359356

121. Shipitsyna E, Kularatne R, Golparian D, Müller EE, Vargas SK, Hadad R, et al. Mycoplasma genitalium prevalence, antimicrobial resistance-associated mutations, and coinfections with non-viral sexually transmitted infections in high-risk populations in Guatemala, Malta, Morocco, Peru and South Africa, 2019-2021. *Front Microbiol*. 2023;**14**:1130762. 10.3389/fmicb.2023.1130762

122. Sokoll PR, Migliavaca CB, Siebert U, Schmid D, Arvandi M. Prevalence of Mycoplasma genitalium infection among HIV PrEP users: a systematic review and meta-analysis. *Sex Transm Infect*. 2023;**99**(5):351-9. 10.1136/sextrans-2022-055687

123. Sood R, Walo C, Burton R, Khalife M, Dicko A, Mangana F. Spontaneous nosocomial Proteus mirabilis meningitis in a Human Immunodeficiency Virus (HIV)-infected adult patient: a case report. *J Med Case Rep*. 2023;**17**(1):52. 10.1186/s13256-022-03704-0

124. Spielvogel E, Lee SK, Zhou S, Lockbaum GJ, Henes M, Sondgeroth A, et al. Selection of HIV-1 for resistance to fifth-generation protease inhibitors reveals two independent pathways to high-level resistance. *Elife*. 2023;**12**. 10.7554/eLife.80328

125. Spottiswoode N, Hao S, Sanchez-Guerrero E, Detweiler AM, Mekonen H, Neff N, et al. In host evolution of beta lactam resistance during active treatment for Pseudomonas aeruginosa bacteremia. *Front Cell Infect Microbiol*. 2023;**13**:1241608. 10.3389/fcimb.2023.1241608

126. Srivastava S, Singh P, Sharad N, Kiro VV, Malhotra R, Mathur P. Infection Trends, Susceptibility Pattern, and Treatment Options for Stenotrophomonas maltophilia Infections in Trauma Patients: A Retrospective Study. *J Lab Physicians*. 2023;**15**(1):106-9. 10.1055/s-0042-1757413

127. Stott KE, Ahmadu A, Kajanga C, Moyo M, Gondwe E, Chimang'anga W, et al. Population pharmacokinetics and CSF penetration of flucytosine in adults with HIV-associated cryptococcal meningoencephalitis. *J Antimicrob Chemother*. 2023;**78**(4):1015-22. 10.1093/jac/dkad038

128. Suprewicz Ł, Skłodowski K, Walewska A, Deptuła P, Sadzyńska A, Eljaszewicz A, et al. Plasma Gelsolin Enhances Phagocytosis of Candida auris by Human Neutrophils through Scavenger Receptor Class B. *Microbiol Spectr*. 2023;**11**(2):e0408222. 10.1128/spectrum.04082-22

129. Takem EN, Coox C, Shang J, Ndongmo C, Dokubo EK. The association between HIV pretreatment drug resistance and virological outcomes in children and adults in sub-Saharan Africa: A systematic review and meta-analysis. *PLoS One*. 2024;**19**(4):e0300456. 10.1371/journal.pone.0300456

130. Teker B, Schim van der Loeff M, Hoornenborg E, Boyd A, Reedijk S, van Dam A, et al. Minimum inhibitory concentrations of Neisseria gonorrhoeae strains in clients of the Amsterdam sexual health clinic with a Dutch versus an international sexual network. *Sex Transm Infect*. 2024;**100**(3):173-80. 10.1136/sextrans-2023-055988

131. Thangaraju P, Yella SST, Ramamurthy VA, Navabshan I, Mohamed T. A Perspective into "TEL"-Tenofovir, Emtricitabine and Lamivudine Antileprotic Activities by Drug Repurposing and Exploring the Possibility of Combination Chemotherapy with Drug Rescued Molecules for a Leprosy Free Mankind. *Recent Adv Antiinfect Drug Discov*. 2023;**18**(3):170-7. 10.2174/2772434418666230220123217

132. Thanyasrisung P, Satitviboon W, Howattanapanich S, Matangkasombut O. Antifungal drug resistance in oral Candida isolates from HIV-infected and healthy individuals and efficacy of chitosan as an alternative antifungal agent. *Arch Oral Biol*. 2023;**147**:105628. 10.1016/j.archoralbio.2023.105628

133. Tilahun M, Gebretsadik D, Seid A, Gedefie A, Belete MA, Tesfaye M, et al. Bacteriology of community-acquired pneumonia, antimicrobial susceptibility pattern and associated risk factors among HIV patients, Northeast Ethiopia: cross-sectional study. *SAGE Open Med*. 2023;**11**:20503121221145569. 10.1177/20503121221145569

134. Trunfio M, Pinnetti C, Arsuffi S, Bai F, Celani L, D'Ettorre G, et al. The presence of resistance-associated mutations in reverse transcriptase gene is associated with cerebrospinal fluid HIV-1 escape: A multicentric retrospective analysis. *J Med Virol*. 2023;**95**(4):e28704. 10.1002/jmv.28704

135. Valencia-Trujillo D, Avila-Trejo AM, García-Reyes RL, Narváez-Díaz L, Mújica-Sánchez MA, Helguera-Repetto AC, et al. Phenotypic and Genotypic Drug Resistance of Mycobacterium tuberculosis Strains Isolated from HIV-Infected Patients from a Third-Level Public Hospital in Mexico. *Pathogens*. 2024;**13**(2). 10.3390/pathogens13020098

136. van de Klundert MAA, Antonova A, Di Teodoro G, Ceña Diez R, Chkhartishvili N, Heger E, et al. Molecular Epidemiology of HIV-1 in Eastern Europe and Russia. *Viruses*. 2022;**14**(10). 10.3390/v14102099

137. Van Dijck C, Laumen JGE, de Block T, Abdellati S, De Baetselier I, Tsoumanis A, et al. The oropharynx of men using HIV pre-exposure prophylaxis is enriched with antibiotic resistance genes: A cross-sectional observational metagenomic study. *J Infect*. 2023;**86**(4):329-37. 10.1016/j.jinf.2023.02.006

138. van Kampen JJA, Pham HT, Yoo S, Overmars RJ, Lungu C, Mahmud R, et al. HIV-1 resistance against dolutegravir fluctuates rapidly alongside erratic treatment adherence: a case report. *J Glob Antimicrob Resist*. 2022;**31**:323-7. 10.1016/j.jgar.2022.11.001

139. Wahl A, Fischer MA, Klaper K, Müller A, Borgmann S, Friesen J, et al. Presence of hypervirulence-associated determinants in Klebsiella pneumoniae from hospitalised patients in Germany. *Int J Med Microbiol*. 2024;**314**:151601. 10.1016/j.ijmm.2024.151601

140. Wake RM, Molloy SF, Jarvis JN, Harrison TS, Govender NP. Cryptococcal Antigenemia in Advanced Human Immunodeficiency Virus Disease: Pathophysiology, Epidemiology, and Clinical Implications. *Clin Infect Dis*. 2023;**76**(4):764-70. 10.1093/cid/ciac675

141. Wang Y, Jing W, Liu J, Liu M. Global trends, regional differences and age distribution for the incidence of HIV and tuberculosis co-infection from 1990 to 2019: results from the global burden of disease study 2019. *Infect Dis (Lond)*. 2022;**54**(11):773-83. 10.1080/23744235.2022.2092647

142. Wardley AM, Williams H, Coombe J, Caddy C, Fairley CK, Hocking JS. Would men who have sex with men support less frequent screening for asymptomatic chlamydia and gonorrhoea to improve antibiotic stewardship? A qualitative study. *Sex Health*. 2023;**20**(2):148-57. 10.1071/sh22139

143. Wedderburn CJ, Evans C, Slogrove AL, Rehman AM, Gibb DM, Prendergast AJ, et al. Co-trimoxazole prophylaxis for children who are HIV-exposed and uninfected: a systematic review. *J Int AIDS Soc*. 2023;**26**(6):e26079. 10.1002/jia2.26079

144. Weldemhret L, Atsbaha AH, Bekuretsion H, Desta A, Legesse L, Kahsay AG, et al. Time to Sputum Culture Conversion and Its Predictors Among Multidrug Resistant Tuberculosis Patients in Tigray, Northern Ethiopia: Retrospective Cohort Study. *Infect Drug Resist*. 2023;**16**:3671-81. 10.2147/idr.S413495

145. Yoon H, Wake RM, Nakouzi AS, Wang T, Agalliu I, Tiemessen CT, et al. Association of Antibody Immunity With Cryptococcal Antigenemia and Mortality in a South African Cohort With Advanced Human Immunodeficiency Virus Disease. *Clin Infect Dis*. 2023;**76**(4):649-57. 10.1093/cid/ciac633

146. Zhang W, Yan CY, Li SR, Fan TT, Cao SS, Cui B, et al. Efficacy and safety of piperacillin-tazobactam compared with meropenem in treating complicated urinary tract infections including acute pyelonephritis due to extended-spectrum β-lactamase-producing Enterobacteriaceae. *Front Cell Infect Microbiol*. 2023;**13**:1093842. 10.3389/fcimb.2023.1093842

147. Zhang Y, Yu C, Jiang Y, Zheng X, Wang L, Li J, et al. Drug resistance profile of Mycobacterium kansasii clinical isolates before and after 2-month empirical antimycobacterial treatment. *Clin Microbiol Infect*. 2023;**29**(3):353-9. 10.1016/j.cmi.2022.10.002

148. Abdelnabi M, Cavazos A, Mittal N, Tarbox M. Leucocytoclastic vasculitis secondary to urinary tract infection caused by extended-spectrum β-lactamase producing Klebsiella pneumoniae. *BMJ Case Rep*. 2023;**16**(4). 10.1136/bcr-2023-255395

149. Abubaker KT, Anwar KA. Antimicrobial susceptibility and integrons detection among extended-spectrum β-lactamase producing Enterobacteriaceae isolates in patients with urinary tract infection. *PeerJ*. 2023;**11**:e15429. 10.7717/peerj.15429

150. Adekanmbi AO, Akinlabi OC, Usidamen S, Olaposi AV, Olaniyan AB. High burden of ESBL- producing Klebsiella spp., Proteus mirabilis, Enterobacter cloacae and Pseudomonas aeruginosa in diagnosed cases of urinary tract infection in a Nigerian Teaching Hospital. *Acta Microbiol Immunol Hung*. 2022;**69**(2):127-34. 10.1556/030.2022.01747

151. Afsharikhah S, Ghanbarpour R, Mohseni P, Adib N, Bagheri M, Jajarmi M. High prevalence of β-lactam and fluoroquinolone resistance in various phylotypes of Escherichia coli isolates from urinary tract infections in Jiroft city, Iran. *BMC Microbiol*. 2023;**23**(1):114. 10.1186/s12866-023-02860-7

152. Aghamohammad S, Nikbin VS, Badmasti F, Shahcheraghi F. High heterogeneity of fecal carriage extended-spectrum beta-lactamase-producing E. coli isolated from iranian community and clinical settings. *BMC Infect Dis*. 2022;**22**(1):318. 10.1186/s12879-022-07304-7

153. Aghamohammad S, Shahcheraghi F. The notable relatedness between ESBL producing Enterobacteriaceae isolated from clinical samples and asymptomatic fecal carriers. *BMC Infect Dis*. 2023;**23**(1):775. 10.1186/s12879-023-08746-3

154. Aguilar-Bultet L, García-Martín AB, Vock I, Maurer Pekerman L, Stadler R, Schindler R, et al. Within-host genetic diversity of extended-spectrum beta-lactamase-producing Enterobacterales in long-term colonized patients. *Nat Commun*. 2023;**14**(1):8495. 10.1038/s41467-023-44285-w

155. Ahn ST, Lee HS, Han DE, Lee DH, Kim JW, Park MG, et al. What are the risk factors for recurrent UTI with repeated ESBL-producing Enterobacteriaceae? A retrospective cohort study. *J Infect Chemother*. 2023;**29**(1):72-7. 10.1016/j.jiac.2022.09.020

156. Ajimuda OE, Sanmi-Kayode I, Adeniyi OO, Alaka OO, Onipede A. Prevalence of extended spectrum Beta-Lactamase producing Klebsiella species from patients' specimens in a tertiary teaching hospital in Ile-Ife, Southwest Nigeria. *Afr Health Sci*. 2022;**22**(2):146-55. 10.4314/ahs.v22i2.17

157. Akenten CW, Khan NA, Mbwana J, Krumkamp R, Fosu D, Paintsil EK, et al. Carriage of ESBL-producing Klebsiella pneumoniae and Escherichia coli among children in rural Ghana: a cross-sectional study. *Antimicrob Resist Infect Control*. 2023;**12**(1):60. 10.1186/s13756-023-01263-7

158. Alotibi I, Al-Sarraj F, Albiheyri R, Alghamdi MA, Nass N, Bouback T, et al. Study the apoptosis and necrosis inducing of fosfomycin into associated infected urothelial tissue by extended spectrum beta lactamase positive of E. coli. *Microb Pathog*. 2022;**173**(Pt A):105838. 10.1016/j.micpath.2022.105838

159. Al-Sheboul SA, Al-Madi GS, Brown B, Hayajneh WA. Prevalence of Extended-Spectrum β-Lactamases in Multidrug-Resistant Klebsiella pneumoniae Isolates in Jordanian Hospitals. *J Epidemiol Glob Health*. 2023;**13**(2):180-90. 10.1007/s44197-023-00096-2

160. Alsubaie MA, Alsuheili AZ, Aljehani MN, Alothman AA, Alzahrani AS, Mohammedfadel HA, et al. Pediatric community acquired urinary tract infections due to extended-spectrum beta-lactamase versus non-extended-spectrum beta-lactamase producing bacteria. *Pediatr Int*. 2023;**65**(1):e15620. 10.1111/ped.15620

161. Amare A, Eshetie S, Kasew D, Moges F. High prevalence of fecal carriage of Extended-spectrum beta-lactamase and carbapenemase-producing Enterobacteriaceae among food handlers at the University of Gondar, Northwest Ethiopia. *PLoS One*. 2022;**17**(3):e0264818. 10.1371/journal.pone.0264818

162. Amato HK, Loayza F, Salinas L, Paredes D, Garcia D, Sarzosa S, et al. Risk factors for extended-spectrum beta-lactamase (ESBL)-producing E. coli carriage among children in a food animal-producing region of Ecuador: A repeated measures observational study. *PLoS Med*. 2023;**20**(10):e1004299. 10.1371/journal.pmed.1004299

163. Aramwittayanukul S, Malathum K, Kantachuvesiri S, Arpornsujaritkun N, Chootip P, Bruminhent J. Impact of Carbapenem Peri-Transplant Prophylaxis and Risk of Extended-Spectrum Cephalosporin-Resistant Enterobacterales Early Urinary Tract Infection in Kidney Transplant Recipients: A Propensity Score-Matched Analysis. *Front Med (Lausanne)*. 2022;**9**:841293. 10.3389/fmed.2022.841293

164. Araújo MRB, Sant'Anna LO, Santos N, Seabra LF, Santos LSD. Monitoring fluoroquinolone resistance among ESBL-positive and ESBL-negative Escherichia coli strains isolated from urinary tract infections: An alert for empirical treatment. *Rev Soc Bras Med Trop*. 2023;**56**:e0513. 10.1590/0037-8682-0513-2022

165. Arias Ramos D, Alzate JA, Moreno Gómez GA, Hoyos Pulgarín JA, Olaya Gómez JC, Cortés Bonilla I, et al. Empirical treatment and mortality in bacteremia due to extended spectrum β-lactamase producing Enterobacterales (ESβL-E), a retrospective cross-sectional study in a tertiary referral hospital from Colombia. *Ann Clin Microbiol Antimicrob*. 2023;**22**(1):13. 10.1186/s12941-023-00566-2

166. Aronin SI, Dunne MW, Yu KC, Watts JA, Gupta V. Increased rates of extended-spectrum beta-lactamase isolates in patients hospitalized with culture-positive urinary Enterobacterales in the United States: 2011 - 2020. *Diagn Microbiol Infect Dis*. 2022;**103**(4):115717. 10.1016/j.diagmicrobio.2022.115717

167. Awoke T, Teka B, Aseffa A, Seman A, Sebre S, Yitayew B, et al. Magnitude and Molecular Characterization of Extended-Spectrum β-Lactamase Genes among Klebsiella pneumoniae Isolates in a Large Tertiary Hospital in Ethiopia. *Adv Exp Med Biol*. 2023;**1370**:91-102. 10.1007/5584_2022_739

168. Begaj X, Lee H, Noor A, Fiorito T, Agarwalla V, Kambhampati O, et al. Clinical Outcomes of Children With Extended-spectrum ß -Lactamase Urinary Tract Infection Receiving Discordant Empiric Antibiotic: A Comparative Study of Fever Duration, Length of Stay, and Readmissions. *Clin Pediatr (Phila)*. 2023;**62**(4):338-44. 10.1177/00099228221129029

169. Ben Hassena A, Guermazi-Toumi S, Gdoura-Ben Amor M, Saidani M, Tlili S, Khannous L, et al. Detection of AmpC and ESBL-producing Enterobacterales isolated from urinary tract infections in Tunisia. *Acta Microbiol Immunol Hung*. 2022. 10.1556/030.2022.01630

170. Ben Sallem R, Laribi B, Arfaoui A, Ben Khelifa Melki S, Ouzari HI, Ben Slama K, et al. Co-occurrence of genes encoding carbapenemase, ESBL, pAmpC and non-β-Lactam resistance among Klebsiella pneumonia and E. coli clinical isolates in Tunisia. *Lett Appl Microbiol*. 2022;**74**(5):729-40. 10.1111/lam.13658

171. Beshah D, Desta AF, Woldemichael GB, Belachew EB, Derese SG, Zelelie TZ, et al. High burden of ESBL and carbapenemase-producing gram-negative bacteria in bloodstream infection patients at a tertiary care hospital in Addis Ababa, Ethiopia. *PLoS One*. 2023;**18**(6):e0287453. 10.1371/journal.pone.0287453

172. Bier N, Hanson B, Jiang ZD, DuPont HL, Arias CA, Miller WR. A Case of Successful Treatment of Recurrent Urinary Tract Infection by Extended-Spectrum β-Lactamase Producing Klebsiella pneumoniae Using Oral Lyophilized Fecal Microbiota Transplant. *Microb Drug Resist*. 2023;**29**(1):34-8. 10.1089/mdr.2022.0031

173. Bizimana J, Ndayisenga J, Kajumbura H, Mulepo P, Christine NF. Colonization of patients hospitalized at orthopedic department of tertiary hospital in Uganda with extended-spectrum beta-lactamase-producing enterobacterales. *Antimicrob Resist Infect Control*. 2023;**12**(1):26. 10.1186/s13756-023-01229-9

174. Boattini M, Bianco G, Comini S, Iannaccone M, Casale R, Cavallo R, et al. Direct detection of extended-spectrum-β-lactamase-producers in Enterobacterales from blood cultures: a comparative analysis. *Eur J Clin Microbiol Infect Dis*. 2022;**41**(3):407-13. 10.1007/s10096-021-04385-1

175. Bobbadi S, Bobby MN, Chinnam BK, Reddy PN, Kandhan S. Phenotypic and genetic screening of Klebsiella pneumoniae isolates from human UTI patients for beta-lactamases and their genetic diversity analysis by ERIC and REP PCRs. *Braz J Microbiol*. 2023;**54**(3):1723-36. 10.1007/s42770-023-00984-6

176. Bouchand C, Andréo A, Le Gallou F, Corvec S, Bourigault C, Lepelletier D. Retrospective analysis of a large single cohort of Enterobacteriaceae producing extended-spectrum B-lactamase (E-ESBL) patients: incidence, microbiology, and mortality. *Eur J Clin Microbiol Infect Dis*. 2022;**41**(10):1237-43. 10.1007/s10096-022-04489-2

177. Boussetta A, Kharbach N, Abdellatif A, Karray A, Jellouli M, Gargah T. Predictive factors of urinary tract infections caused by extended-spectrum β-lactamase-producing Escherichia coli in children: a prospective Tunisian study. *Tunis Med*. 2023;**101**(2):285-91.

178. Brown DG, Worby CJ, Pender MA, Brintz BJ, Ryan ET, Sridhar S, et al. Development of a prediction model for the acquisition of extended spectrum beta-lactam-resistant organisms in U.S. international travellers. *J Travel Med*. 2023;**30**(6). 10.1093/jtm/taad028

179. Camargo CH, Yamada AY, de Souza AR, Sacchi CT, Reis AD, Santos MBN, et al. Genomic characterization of New Delhi metallo-beta-lactamase-producing species of Morganellaceae, Yersiniaceae, and Enterobacteriaceae (other than Klebsiella) from Brazil over 2013-2022. *Microbiol Immunol*. 2024;**68**(1):1-5. 10.1111/1348-0421.13100

180. Campos-Madueno EI, Moradi M, Eddoubaji Y, Shahi F, Moradi S, Bernasconi OJ, et al. Intestinal colonization with multidrug-resistant Enterobacterales: screening, epidemiology, clinical impact, and strategies to decolonize carriers. *Eur J Clin Microbiol Infect Dis*. 2023;**42**(3):229-54. 10.1007/s10096-023-04548-2

181. Cave R, Ter-Stepanyan MM, Kotsinyan N, Mkrtchyan HV. An Emerging Lineage of Uropathogenic Extended Spectrum β-Lactamase Escherichia coli ST127. *Microbiol Spectr*. 2022;**10**(6):e0251122. 10.1128/spectrum.02511-22

182. Chan EY. Community-acquired urinary tract infections caused by ESBL-producing Enterobacteriaceae in infants less than 2 years of age. *Pediatr Nephrol*. 2022;**37**(5):1167-8. 10.1007/s00467-022-05443-2

183. Chaudhary MK, Jadhav I, Banjara MR. Molecular detection of plasmid mediated bla(TEM), bla(CTX-M,)and bla(SHV) genes in Extended Spectrum β-Lactamase (ESBL) Escherichia coli from clinical samples. *Ann Clin Microbiol Antimicrob*. 2023;**22**(1):33. 10.1186/s12941-023-00584-0

184. Chen L, Hua J, Hong SJ, Yuan CY, Jing RC, Luo XY, et al. Comparison of the relative efficacy of β-lactam/β-lactamase inhibitors and carbapenems in the treatment of complicated urinary tract infections caused by ceftriaxone-non-susceptible Enterobacterales: a multicentre retrospective observational cohort study. *J Antimicrob Chemother*. 2023;**78**(3):710-8. 10.1093/jac/dkac448

185. Chen WL, Cheng MF, Tseng FC, Wu PC, Huang IF, Chen YW, et al. Geographic distribution of the major clone of extended-spectrum beta-lactamase-producing Escherichia coli infection in a pediatric community in southern Taiwan. *J Infect Public Health*. 2023;**16**(6):853-8. 10.1016/j.jiph.2023.03.017

186. Chen YC, Chen WY, Hsu WY, Tang HJ, Chou Y, Chang YH, et al. Distribution of β-lactamases and emergence of carbapenemases co-occurring Enterobacterales isolates with high-level antibiotic resistance identified from patients with intra-abdominal infection in the Asia-Pacific region, 2015-2018. *J Microbiol Immunol Infect*. 2022;**55**(6 Pt 2):1263-72. 10.1016/j.jmii.2021.07.007

187. Cheng MF, Ho PY, Wang JL, Tseng FC, Chang JT, Huang IF, et al. Prevalence and household risk factors for fecal carriage of ESBL-producing, sequence type 131, and extraintestinal pathogenic Escherichia coli among children in southern Taiwan. *J Microbiol Immunol Infect*. 2022;**55**(4):695-707. 10.1016/j.jmii.2022.04.001

188. Choi YS, Kim JH, Kim Y, Cho HJ, Sung JH, Choi SJ, et al. Growing threat of extended-spectrum β-lactamase-producing Enterobacteriaceae colonisation in high-risk pregnancies: A cross-sectional study. *Bjog*. 2023;**130**(4):415-23. 10.1111/1471-0528.17194

189. Choudhury K, Dhar Chanda D, Bhattacharjee A. Molecular characterization of extended spectrum beta lactamases in clinical isolates of Escherichia coli and Klebsiella spp from a tertiary care hospital of South Eastern Assam. *Indian J Med Microbiol*. 2022;**40**(1):135-7. 10.1016/j.ijmmb.2021.11.001

190. Chuang C, Lee KC, Wang YP, Lee PC, Chang TE, Huang YH, et al. High carriage rate of extended-spectrum β-lactamase Enterobacterales and diarrheagenic Escherichia coli in healthy donor screening for fecal microbiota transplantation. *Eur J Clin Microbiol Infect Dis*. 2023;**42**(9):1103-13. 10.1007/s10096-023-04644-3

191. Chumbita M, Puerta-Alcalde P, Yáñez L, Cuesta MA, Chinea A, Español Morales I, et al. Resistance to empirical β-lactams recommended in febrile neutropenia guidelines in Gram-negative bacilli bloodstream infections in Spain: a multicentre study. *J Antimicrob Chemother*. 2022;**77**(7):2017-23. 10.1093/jac/dkac135

192. Collingwood JD, Wang L, Aban IB, Yarbrough AH, Boppana SB, Dangle PP. Risk factors for community acquired pediatric urinary tract infection with extended-spectrum-β-lactamase Escherichia coli - A case-control study. *J Pediatr Urol*. 2023;**19**(1):129.e1-.e7. 10.1016/j.jpurol.2022.10.020

193. Collingwood JD, Yarbrough AH, Boppana SB, Dangle PP. Increasing Prevalence of Pediatric Community-acquired UTI by Extended Spectrum β-Lactamase-producing E. coli: Cause for Concern. *Pediatr Infect Dis J*. 2023;**42**(2):106-9. 10.1097/inf.0000000000003777

194. Comini S, Bianco G, Boattini M, Banche G, Ricciardelli G, Allizond V, et al. Evaluation of a diagnostic algorithm for rapid identification of Gram-negative species and detection of extended-spectrum β-lactamase and carbapenemase directly from blood cultures. *J Antimicrob Chemother*. 2022;**77**(10):2632-41. 10.1093/jac/dkac230

195. de Korne-Elenbaas J, van der Putten BCL, Boek NDM, Matser A, Schultsz C, Bruisten SM, et al. Putative transmission of extended-spectrum β-lactamase-producing Escherichia coli among men who have sex with men in Amsterdam, the Netherlands. *Int J Antimicrob Agents*. 2023;**62**(1):106810. 10.1016/j.ijantimicag.2023.106810

196. de Lauzanne A, Sreng N, Foucaud E, Sok T, Chon T, Yem C, et al. Prevalence and factors associated with faecal carriage of extended-spectrum β-lactamase-producing Enterobacterales among peripartum women in the community in Cambodia. *J Antimicrob Chemother*. 2022;**77**(10):2658-66. 10.1093/jac/dkac224

197. Dequidt T, Bastian S, Nacher M, Breurec S, Carles M, Thiery G, et al. Cefoxitin versus carbapenems as definitive treatment for extended-spectrum β-lactamase-producing Klebsiella pneumoniae bacteremia in intensive care unit: a propensity-matched retrospective analysis. *Crit Care*. 2023;**27**(1):418. 10.1186/s13054-023-04712-2

198. Djim-Adjim-Ngana K, Mbiakop BW, Oumar LA, Munshili Njifon HL, Tchinda Fossi C, Enyegue ELE, et al. Phenotypic characterization and epidemiology of extended-spectrum β-lactamase-producing Enterobacteriaceae strains from urinary tract infections in Garoua, Cameroon. *Front Public Health*. 2023;**11**:1187934. 10.3389/fpubh.2023.1187934

199. Doerr N, Dietze N, Lippmann N, Rodloff AC. Extended-spectrum beta-lactamases found in Escherichia coli isolates obtained from blood cultures and corresponding stool specimen. *Sci Rep*. 2023;**13**(1):8940. 10.1038/s41598-023-36240-y

200. Ducarmon QR, Zwittink RD, Willems RPJ, Verhoeven A, Nooij S, van der Klis FRM, et al. Gut colonisation by extended-spectrum β-lactamase-producing Escherichia coli and its association with the gut microbiome and metabolome in Dutch adults: a matched case-control study. *Lancet Microbe*. 2022;**3**(6):e443-e51. 10.1016/s2666-5247(22)00037-4

201. Duffy N, Karlsson M, Reses HE, Campbell D, Daniels J, Stanton RA, et al. Epidemiology of extended-spectrum β-lactamase-producing Enterobacterales in five US sites participating in the Emerging Infections Program, 2017. *Infect Control Hosp Epidemiol*. 2022;**43**(11):1586-94. 10.1017/ice.2021.496

202. Duran-Bedolla J, Garza-Ramos U, Silva-Sánchez J, Lozano L, Reyna-Flores F, Sánchez-Pérez A, et al. Genetic characterization of plasmid-mediated fluoroquinolone efflux pump QepA among ESBL-producing Escherichia coli isolates in Mexico. *Braz J Microbiol*. 2023;**54**(4):2791-7. 10.1007/s42770-023-01115-x

203. Edwards T, Williams CT, Olwala M, Andang'o P, Otieno W, Nalwa GN, et al. Molecular surveillance reveals widespread colonisation by carbapenemase and extended spectrum beta-lactamase producing organisms in neonatal units in Kenya and Nigeria. *Antimicrob Resist Infect Control*. 2023;**12**(1):14. 10.1186/s13756-023-01216-0

204. Ehsan B, Haque A, Qasim M, Ali A, Sarwar Y. High prevalence of extensively drug resistant and extended spectrum beta lactamases (ESBLs) producing uropathogenic Escherichia coli isolated from Faisalabad, Pakistan. *World J Microbiol Biotechnol*. 2023;**39**(5):132. 10.1007/s11274-023-03565-9

205. El Aila NA, Al Laham NA, Ayesh BM. Prevalence of extended spectrum beta lactamase and molecular detection of blaTEM, blaSHV and blaCTX-M genotypes among Gram negative bacilli isolates from pediatric patient population in Gaza strip. *BMC Infect Dis*. 2023;**23**(1):99. 10.1186/s12879-023-08017-1

206. El Aila NA, Laham NAA, Ayesh BM, Naas T. Fecal carriage of extended-spectrum β-lactamase-producing enterobacterales from hospitals and community settings in Gaza Strip, Palestine. *BMC Microbiol*. 2023;**23**(1):376. 10.1186/s12866-023-03102-6

207. El-Masry EA, Alruwaili FM, Taha AE, Saad AE, Taher IA. Prevalence of extended-spectrum beta-lactamase-producing Enterobacteriaceae among clinical isolates in Turaif general hospital, northern borders- Saudi Arabia. *J Infect Dev Ctries*. 2023;**17**(4):477-84. 10.3855/jidc.17212

208. Enyinnaya SO, Iregbu KC, Jamal WY, Rotimi VO. Antibiotic susceptibility profiles and detection of genes mediating extended-spectrum β-lactamase (Esbl) production in escherichia coli isolates from National Hospital, Abuja. *Niger J Clin Pract*. 2022;**25**(8):1216-20. 10.4103/njcp.njcp_1390_21

209. Fakorede CO, Amisu KO, Saki M, Akinyemi KO. Co-existence of extended-spectrum β-lactamases bla(CTX-M-9) and bla(CTX-M-15) genes in Salmonella species isolated from febrile and diarrhoeagenic patients in Lagos, Nigeria: a cross-sectional study. *Eur J Med Res*. 2023;**28**(1):3. 10.1186/s40001-022-00960-0

210. Fernández Vecilla D, Zugazaga Inchaurza K, Lombide Aguirre I, Díaz de Tuesta Del Arco JL. Phenotypic and genotypic characterization of Shigella sonnei carrying the extended-spectrum beta-lactamase CTX-M-27. A report of two cases in Spain in men who have sex with men. *Enferm Infecc Microbiol Clin (Engl Ed)*. 2023;**41**(4):248-50. 10.1016/j.eimce.2023.02.003

211. Ford CD, Lopansri BK, Coombs J, Gouw L, Asch J, Hoda D. Extended spectrum cephalosporin resistant enterobacteriaceae carriage and infection in patients admitted with newly-diagnosed acute leukemia. *Am J Infect Control*. 2023;**51**(2):172-7. 10.1016/j.ajic.2022.05.019

212. Furuichi M, Yaginuma M, Shinjoh M, Ohnishi T, Takahashi T, Iwata S. Extended-Spectrum β-Lactamase-Producing Escherichia coli in Neonates and Listeria monocytogenes in Young Children with Bacterial Meningitis in Japan. *J Pediatric Infect Dis Soc*. 2023;**12**(3):165-8. 10.1093/jpids/piac135

213. Gainey AB, Daniels R, Burch AK, Hawn J, Fackler J, Biswas B, et al. Recurrent ESBL Escherichia coli Urosepsis in a Pediatric Renal Transplant Patient Treated With Antibiotics and Bacteriophage Therapy. *Pediatr Infect Dis J*. 2023;**42**(1):43-6. 10.1097/inf.0000000000003735

214. Gallah S, Scherer M, Collin T, Gomart C, Veziris N, Benzerara Y, et al. Five-Hour Detection of Intestinal Colonization with Extended-Spectrum-β-Lactamase-Producing Enterobacteriaceae Using the β-Lacta Phenotypic Test: the BLESSED Study. *Microbiol Spectr*. 2023;**11**(1):e0295922. 10.1128/spectrum.02959-22

215. Garba Z, Kaboré B, Bonkoungou IJO, Natama MH, Rouamba T, Haukka K, et al. Phenotypic Detection of Carbapenemase and AmpC-β-Lactamase Production among Extended Spectrum β-Lactamase (ESBL)-Producing Escherichia coli and Klebsiella spp. Isolated from Clinical Specimens. *Antibiotics (Basel)*. 2023;**13**(1). 10.3390/antibiotics13010031

216. Ghosh A, Ghosh B, Mukherjee M. Epidemiologic and molecular characterization of β-lactamase-producing multidrug-resistant uropathogenic Escherichia coli isolated from asymptomatic hospitalized patients. *Int Microbiol*. 2022;**25**(1):27-45. 10.1007/s10123-021-00187-9

217. Gonzales-Rodriguez AO, Infante Varillas SF, Reyes-Farias CI, Ladines Fajardo CE, Gonzales Escalante E. Extended-spectrum β-lactamases and virulence factors in uropathogenic Escherichia coli in nursing homes in Lima, Peru. *Rev Peru Med Exp Salud Publica*. 2022;**39**(1):98-103. 10.17843/rpmesp.2022.391.8580

218. Grall-Zahar I, Rucly S, Billard-Pomares T, Gasnier-Besnardeau K, Al Mouft O, Zahar JR, et al. Prevalence and risk factors for carriage of extended-spectrum β-lactamase-producing enterobacteriaceae in rehabilitation wards in France. *Infect Dis Now*. 2022;**52**(7):403-7. 10.1016/j.idnow.2022.07.004

219. Grohs P, Vilfaillot A, Zahar JR, Barbut F, Frange P, Casetta A, et al. Faecal carriage of multidrug-resistant bacteria and associated risk factors: results from a point prevalence study. *J Antimicrob Chemother*. 2022;**77**(10):2667-78. 10.1093/jac/dkac289

220. Habibzadeh N, Peeri Doghaheh H, Manouchehri Far M, Alimohammadi Asl H, Iranpour S, Arzanlou M. Fecal Carriage of Extended-Spectrum β-Lactamases and pAmpC Producing Enterobacterales in an Iranian Community: Prevalence, Risk Factors, Molecular Epidemiology, and Antibiotic Resistance. *Microb Drug Resist*. 2022;**28**(9):921-34. 10.1089/mdr.2021.0029

221. Hagiya H, Onishi Y, Shinohara N, Tokuyasu M, Imanishi A, Fukushima S, et al. High frequency of extended-spectrum beta-lactamase-producing Enterobacteriaceae carriers at a Japanese long-term care hospital. *J Infect Chemother*. 2022;**28**(11):1578-81. 10.1016/j.jiac.2022.07.014

222. Hajihasani A, Ebrahimi-Rad M, Rasoulinasab M, Aslani MM, Shahcheraghi F. Prevalence of O25b-ST131 Escherichia coli Clone: Fecal Carriage of Extended-Spectrum β-Lactamase and Carbapenemase-Producing Isolates in Healthy Adults in Tehran, Iran. *Microb Drug Resist*. 2022;**28**(2):210-6. 10.1089/mdr.2021.0001

223. Hamwi AM, Salem-Sokhn E. High frequency and molecular characterization of ESBL-producing Enterobacteriaceae isolated from wound infections in North Lebanon. *Expert Rev Anti Infect Ther*. 2023;**21**(8):901-9. 10.1080/14787210.2023.2234082

224. Handal N, Whitworth J, Lyngbakken MN, Berdal JE, Dalgard O, Bakken Jørgensen S. Mortality and length of hospital stay after bloodstream infections caused by ESBL-producing compared to non-ESBL-producing E. coli. *Infect Dis (Lond)*. 2024;**56**(1):19-31. 10.1080/23744235.2023.2261538

225. Harbaoui S, Ferjani S, Abbassi MS, Saidani M, Gargueh T, Ferjani M, et al. Genetic heterogeneity and predominance of bla(CTX-M) (-15) in cefotaxime-resistant Enterobacteriaceae isolates colonizing hospitalized children in Tunisia. *Lett Appl Microbiol*. 2022;**75**(6):1460-74. 10.1111/lam.13812

226. Harimanana A, Rakotondrasoa A, Rivoarilala LO, Criscuolo A, Opatowski L, Rakotomanana EFN, et al. Neonatal acquisition of extended-spectrum beta-lactamase-producing Enterobacteriaceae in the community of a low-income country (NeoLIC): protocol for a household cohort study in Moramanga, Madagascar. *BMJ Open*. 2022;**12**(9):e061463. 10.1136/bmjopen-2022-061463

227. Hasan MR, Vincent YM, Leto D, Almohri H. Trends in the Rates of Extended-Spectrum-β-Lactamase-Producing Enterobacterales Isolated from Urine Cultures during the COVID-19 Pandemic in Ontario, Canada. *Microbiol Spectr*. 2023;**11**(1):e0312422. 10.1128/spectrum.03124-22

228. Herrera S, Torralbo B, Herranz S, Bernal-Maurandi J, Rubio E, Pitart C, et al. Carriage of multidrug-resistant Gram-negative bacilli: duration and risk factors. *Eur J Clin Microbiol Infect Dis*. 2023;**42**(5):631-8. 10.1007/s10096-023-04581-1

229. Himmelsbach V, Knabe M, Ferstl PG, Peiffer KH, Stratmann JA, Wichelhaus TA, et al. Colonization with multidrug-resistant organisms impairs survival in patients with hepatocellular carcinoma. *J Cancer Res Clin Oncol*. 2022;**148**(6):1465-72. 10.1007/s00432-021-03741-0

230. Hoellinger B, Kaeuffer C, Boyer P, Lefebvre N, Hansmann Y, Robert A, et al. Cefepime vs carbapenems for treating third-generation cephalosporin-resistant AmpC β-lactamase-hyperproducing Enterobacterales bloodstream infections: a multicenter retrospective study. *Int J Infect Dis*. 2023;**134**:273-9. 10.1016/j.ijid.2023.07.004

231. Holmbom M, Möller V, Kristinsdottir L, Nilsson M, Rashid MU, Fredrikson M, et al. Risk factors and outcome due to extended-spectrum β-lactamase-producing uropathogenic Escherichia coli in community-onset bloodstream infections: A ten-year cohort study in Sweden. *PLoS One*. 2022;**17**(11):e0277054. 10.1371/journal.pone.0277054

232. Hounmanou YMG, Wanyana A, Alafi S, Wabwire-Mangen F, Christensen H, Olsen JE, et al. Whole strains vs MGEs in short and longterm transmission of ESBL genes between healthcare and community settings in Uganda. *Sci Rep*. 2023;**13**(1):10229. 10.1038/s41598-023-35879-x

233. Ibadin EE, Omoregie R, Enabulele IOJNZJoMLS. Prevalence of extended spectrum beta-lactamase, AmpC beta-lactamase and metallo-beta-lactamase enzymes among clinical isolates recovered from patients with urinary tract infections in Benin City, Nigeria. 2018;**72**(1):11-6.

234. Ibiam FA, Egwu E, Moses IB, Iroha CS, Adekwu A, Obasikene G, et al. Antibiogram and molecular characterization of extended-spectrum β-lactamase-producing pathogens implicated in chronic suppurative otitis media. *Pan Afr Med J*. 2023;**46**:108. 10.11604/pamj.2023.46.108.41156

235. Ilmavirta H, Ollgren J, Räisänen K, Kinnunen T, Hakanen AJ, Jalava J, et al. Increasing proportions of extended-spectrum β-lactamase-producing isolates among Escherichia coli from urine and bloodstream infections: results from a nationwide surveillance network, Finland, 2008 to 2019. *Euro Surveill*. 2023;**28**(43). 10.2807/1560-7917.Es.2023.28.43.2200934

236. Issa N, Coppry M, Ripoche E, Guisset O, Mourissoux G, Bessede E, et al. Impact of extended-spectrum beta-lactamase-producing Enterobacterales (ESBL-E) rectal carriage in cancer patients admitted to the intensive care unit. *Infect Dis Now*. 2022;**52**(2):104-6. 10.1016/j.idnow.2021.12.004

237. Jacquier H, Assao B, Chau F, Guindo O, Condamine B, Magnan M, et al. Faecal carriage of extended-spectrum β-lactamase-producing Escherichia coli in a remote region of Niger. *J Infect*. 2023;**87**(3):199-209. 10.1016/j.jinf.2023.06.015

238. Jiménez-Rojas V, Villanueva-García D, Miranda-Vega AL, Aldana-Vergara R, Aguilar-Rodea P, López-Marceliano B, et al. Gut colonization and subsequent infection of neonates caused by extended-spectrum beta-lactamase-producing Escherichia coli and Klebsiella pneumoniae. *Front Cell Infect Microbiol*. 2023;**13**:1322874. 10.3389/fcimb.2023.1322874

239. Kallel H, Houcke S, Resiere D, Court T, Roncin C, Raad M, et al. Prior Carriage Predicts Intensive Care Unit Infections Caused by Extended-Spectrum Beta-Lactamase-Producing Enterobacteriaceae. *Am J Trop Med Hyg*. 2022;**106**(2):525-31. 10.4269/ajtmh.20-1436

240. Kavruk M, Soyaltın E, Erfidan G, Arslansoyu Çamlar S, Alaygut D, Mutlubaş F, et al. The Influence of Non-E. Coli or Extended-Spectrum β-Lactamase-Producing Bacterial Growth on the Follow-Up Procedure of Infants with the First Febrile Urinary Tract Infection. *Indian J Pediatr*. 2023;**90**(7):677-82. 10.1007/s12098-022-04183-3

241. Kawata S, Morimoto S, Kosai K, Kawamoto Y, Nakashima Y, Morinaga Y, et al. The fecal carriage rate of extended-spectrum β-lactamase-producing or carbapenem-resistant Enterobacterales among Japanese infants in the community at the 4-month health examination in a rural city. *Front Cell Infect Microbiol*. 2023;**13**:1168451. 10.3389/fcimb.2023.1168451

242. Kayaaslan B, Oktay Z, Hasanoglu I, Kalem AK, Eser F, Ayhan M, et al. Increasing rates of extended-spectrum B-lactamase-producing Escherichia coli and Klebsiella pneumoniae in uncomplicated and complicated acute pyelonephritis and evaluation of empirical treatments based on culture results. *Eur J Clin Microbiol Infect Dis*. 2022;**41**(3):421-30. 10.1007/s10096-021-04392-2

243. Khaldi Z, Nayme K, Bourjilat F, Bensaci A, Timinouni M, Ould El-Hadj-Khelil A. Detection of ESBLs and carbapenemases among Enterobacteriaceae isolated from diabetic foot infections in Ouargla, Algeria. *J Infect Dev Ctries*. 2022;**16**(11):1732-8. 10.3855/jidc.16660

244. Kharaghani AA, Harzandi N, Khorsand B, Rajabnia M, Kharaghani AA, Houri H. High prevalence of Mucosa-Associated extended-spectrum β-Lactamase-producing Escherichia coli and Klebsiella pneumoniae among Iranain patients with inflammatory bowel disease (IBD). *Ann Clin Microbiol Antimicrob*. 2023;**22**(1):86. 10.1186/s12941-023-00630-x

245. Kharat AA, Makwana N, Kadam DG, Chavan AS, Kulkarni JA, Kharat AS. Co-existence of Extended Spectrum β-Lactamase and carbapenemase-producing genes from Diarrheagenic Enteric pathogens isolated in a tertiary care hospital. *Acta Biochim Pol*. 2023;**70**(1):69-76. 10.18388/abp.2020_6188

246. Khoshbayan A, Golmoradi Zadeh R, Taati Moghadam M, Mirkalantari S, Darbandi A. Molecular determination of O25b/ST131 clone type among extended spectrum β-lactamases production Escherichia coli recovering from urinary tract infection isolates. *Ann Clin Microbiol Antimicrob*. 2022;**21**(1):35. 10.1186/s12941-022-00526-2

247. Kibwana UO, Manyahi J, Sandnes HH, Blomberg B, Mshana SE, Langeland N, et al. Gastrointestinal colonization of extended-spectrum beta-lactamase-producing bacteria among children below five years of age hospitalized with fever in Dar es Salaam, Tanzania. *J Glob Antimicrob Resist*. 2022;**30**:107-14. 10.1016/j.jgar.2022.05.023

248. Kibwana UO, Manyahi J, Sandnes HH, Blomberg B, Mshana SE, Langeland N, et al. Fluoroquinolone resistance among fecal extended spectrum βeta lactamases positive Enterobacterales isolates from children in Dar es Salaam, Tanzania. *BMC Infect Dis*. 2023;**23**(1):135. 10.1186/s12879-023-08086-2

249. Kikuchi M, Suzuki Y, Okada S, Sato A, Oshima K, Matsumoto T. Septic shock caused by a carbon dioxide-dependent and extended spectrum β-lactamase-producing Proteus mirabilis small colony variant in a long-term bedridden patient. *J Infect Chemother*. 2022;**28**(3):455-8. 10.1016/j.jiac.2021.12.013

250. Kohler P, Seiffert SN, Kessler S, Rettenmund G, Lemmenmeier E, Qalla Widmer L, et al. Molecular Epidemiology and Risk Factors for Extended-Spectrum β-Lactamase-Producing Enterobacterales in Long-Term Care Residents. *J Am Med Dir Assoc*. 2022;**23**(3):475-81.e5. 10.1016/j.jamda.2021.06.030

251. Kremer A, Whitmer G, Diaz A, Sajwani A, Navarro A, Arshad M. ESBL Escherichia coli Isolates Have Enhanced Gut Colonization Capacity Compared to Non-ESBL Strains in Neonatal Mice. *Microbiol Spectr*. 2022;**10**(5):e0058222. 10.1128/spectrum.00582-22

252. Kumar CPG, Bhatnagar T, Sathya Narayanan G, Swathi SS, Sindhuja V, Siromany VA, et al. High-level Colonization With Antibiotic-Resistant Enterobacterales Among Individuals in a Semi-Urban Setting in South India: An Antibiotic Resistance in Communities and Hospitals (ARCH) Study. *Clin Infect Dis*. 2023;**77**(Suppl 1):S111-s7. 10.1093/cid/ciad220

253. Lecuru M, Daniau C, Alfandari S, Dumartin C, Bajolet O, Blanchard H, et al. Evolution of antibiotic treatments for healthcare-associated infections caused by extended-spectrum beta-lactamase-producing Enterobacteriaceae in France. *Infect Dis Now*. 2022;**52**(7):396-402. 10.1016/j.idnow.2022.08.003

254. Lee CM, Lee S, Kim ES, Kim HB, Park WB, Moon SM, et al. Disease burden of bacteraemia with extended-spectrum beta-lactamase-producing and carbapenem-resistant Enterobacterales in Korea. *J Hosp Infect*. 2024;**144**:85-93. 10.1016/j.jhin.2023.11.013

255. Lewis JM, Mphasa M, Banda R, Beale MA, Heinz E, Mallewa J, et al. Colonization dynamics of extended-spectrum beta-lactamase-producing Enterobacterales in the gut of Malawian adults. *Nat Microbiol*. 2022;**7**(10):1593-604. 10.1038/s41564-022-01216-7

256. Li Z, Li J, Liu J, Peng Y, Li Z, Wang M, et al. High Carriage of Extended-Spectrum, Beta Lactamase-Producing, and Colistin-Resistant Enterobacteriaceae in Tibetan Outpatients with Diarrhea. *Antibiotics (Basel)*. 2022;**11**(4). 10.3390/antibiotics11040508

257. Lindblom A, Kiszakiewicz C, Kristiansson E, Yazdanshenas S, Kamenska N, Karami N, et al. The impact of the ST131 clone on recurrent ESBL-producing E. coli urinary tract infection: a prospective comparative study. *Sci Rep*. 2022;**12**(1):10048. 10.1038/s41598-022-14177-y

258. Ling W, Cadavid-Restrepo A, Furuya-Kanamori L, Harris PNA, Paterson DL. Incidence and predictors of Escherichia coli producing extended-spectrum beta-lactamase (ESBL-Ec) in Queensland, Australia from 2010 to 2019: a population-based spatial analysis. *Epidemiol Infect*. 2022;**150**:e178. 10.1017/s0950268822001637

259. Ling W, Paterson DL, Harris PNA, Furuya-Kanamori L, Edwards F, Laupland KB. Mortality, hospital length of stay, and recurrent bloodstream infections associated with extended-spectrum beta-lactamase-producing Escherichia coli in a low prevalence region: A 20-year population-based large cohort study. *Int J Infect Dis*. 2024;**138**:84-90. 10.1016/j.ijid.2023.11.007

260. Literacka E, Konior M, Izdebski R, Żabicka D, Herda M, Gniadkowski M, et al. High risk of intestinal colonization with ESBL-producing Escherichia coli among soldiers of military contingents in specific geographic regions. *Eur J Clin Microbiol Infect Dis*. 2023;**42**(12):1523-30. 10.1007/s10096-023-04684-9

261. Ljubović AD, Granov Ð, Husić E, Gačanović D, Halković J, Lab Ing A, et al. Prevalence of extended-spectrum β-lactamase and carbapenem-resistant Klebsiella pneumoniae in clinical samples. *Saudi Med J*. 2023;**44**(8):801-7. 10.15537/smj.2023.44.8.20230237

262. Lombardi G, Tanzarella ES, Cutuli SL, De Pascale G. Treatment of severe infections caused by ESBL or carbapenemases-producing Enterobacteriaceae. *Med Intensiva (Engl Ed)*. 2023;**47**(1):34-44. 10.1016/j.medine.2022.09.002

263. Mahmud B, Wallace MA, Reske KA, Alvarado K, Muenks CE, Rasmussen DA, et al. Epidemiology of Plasmid Lineages Mediating the Spread of Extended-Spectrum Beta-Lactamases among Clinical Escherichia coli. *mSystems*. 2022;**7**(5):e0051922. 10.1128/msystems.00519-22

264. Mannathoko N, Lautenbach E, Mosepele M, Otukile D, Sewawa K, Glaser L, et al. Performance of CHROMagar ESBL media for the surveillance of extended-spectrum cephalosporin-resistant Enterobacterales (ESCrE) from rectal swabs in Botswana. *J Med Microbiol*. 2023;**72**(11). 10.1099/jmm.0.001770

265. Matsumoto H, Komiya K, Ichihara S, Nagaoka Y, Yamanaka M, Nishiyama Y, et al. Factors Associated with Extended-spectrum β-lactamase-producing Enterobacteria Isolated from Respiratory Samples. *Intern Med*. 2023;**62**(14):2043-50. 10.2169/internalmedicine.0690-22

266. Matta-Chuquisapon J, Valencia-Bazalar E, Sevilla-Andrade C, Barrón-Pastor HJ. Phylogeny and antimicrobial resistance of extended-spectrum beta-lactamaseproducing Escherichia coli from hospitalized oncology patients in Perú. *Biomedica*. 2022;**42**(3):470-8. 10.7705/biomedica.6263

267. Mbonyingingo D, Nzoyikorera N, Diawara I, Fdany K, Katfy K, Maaloum F, et al. Faecal carriage of extended-spectrum beta-lactamase-producing Enterobacterales in a paediatric intensive care unit in Casablanca, Morocco. *J Hosp Infect*. 2023;**133**:109-10. 10.1016/j.jhin.2022.09.028

268. Medugu N, Tickler IA, Duru C, Egah R, James AO, Odili V, et al. Phenotypic and molecular characterization of beta-lactam resistant Multidrug-resistant Enterobacterales isolated from patients attending six hospitals in Northern Nigeria. *Sci Rep*. 2023;**13**(1):10306. 10.1038/s41598-023-37621-z

269. Menezes J, Frosini SM, Belas A, Marques C, da Silva JM, Amaral AJ, et al. Longitudinal study of ESBL/AmpC-producing Enterobacterales strains sharing between cohabiting healthy companion animals and humans in Portugal and in the United Kingdom. *Eur J Clin Microbiol Infect Dis*. 2023;**42**(8):1011-24. 10.1007/s10096-023-04629-2

270. Mirzaei B, Ebrahimi A, Keshavarzi S, Hydarzadeh S, Badmasti F, Dadar M, et al. Antibiotic Susceptibility, Biofilm-Forming Ability, and Prevalence of Extended-Spectrum Beta-Lactamase (ESBL)- and Biofilm-Associated Genes Among Klebsiella pneumoniae Isolates from Hospitalized Patients in Northwest of Iran. *Curr Microbiol*. 2023;**80**(5):175. 10.1007/s00284-023-03247-7

271. Moglad E, Altayb HN. Genomic characterization of extended spectrum beta lactamases producing multidrug-resistant Escherichia coli clinically isolated harboring chromosomally mediated CTX-M-15 from Alkharj, KSA. *Infect Genet Evol*. 2023;**116**:105526. 10.1016/j.meegid.2023.105526

272. Moreno-Mingorance A, Mir-Cros A, Goterris L, Rodriguez-Garrido V, Sulleiro E, Barberà MJ, et al. Increasing trend of antimicrobial resistance in Shigella associated with MSM transmission in Barcelona, 2020-21: outbreak of XRD Shigella sonnei and dissemination of ESBL-producing Shigella flexneri. *J Antimicrob Chemother*. 2023;**78**(4):975-82. 10.1093/jac/dkad031

273. Morin-Le Bihan A, Le Neindre K, Dejoies L, Piau C, Donnio PY, Ménard G. Use of the quantitative antibiogram method for assessing nosocomial transmission of ESBL-producing Enterobacterales in a French hospital. *J Hosp Infect*. 2023;**135**:132-8. 10.1016/j.jhin.2023.01.023

274. Moussa B, Oumokhtar B, Arhoune B, Massik A, Elfakir S, Khalis M, et al. Gut acquisition of Extended-spectrum β-lactamases-producing Klebsiella pneumoniae in preterm neonates: Critical role of enteral feeding, and endotracheal tubes in the neonatal intensive care unit (NICU). *PLoS One*. 2023;**18**(11):e0293949. 10.1371/journal.pone.0293949

275. Mukheef SA, Eleroui M, Al-Dahmoshi HOM, Ben Amara I. Prevalence of Some Group A Beta-Lactamase Genes among Uropathogenic Escherichia coli isolated from Women with Cystitis. *Cell Mol Biol (Noisy-le-grand)*. 2022;**68**(10):84-9. 10.14715/cmb/2022.68.10.13

276. Mutua JM, Njeru JM, Musyoki AM. Extended-spectrum β-lactamase- producing gram-negative bacterial infections in severely ill COVID-19 patients admitted in a national referral hospital, Kenya. *Ann Clin Microbiol Antimicrob*. 2023;**22**(1):91. 10.1186/s12941-023-00641-8

277. Nakai M, Oka K, Watanabe G, Kamei K, Tsukada N, Mori R, et al. Epidemiology and molecular characterization of fecal carriage of third-generation cephalosporin-resistant Enterobacterales among elderly residents in Japan. *J Infect Chemother*. 2022;**28**(4):569-75. 10.1016/j.jiac.2021.12.033

278. Namikawa H, Imoto W, Yamada K, Tochino Y, Kaneko Y, Kakeya H, et al. Predictors of mortality from extended-spectrum beta-lactamase-producing Enterobacteriaceae bacteremia. *Emerg Microbes Infect*. 2023;**12**(1):2217951. 10.1080/22221751.2023.2217951

279. Neffe L, Forde TL, Oravcova K, Köhler U, Bautsch W, Tomasch J, et al. Genomic epidemiology of clinical ESBL-producing Enterobacteriaceae in a German hospital suggests infections are primarily community- and regionally-acquired. *Microb Genom*. 2022;**8**(12). 10.1099/mgen.0.000901

280. Nejad MK, Hasani A, Soofiyani SR, Nahandi MZ, Hasani A. Aptitude of Uropathogenic Escherichia coli in Renal Transplant Recipients: A Comprehensive Review on Characteristic Features, and Production of Extended Spectrum β-Lactamase. *Curr Microbiol*. 2023;**80**(12):382. 10.1007/s00284-023-03476-w

281. Nkengkana OA, Founou RC, Founou LL, Dimani BD, Koudoum PL, Zemtsa JR, et al. Phenotypic and genotypic characterization of multidrug resistant and extended-spectrum β-lactamase-producing Enterobacterales isolated from clinical samples in the western region in Cameroon. *BMC Infect Dis*. 2023;**23**(1):819. 10.1186/s12879-023-08742-7

282. Noguchi T, Shinohara K, Tsuchido Y, Yukawa S, Yamamoto M, Matsumura Y, et al. Oral Antibiotic Transition in Patients with Bacteremia with a Urinary Source Due to Extended-Spectrum β-Lactamase-Producing Escherichia coli. *Jpn J Infect Dis*. 2022;**75**(2):205-8. 10.7883/yoken.JJID.2020.1084

283. Obadare TO, Adeyemo AT, Odetoyin BW, Ugowe OJ, Anyabolu CH, Adejuyigbe EA, et al. Rectal carriage of extended-spectrum β-lactamase-producing Enterobacteriales among neonates admitted into a special care baby unit, southwest Nigeria. *Trans R Soc Trop Med Hyg*. 2023;**117**(7):528-35. 10.1093/trstmh/trad010

284. Obeng-Nkrumah N, Hansen DS, Awuah-Mensah G, Blankson NK, Frimodt-Møller N, Newman MJ, et al. High level of colonization with third-generation cephalosporin-resistant Enterobacterales in African community settings, Ghana. *Diagn Microbiol Infect Dis*. 2023;**106**(1):115918. 10.1016/j.diagmicrobio.2023.115918

285. Ohnishi T, Mishima Y, Naito T, Matsuda N, Ariji S, Umino D, et al. Clinical features and treatment strategies of febrile urinary tract infection caused by extended-spectrum beta-lactamase-producing Enterobacteriaceae in children: a multicenter retrospective observational study in Japan. *Int J Infect Dis*. 2022;**125**:97-102. 10.1016/j.ijid.2022.09.033

286. Oka K, Tetsuka N, Morioka H, Iguchi M, Kawamura K, Hayashi K, et al. Genetic and epidemiological analysis of ESBL-producing Klebsiella pneumoniae in three Japanese university hospitals. *J Infect Chemother*. 2022;**28**(9):1286-94. 10.1016/j.jiac.2022.05.013

287. Oktaviani Sulikah SR, Hasanah M, Setyarini W, Parathon H, Kitagawa K, Nakanishi N, et al. Occurrence of Carriage of Multidrug Resistant Enterobacteriaceae among Pregnant Women in the Primary Health Center and Hospital Setting in Surabaya, Indonesia. *Microb Drug Resist*. 2022;**28**(1):48-55. 10.1089/mdr.2020.0506

288. Onishi R, Shigemura K, Osawa K, Yang YM, Maeda K, Tanimoto H, et al. Impact on quinolone resistance of plasmid-mediated quinolone resistance gene and mutations in quinolone resistance-determining regions in extended spectrum beta lactamase-producing Klebsiella pneumoniae isolated from urinary tract infection patients. *Pathog Dis*. 2022;**80**(1). 10.1093/femspd/ftac030

289. Opstrup KV, Christiansen G, Birkelund S. Beta-lactam induced morphological changes in serum of extended-spectrum beta-lactamase-producing Klebsiella pneumoniae blood isolates. *Microbes Infect*. 2023;**25**(1-2):105036. 10.1016/j.micinf.2022.105036

290. Osman M, Yassine I, Hamze M, Al Mir H, Ghorbani Tajani A, Bisha B, et al. Emergence of Extended-Spectrum Cephalosporin- and Colistin-Resistant Enterobacterales in Otherwise Healthy University Students. *Microb Drug Resist*. 2024;**30**(2):101-7. 10.1089/mdr.2023.0213

291. Ota Y, Furuhashi K, Hirai N, Nagao Y, Ishikawa J, Nagura O, et al. Utility of the MBT STAR-Cepha kit in detecting extended-spectrum β-lactamase producers in clinical urine samples and positive blood cultures using matrix-assisted laser desorption/ionization time-of-flight mass spectrometry. *J Microbiol Methods*. 2023;**211**:106756. 10.1016/j.mimet.2023.106756

292. Park KS, Kim DR, Baek JY, Shin A, Kim KR, Park H, et al. Susceptibility to Fosfomycin and Nitrofurantoin of ESBL-Positive Escherichia coli and Klebsiella pneumoniae Isolated From Urine of Pediatric Patients. *J Korean Med Sci*. 2023;**38**(48):e361. 10.3346/jkms.2023.38.e361

293. Paulitsch-Fuchs AH, Melchior N, Haitzmann T, Fingerhut T, Feierl G, Baumert R, et al. Analysis of Extended Spectrum Beta Lactamase (ESBL) Genes of Non-Invasive ESBL Enterobacterales in Southeast Austria in 2017. *Antibiotics (Basel)*. 2022;**12**(1). 10.3390/antibiotics12010001

294. Paumier A, Asquier-Khati A, Thibaut S, Coeffic T, Lemenand O, Larramendy S, et al. Assessment of Factors Associated With Community-Acquired Extended-Spectrum β-Lactamase-Producing Escherichia coli Urinary Tract Infections in France. *JAMA Netw Open*. 2022;**5**(9):e2232679. 10.1001/jamanetworkopen.2022.32679

295. Peng Y, Sin DZY, Tun HM. International travel, the gut microbiome, and ESBL-E coli carriage. *Lancet Microbe*. 2022;**3**(10):e730. 10.1016/s2666-5247(22)00201-4

296. Pérez-Granados EE, Díaz-Chávez E, Álvarez JA, Macías AE, Arreguín V, Gutiérrez-Canales LG, et al. Impact of infections and extended-spectrum beta-lactamase (ESBL)-producing Enterobacteriaceae on graft and patient survival in a kidney transplantation program in Mexico. *Gac Med Mex*. 2022;**158**(5):295-301. 10.24875/gmm.M22000698

297. Pérez-Nadales E, Fernández-Ruiz M, Gutiérrez-Gutiérrez B, Pascual Á, Rodríguez-Baño J, Martínez-Martínez L, et al. Extended-spectrum β-lactamase-producing and carbapenem-resistant Enterobacterales bloodstream infection after solid organ transplantation: Recent trends in epidemiology and therapeutic approaches. *Transpl Infect Dis*. 2022;**24**(4):e13881. 10.1111/tid.13881

298. Perez-Palacios P, Girlich D, Soraa N, Lamrani A, Maoulainine FMR, Bennaoui F, et al. Multidrug-resistant Enterobacterales responsible for septicaemia in a neonatal intensive care unit in Morocco. *J Glob Antimicrob Resist*. 2023;**33**:208-17. 10.1016/j.jgar.2023.02.011

299. Pilmis B, Weiss E, Scemla A, Le Monnier A, Grossi PA, Slavin MA, et al. Multidrug-resistant Enterobacterales infections in abdominal solid organ transplantation. *Clin Microbiol Infect*. 2023;**29**(1):38-43. 10.1016/j.cmi.2022.06.005

300. Promsuwan O, Malathum K, Ingsathit A. Epidemiology of extended-spectrum β-lactamase-producing Enterobacterales infection in kidney transplant recipients. *Antimicrob Resist Infect Control*. 2023;**12**(1):123. 10.1186/s13756-023-01308-x

301. Pustam A, Jayaraman J, Ramsubhag A. Characterization of Beta-Lactam Resistance Genes and Virulence Factors Associated with Multidrug-Resistant Klebsiella pneumoniae Isolated from Patients at Major Hospitals in Trinidad, West Indies. *Curr Microbiol*. 2022;**79**(9):278. 10.1007/s00284-022-02972-9

302. Qasemi A, Rahimi F, Katouli M. Clonal groups of extended-spectrum β-lactamase and biofilm producing uropathogenic Escherichia coli in Iran. *Pathog Glob Health*. 2022;**116**(8):485-97. 10.1080/20477724.2021.2011578

303. Raffelsberger N, Buczek DJ, Svendsen K, Småbrekke L, Pöntinen AK, Löhr IH, et al. Community carriage of ESBL-producing Escherichia coli and Klebsiella pneumoniae: a cross-sectional study of risk factors and comparative genomics of carriage and clinical isolates. *mSphere*. 2023;**8**(4):e0002523. 10.1128/msphere.00025-23

304. Raouf FEA, Benyagoub E, Alkhudhairy MK, Akrami S, Saki M. Extended-spectrum beta-lactamases among Klebsiella pneumoniae from Iraqi patients with community-acquired pneumonia. *Rev Assoc Med Bras (1992)*. 2022;**68**(6):833-7. 10.1590/1806-9282.20220222

305. Raphael E, Inamdar PP, Belmont C, Shariff-Marco S, Huang AJ, Chambers HF. Spatial clusters of extended-spectrum beta-lactamase-producing Escherichia coli causing community-onset bacteriuria due to repeat infections: cluster analysis from a large urban medical center, San Francisco, 2014-2020. *Antimicrob Resist Infect Control*. 2023;**12**(1):115. 10.1186/s13756-023-01320-1

306. Ríos E, Del Carmen López Diaz M, Culebras E, Rodríguez-Avial I, Rodríguez-Avial C. Resistance to fosfomycin is increasing and is significantly associated with extended-spectrum β-lactamase-production in urinary isolates of Escherichia coli. *Med Microbiol Immunol*. 2022;**211**(5-6):269-72. 10.1007/s00430-022-00749-2

307. Rizk MA, Zaki MES, Mohamed HAA, Abdel-Hady DM, Montasser K. Extended-Spectrum β-Lactamase-Producing Escherichia coli and Virulence Genes in Pediatric Patients with Health-Care Urinary Tract Infections. *Infect Disord Drug Targets*. 2023;**23**(3):e041122210656. 10.2174/1871526523666221104150123

308. Rodríguez EC, Saavedra SY, Montaño LA, Sossa DP, Correa FP, Vaca JA, et al. Characterization of extended spectrum β-lactamases in Colombian clinical isolates of non-typhoidal Salmonella enterica between 1997 and 2022. *Biomedica*. 2023;**43**(3):374-84. 10.7705/biomedica.6891

309. Rodríguez-Molina D, Berglund F, Blaak H, Flach CF, Kemper M, Marutescu L, et al. International Travel as a Risk Factor for Carriage of Extended-Spectrum β-Lactamase-Producing Escherichia coli in a Large Sample of European Individuals-The AWARE Study. *Int J Environ Res Public Health*. 2022;**19**(8). 10.3390/ijerph19084758

310. Sallem N, Hammami A, Mnif B. Trends in human intestinal carriage of ESBL- and carbapenemase-producing Enterobacterales among food handlers in Tunisia: emergence of C1-M27-ST131 subclades, blaOXA-48 and blaNDM. *J Antimicrob Chemother*. 2022;**77**(8):2142-52. 10.1093/jac/dkac167

311. Sammarro M, Rowlingson B, Cocker D, Chidziwisano K, Jacob ST, Kajumbula H, et al. Risk Factors, Temporal Dependence, and Seasonality of Human Extended-Spectrum β-Lactamases-Producing Escherichia coli and Klebsiella pneumoniae Colonization in Malawi: A Longitudinal Model-Based Approach. *Clin Infect Dis*. 2023;**77**(1):1-8. 10.1093/cid/ciad117

312. Sampah J, Owusu-Frimpong I, Aboagye FT, Owusu-Ofori A. Prevalence of carbapenem-resistant and extended-spectrum beta-lactamase-producing Enterobacteriaceae in a teaching hospital in Ghana. *PLoS One*. 2023;**18**(10):e0274156. 10.1371/journal.pone.0274156

313. Santona A, Sumbana JJ, Fiamma M, Deligios M, Taviani E, Simbine SE, et al. High-risk lineages among extended-spectrum β-lactamase-producing Escherichia coli from extraintestinal infections in Maputo Central Hospital, Mozambique. *Int J Antimicrob Agents*. 2022;**60**(4):106649. 10.1016/j.ijantimicag.2022.106649

314. Sgayer I, Glikman D, Shqara RA, Maimon M, Rechnitzer H, Lowenstein L, et al. Maternal colonization with extended-spectrum β-lactamase-producing Enterobacteriaceae in term versus preterm pregnancies. *Int J Gynaecol Obstet*. 2023;**161**(2):447-54. 10.1002/ijgo.14555

315. Shafiq M, Bilal H, Permana B, Xu D, Cai G, Li X, et al. Characterization of antibiotic resistance genes and mobile elements in extended-spectrum β-lactamase-producing Escherichia coli strains isolated from hospitalized patients in Guangdong, China. *J Appl Microbiol*. 2023;**134**(7). 10.1093/jambio/lxad125

316. Shahkolahi S, Shakibnia P, Shahbazi S, Sabzi S, Badmasti F, Asadi Karam MR, et al. Detection of ESBL and AmpC producing Klebsiella pneumoniae ST11 and ST147 from urinary tract infections in Iran. *Acta Microbiol Immunol Hung*. 2022;**69**(4):303-13. 10.1556/030.2022.01808

317. Shang C, Yang R, Yang Y, Zhang H, Zhang J, Xia Q, et al. Colonization of extended-spectrum β-lactamase-producing Enterobacteriaceae does not affect subsequent infection and liver transplant outcomes: a retrospective observational cohort study. *Front Public Health*. 2023;**11**:1207889. 10.3389/fpubh.2023.1207889

318. Sharma B, Yadav S, Khanal A, Shakya B, Shrestha K, Aryal S, et al. Fecal Carriage of Extended-Spectrum beta-Lactamase Producing Escherichia coli among Health Science Students. *J Nepal Health Res Counc*. 2022;**20**(1):160-5. 10.33314/jnhrc.v20i01.3872

319. Sharma BK, Sharma BP, Kunwar A, Basnet N, Magar PD, Adhikari SJJoGE, et al. Prevalence of Extended Spectrum β-Lactamase Producers (ESBLs) with antibiotic resistance pattern of Gram negative pathogenic bacteria isolated from door handles in hospitals of Pokhara, Western Nepal. 2023;**21**(1):139.

320. Shimizu T, Kido N, Miyashita N, Tanaka S, Omiya T, Morikaku K, et al. Antimicrobial resistance in Escherichia coli isolates from Japanese raccoon dogs (Nyctereutes viverrinus) in Kanagawa Prefecture, Japan: Emergence of extended-spectrum cephalosporin-resistant human-related clones. *J Med Microbiol*. 2022;**71**(12). 10.1099/jmm.0.001631

321. Shropshire WC, Strope B, Selvaraj Anand S, Bremer J, McDaneld P, Bhatti MM, et al. Temporal dynamics of genetically heterogeneous extended-spectrum cephalosporin-resistant Escherichia coli bloodstream infections. *mSphere*. 2023;**8**(4):e0018323. 10.1128/msphere.00183-23

322. Siriphap A, Kitti T, Khuekankaew A, Boonlao C, Thephinlap C, Thepmalee C, et al. High prevalence of extended-spectrum beta-lactamase-producing Escherichia coli and Klebsiella pneumoniae isolates: A 5-year retrospective study at a Tertiary Hospital in Northern Thailand. *Front Cell Infect Microbiol*. 2022;**12**:955774. 10.3389/fcimb.2022.955774

323. Soyaltın E, Erfidan G, Kavruk M, Çamlar SA, Yılmaz N, Alaygut D, et al. Predictors of febrile urinary tract infection caused by extended-spectrum beta-lactamase-producing bacteria. *Turk J Pediatr*. 2022;**64**(2):265-73. 10.24953/turkjped.2020.2371

324. Sserwadda I, Kidenya BR, Kanyerezi S, Akaro IL, Mkinze B, Mshana SE, et al. Unraveling virulence determinants in extended-spectrum beta-lactamase-producing Escherichia coli from East Africa using whole-genome sequencing. *BMC Infect Dis*. 2023;**23**(1):587. 10.1186/s12879-023-08579-0

325. Standing JF. Comment on: Flomoxef for neonates: extending options for treatment of neonatal sepsis caused by ESBL-producing Enterobacterales. *J Antimicrob Chemother*. 2022;**77**(7):2046-7. 10.1093/jac/dkac043

326. Sultana KF, Akter A, Saha SR, Ahmed F, Alam S, Jafar T, et al. Bacterial profile, antimicrobial resistance, and molecular detection of ESBL and quinolone resistance gene of uropathogens causing urinary tract infection in the southeastern part of Bangladesh. *Braz J Microbiol*. 2023;**54**(2):803-15. 10.1007/s42770-023-00942-2

327. Symanzik C, Hillenbrand J, Stasielowicz L, Greie JC, Friedrich AW, Pulz M, et al. Novel insights into pivotal risk factors for rectal carriage of extended-spectrum-β-lactamase-producing enterobacterales within the general population in Lower Saxony, Germany. *J Appl Microbiol*. 2022;**132**(4):3256-64. 10.1111/jam.15399

328. Tacconelli E, Górska A, De Angelis G, Lammens C, Restuccia G, Schrenzel J, et al. Estimating the association between antibiotic exposure and colonization with extended-spectrum β-lactamase-producing Gram-negative bacteria using machine learning methods: a multicentre, prospective cohort study. 2020;**26**(1):87-94.

329. Tadesse S, Mulu W, Genet C, Kibret M, Belete MA. Emergence of High Prevalence of Extended-Spectrum Beta-Lactamase and Carbapenemase-Producing Enterobacteriaceae Species among Patients in Northwestern Ethiopia Region. *Biomed Res Int*. 2022;**2022**:5727638. 10.1155/2022/5727638

330. Tamma PD, Aitken SL, Bonomo RA, Mathers AJ, van Duin D, Clancy CJ. Infectious Diseases Society of America 2022 Guidance on the Treatment of Extended-Spectrum β-lactamase Producing Enterobacterales (ESBL-E), Carbapenem-Resistant Enterobacterales (CRE), and Pseudomonas aeruginosa with Difficult-to-Treat Resistance (DTR-P. aeruginosa). *Clin Infect Dis*. 2022;**75**(2):187-212. 10.1093/cid/ciac268

331. Tanimoto H, Shigemura K, Osawa K, Kado M, Onishi R, Fang SB, et al. Comparative genetic analysis of the antimicrobial susceptibilities and virulence of hypermucoviscous and non-hypermucoviscous ESBL-producing Klebsiella pneumoniae in Japan. *J Microbiol Immunol Infect*. 2023;**56**(1):93-103. 10.1016/j.jmii.2022.08.010

332. Toombs-Ruane LJ, Marshall JC, Benschop J, Drinković D, Midwinter AC, Biggs PJ, et al. Extended-spectrum β-lactamase- and AmpC β-lactamase-producing Enterobacterales associated with urinary tract infections in the New Zealand community: a case-control study. *Int J Infect Dis*. 2023;**128**:325-34. 10.1016/j.ijid.2022.12.013

333. Tornberg-Belanger SN, Rwigi D, Mugo M, Kitheka L, Onamu N, Ounga D, et al. Antimicrobial resistance including Extended Spectrum Beta Lactamases (ESBL) among E. coli isolated from kenyan children at hospital discharge. *PLoS Negl Trop Dis*. 2022;**16**(3):e0010283. 10.1371/journal.pntd.0010283

334. Ullah N, Assawakongkarat T, Akeda Y, Chaichanawongsaroj N. Detection of Extended-spectrum β-lactamase-producing Escherichia coli isolates by isothermal amplification and association of their virulence genes and phylogroups with extraintestinal infection. *Sci Rep*. 2023;**13**(1):12022. 10.1038/s41598-023-39228-w

335. Umar UY, Giwa FJ, Ibrahim A, Gachi FS. Antimicrobial susceptibility pattern of extended-spectrum beta-lactamase-producing uropathogens in aminu Kano teaching hospital, Northwestern Nigeria. *Ann Afr Med*. 2023;**22**(4):508-14. 10.4103/aam.aam_155_22

336. van Kleef-van Koeveringe S, Matheeussen V, Jansens H, Perales Selva N, De Coninck D, De Bruyne K, et al. Epidemiology and molecular typing of multidrug-resistant bacteria in day care centres in Flanders, Belgium. *Epidemiol Infect*. 2023;**151**:e156. 10.1017/s0950268823001528

337. Wang G, Zhu Y, Feng S, Wei B, Zhang Y, Wang J, et al. Extended-spectrum beta-lactamase-producing Enterobacteriaceae related urinary tract infection in adult cancer patients: a multicenter retrospective study, 2015-2019. *BMC Infect Dis*. 2023;**23**(1):129. 10.1186/s12879-023-08023-3

338. Watanabe N, Watari T, Otsuka Y, Yamagata K, Fujioka M. Clinical characteristics and antimicrobial susceptibility of Klebsiella pneumoniae, Klebsiella variicola and Klebsiella quasipneumoniae isolated from human urine in Japan. *J Med Microbiol*. 2022;**71**(6). 10.1099/jmm.0.001546

339. Wilkowski P, Hryniewiecka E, Jasińska K, Pączek L, Ciszek M. Breaking Antimicrobial Resistance: High-Dose Amoxicillin with Clavulanic Acid for Urinary Tract Infections Due to Extended-Spectrum Beta-Lactamase (ESBL)-Producing Klebsiella pneumoniae. *Ann Transplant*. 2023;**28**:e939258. 10.12659/aot.939258

340. Yap PSX, Chong CW, Ponnampalavanar S, Ramli R, Harun A, Tengku Jamaluddin TZM, et al. A multicentre study to determine the in vitro efficacy of flomoxef against extended-spectrum beta-lactamase producing Escherichia coli in Malaysia. *PeerJ*. 2023;**11**:e16393. 10.7717/peerj.16393

341. Zamudio R, Boerlin P, Beyrouthy R, Madec JY, Schwarz S, Mulvey MR, et al. Dynamics of extended-spectrum cephalosporin resistance genes in Escherichia coli from Europe and North America. *Nat Commun*. 2022;**13**(1):7490. 10.1038/s41467-022-34970-7

342. Zhang T, Huang X, Xu T, Li S, Cui M. Pyogenic liver abscess caused by extended-spectrum β-lactamase-producing hypervirulent Klebsiella pneumoniae diagnosed by third-generation sequencing: a case report and literature review. *J Int Med Res*. 2023;**51**(10):3000605231206296. 10.1177/03000605231206296

343. Zurita J, Sevillano G, Paz YMA, Haro N, Larrea-Álvarez M, Alcocer I, et al. Dominance of ST131, B2, blaCTX-M-15, and papA-papC-kpsMII-uitA among ESBL Escherichia coli isolated from bloodstream infections in Quito, Ecuador: a 10-year surveillance study (2009-2019). *J Appl Microbiol*. 2023;**134**(11). 10.1093/jambio/lxad269

344. Atta HI, Idris SM, Gulumbe BH, Awoniyi OJ. Detection of extended spectrum beta-lactamase genes in strains of Escherichia coli and Klebsiella pneumoniae isolated from recreational water and tertiary hospital waste water in Zaria, Nigeria. *Int J Environ Health Res*. 2022;**32**(9):2074-82. 10.1080/09603123.2021.1940884

345. Aworh MK, Ekeng E, Nilsson P, Egyir B, Owusu-Nyantakyi C, Hendriksen RS. Extended-Spectrum ß-Lactamase-Producing Escherichia coli Among Humans, Beef Cattle, and Abattoir Environments in Nigeria. *Front Cell Infect Microbiol*. 2022;**12**:869314. 10.3389/fcimb.2022.869314

346. Azuma T, Uchiyama T, Zhang D, Usui M, Hayashi T. Distribution and characteristics of carbapenem-resistant and extended-spectrum β-lactamase (ESBL) producing Escherichia coli in hospital effluents, sewage treatment plants, and river water in an urban area of Japan. *Sci Total Environ*. 2022;**839**:156232. 10.1016/j.scitotenv.2022.156232

347. Bager SL, Kakaala I, Kudirkiene E, Byarugaba DK, Olsen JE. GENOMIC CHARACTERIZATION OF MULTIDRUG-RESISTANT EXTENDED-SPECTRUM β-LACTAMASE-PRODUCING ESCHERICHIA COLI AND KLEBSIELLA PNEUMONIAE FROM CHIMPANZEES (PAN TROGLODYTES) FROM WILD AND SANCTUARY LOCATIONS IN UGANDA. *J Wildl Dis*. 2022;**58**(2):269-78. 10.7589/jwd-d-21-00068

348. Banerjee A, Pal S, Goswami P, Batabyal K, Joardar SN, Dey S, et al. Docking analysis of circulating CTX-M variants in multi-drug resistant, beta-lactamase and biofilm-producing E. coli isolated from pet animals and backyard livestock. *Microb Pathog*. 2022;**170**:105700. 10.1016/j.micpath.2022.105700

349. Bastidas-Caldes C, Ochoa J, Guerrero-Latorre L, Moyota-Tello C, Tapia W, Rey-Pérez JM, et al. Removal of Extended-Spectrum Beta-Lactamase-Producing Escherichia coli, ST98, in Water for Human Consumption by Black Ceramic Water Filters in Low-Income Ecuadorian Highlands. *Int J Environ Res Public Health*. 2022;**19**(8). 10.3390/ijerph19084736

350. Cherak Z, Loucif L, Moussi A, Bendjama E, Benbouza A, Rolain JM. Emergence of Metallo-β-Lactamases and OXA-48 Carbapenemase Producing Gram-Negative Bacteria in Hospital Wastewater in Algeria: A Potential Dissemination Pathway Into the Environment. *Microb Drug Resist*. 2022;**28**(1):23-30. 10.1089/mdr.2020.0617

351. Cohen A, Poupko L, Craddock HA, Motro Y, Khalfin B, Zelinger A, et al. Fecal Microbiome Features Associated with Extended-Spectrum β-Lactamase-Producing Enterobacterales Carriage in Dairy Heifers. *Animals (Basel)*. 2022;**12**(14). 10.3390/ani12141738

352. Easler M, Cheney C, Johnson JD, Zadeh MK, Nguyen JN, Yiu SY, et al. Resistome characterization of extended-spectrum beta-lactamase (ESBL)-producing Escherichia coli isolated from wastewater treatment utilities in Oregon. *J Water Health*. 2022;**20**(4):670-9. 10.2166/wh.2022.292

353. Ewbank AC, Fuentes-Castillo D, Sacristán C, Cardoso B, Esposito F, Fuga B, et al. Extended-spectrum β-lactamase (ESBL)-producing Escherichia coli survey in wild seabirds at a pristine atoll in the southern Atlantic Ocean, Brazil: First report of the O25b-ST131 clone harboring bla(CTX-M-8). *Sci Total Environ*. 2022;**806**(Pt 2):150539. 10.1016/j.scitotenv.2021.150539

354. Fetahagić M, Ibrahimagić A, Uzunović S, Beader N, Elveđi-Gašparović V, Luxner J, et al. Detection and characterisation of extended-spectrum and plasmid-mediated AmpC β-lactamase produced by Escherichia coli isolates found at poultry farms in Bosnia and Herzegovina. *Arh Hig Rada Toksikol*. 2021;**72**(4):305-14. 10.2478/aiht-2021-72-3560

355. Garcia-Fierro R, Drapeau A, Dazas M, Saras E, Rodrigues C, Brisse S, et al. Comparative phylogenomics of ESBL-, AmpC- and carbapenemase-producing Klebsiella pneumoniae originating from companion animals and humans. *J Antimicrob Chemother*. 2022;**77**(5):1263-71. 10.1093/jac/dkac041

356. Gelalcha BD, Ensermu DB, Agga GE, Vancuren M, Gillespie BE, D'Souza DH, et al. Prevalence of Antimicrobial Resistant and Extended-Spectrum Beta-Lactamase-producing Escherichia coli in Dairy Cattle Farms in East Tennessee. *Foodborne Pathog Dis*. 2022;**19**(6):408-16. 10.1089/fpd.2021.0101

357. Gelalcha BD, Kerro Dego O. Extended-Spectrum Beta-Lactamases Producing Enterobacteriaceae in the USA Dairy Cattle Farms and Implications for Public Health. *Antibiotics (Basel)*. 2022;**11**(10). 10.3390/antibiotics11101313

358. Grevskott DH, Ghavidel FZ, Svanevik CS, Marathe NP. Resistance profiles and diversity of β-lactamases in Escherichia coli strains isolated from city-scale sewage surveillance in Bergen, Norway mimic clinical prevalence. *Ecotoxicol Environ Saf*. 2021;**226**:112788. 10.1016/j.ecoenv.2021.112788

359. Gruel G, Couvin D, Guyomard-Rabenirina S, Arlet G, Bambou JC, Pot M, et al. High Prevalence of bla (CTXM-1)/IncI1-Iγ/ST3 Plasmids in Extended-Spectrum β-Lactamase-Producing Escherichia coli Isolates Collected From Domestic Animals in Guadeloupe (French West Indies). *Front Microbiol*. 2022;**13**:882422. 10.3389/fmicb.2022.882422

360. Haeili M, Salehzeinali H, Mirzaei S, Pishnian Z, Ahmadi A. Molecular characterization of quinolone resistance and antimicrobial resistance profiles of Klebsiella pneumoniae and Escherichia coli isolated from human and broiler chickens. *Int J Environ Health Res*. 2022;**32**(6):1382-92. 10.1080/09603123.2021.1885632

361. Hasan B, Swedberg G. Molecular Characterization of Clinically Relevant Extended-Spectrum β-Lactamases bla(CTX-M-15)-Producing Enterobacteriaceae Isolated from Free-Range Chicken from Households in Bangladesh. *Microb Drug Resist*. 2022;**28**(7):780-6. 10.1089/mdr.2021.0264

362. Islam MS, Sobur MA, Rahman S, Ballah FM, Ievy S, Siddique MP, et al. Detection of bla(TEM), bla(CTX-M), bla(CMY), and bla(SHV) Genes Among Extended-Spectrum Beta-Lactamase-Producing Escherichia coli Isolated from Migratory Birds Travelling to Bangladesh. *Microb Ecol*. 2022;**83**(4):942-50. 10.1007/s00248-021-01803-x

363. Khalifeh OM, Obaidat MM. Urinary tract virulence genes in extended-spectrum beta-lactamase E. coli from dairy cows, beef cattle, and small ruminants. *Acta Trop*. 2022;**234**:106611. 10.1016/j.actatropica.2022.106611

364. Levent G, Schlochtermeier A, Vinasco J, Jennings J, Richeson J, Ives SE, et al. Long-Term Effects of Single-Dose Cephalosporin or Macrolide Use on the Prevalence of AmpC and Extended-Spectrum β-Lactamase Producing Escherichia coli in the Feces of Beef Cattle. *Microorganisms*. 2022;**10**(10). 10.3390/microorganisms10102071

365. Liu FL, Kuan NL, Yeh KS. Presence of the Extended-Spectrum-β-Lactamase and Plasmid-Mediated AmpC-Encoding Genes in Escherichia coli from Companion Animals-A Study from a University-Based Veterinary Hospital in Taipei, Taiwan. *Antibiotics (Basel)*. 2021;**10**(12). 10.3390/antibiotics10121536

366. Martak D, Guther J, Verschuuren TD, Valot B, Conzelmann N, Bunk S, et al. Populations of extended-spectrum β-lactamase-producing Escherichia coli and Klebsiella pneumoniae are different in human-polluted environment and food items: a multicentre European study. *Clin Microbiol Infect*. 2022;**28**(3):447.e7-.e14. 10.1016/j.cmi.2021.07.022

367. Miltgen G, Martak D, Valot B, Kamus L, Garrigos T, Verchere G, et al. One Health compartmental analysis of ESBL-producing Escherichia coli on Reunion Island reveals partitioning between humans and livestock. *J Antimicrob Chemother*. 2022;**77**(5):1254-62. 10.1093/jac/dkac054

368. Mutuku C, Melegh S, Kovacs K, Urban P, Virág E, Heninger R, et al. Characterization of β-Lactamases and Multidrug Resistance Mechanisms in Enterobacterales from Hospital Effluents and Wastewater Treatment Plant. *Antibiotics (Basel)*. 2022;**11**(6). 10.3390/antibiotics11060776

369. Nakayama T, Le Thi H, Thanh PN, Minh DTN, Hoang ON, Hoai PH, et al. Abundance of colistin-resistant Escherichia coli harbouring mcr-1 and extended-spectrum β-lactamase-producing E. coli co-harbouring bla(CTX-M-55) or (-65) with bla(TEM) isolates from chicken meat in Vietnam. *Arch Microbiol*. 2022;**204**(2):137. 10.1007/s00203-021-02746-0

370. Ndukui JG, Gikunju JK, Aboge GO, Mwaniki JK, Maina JN, Mbaria JM. Molecular Characterization of ESBLs and QnrS Producers From Selected Enterobacteriaceae Strains Isolated From Commercial Poultry Production Systems in Kiambu County, Kenya. *Microbiol Insights*. 2022;**15**:11786361211063619. 10.1177/11786361211063619

371. Nossair MA, Abd El Baqy FA, Rizk MSY, Elaadli H, Mansour AM, Abd El-Aziz AH, et al. Prevalence and Molecular Characterization of Extended-Spectrum β-Lactamases and AmpC β-lactamase-Producing Enterobacteriaceae among Human, Cattle, and Poultry. *Pathogens*. 2022;**11**(8). 10.3390/pathogens11080852

372. Osińska M, Nowakiewicz A, Zięba P, Gnat S, Łagowski D, Trościańczyk A. A rich mosaic of resistance in extended-spectrum β-lactamase-producing Escherichia coli isolated from red foxes (Vulpes vulpes) in Poland as a potential effect of increasing synanthropization. *Sci Total Environ*. 2022;**818**:151834. 10.1016/j.scitotenv.2021.151834

373. Pires J, Huber L, Hickman RA, Dellicour S, Lunha K, Leangapichart T, et al. Genome-associations of extended-spectrum ß-lactamase producing (ESBL) or AmpC producing E. coli in small and medium pig farms from Khon Kaen province, Thailand. *BMC Microbiol*. 2022;**22**(1):253. 10.1186/s12866-022-02646-3

374. Pungpian C, Angkititrakul S, Chuanchuen R. Genomic characterization of antimicrobial resistance in mcr-carrying ESBL-producing Escherichia coli from pigs and humans. *Microbiology (Reading)*. 2022;**168**(6). 10.1099/mic.0.001204

375. Rybak B, Potrykus M, Plenis A, Wolska L. Raw Meat Contaminated with Cephalosporin-Resistant Enterobacterales as a Potential Source of Human Home Exposure to Multidrug-Resistant Bacteria. *Molecules*. 2022;**27**(13). 10.3390/molecules27134151

376. Samir A, Abdel-Moein KA, Zaher HM. The Public Health Burden of Virulent Extended-Spectrum β-Lactamase-Producing Klebsiella pneumoniae Strains Isolated from Diseased Horses. *Vector Borne Zoonotic Dis*. 2022;**22**(4):217-24. 10.1089/vbz.2022.0004

377. Savin M, Bierbaum G, Schmithausen RM, Heinemann C, Kreyenschmidt J, Schmoger S, et al. Slaughterhouse wastewater as a reservoir for extended-spectrum β-lactamase (ESBL)-producing, and colistin-resistant Klebsiella spp. and their impact in a "One Health" perspective. *Sci Total Environ*. 2022;**804**:150000. 10.1016/j.scitotenv.2021.150000

378. Sebre S, Erku Abegaz W, Seman A, Awoke T, Mihret W, Desalegn Z, et al. Molecular Characterization of Extended-Spectrum Beta-Lactamase-Producing Enterobacteriaceae Isolates Collected from Inanimate Hospital Environments in Addis Ababa, Ethiopia. *Adv Exp Med Biol*. 2022;**1369**:69-80. 10.1007/5584_2021_646

379. Selmi R, Tayh G, Srairi S, Mamlouk A, Ben Chehida F, Lahmar S, et al. Prevalence, risk factors and emergence of extended-spectrum β-lactamase producing-, carbapenem- and colistin-resistant Enterobacterales isolated from wild boar (Sus scrofa) in Tunisia. *Microb Pathog*. 2022;**163**:105385. 10.1016/j.micpath.2021.105385

380. Shah A, Alam S, Kabir M, Fazal S, Khurshid A, Iqbal A, et al. Migratory birds as the vehicle of transmission of multi drug resistant extended spectrum β lactamase producing Escherichia fergusonii, an emerging zoonotic pathogen. *Saudi J Biol Sci*. 2022;**29**(5):3167-76. 10.1016/j.sjbs.2022.01.057

381. Sola M, Mani Y, Saras E, Drapeau A, Grami R, Aouni M, et al. Prevalence and Characterization of Extended-Spectrum β-Lactamase- and Carbapenemase-Producing Enterobacterales from Tunisian Seafood. *Microorganisms*. 2022;**10**(7). 10.3390/microorganisms10071364

382. Tigabie M, Biset S, Belachew T, Amare A, Moges F. Multidrug-resistant and extended-spectrum beta-lactamase-producing Enterobacteriaceae isolated from chicken droppings in poultry farms at Gondar City, Northwest Ethiopia. *PLoS One*. 2023;**18**(6):e0287043. 10.1371/journal.pone.0287043

383. Veloo Y, Thahir SSA, Rajendiran S, Hock LK, Ahmad N, Muthu V, et al. Multidrug-Resistant Gram-Negative Bacteria and Extended-Spectrum β-Lactamase-Producing Klebsiella pneumoniae from the Poultry Farm Environment. *Microbiol Spectr*. 2022;**10**(3):e0269421. 10.1128/spectrum.02694-21

384. Worku W, Desta M, Menjetta T. High prevalence and antimicrobial susceptibility pattern of salmonella species and extended-spectrum β-lactamase producing Escherichia coli from raw cattle meat at butcher houses in Hawassa city, Sidama regional state, Ethiopia. *PLoS One*. 2022;**17**(1):e0262308. 10.1371/journal.pone.0262308

385. Yoon S, Lee YJ. Molecular characteristics of ESBL-producing Escherichia coli isolated from chickens with colibacillosis. *J Vet Sci*. 2022;**23**(3):e37. 10.4142/jvs.21105

386. Zhao X, Zhao H, Zhou Z, Miao Y, Li R, Yang B, et al. Characterization of Extended-Spectrum β-Lactamase-Producing Escherichia coli Isolates That Cause Diarrhea in Sheep in Northwest China. *Microbiol Spectr*. 2022;**10**(4):e0159522. 10.1128/spectrum.01595-22

387. Abayneh M, Worku TJDTI. Prevalence of multidrug-resistant and extended-spectrum beta-lactamase (ESBL)-producing gram-negative bacilli: a meta-analysis report in Ethiopia. 2020;**14**:16.

388. Abera D, Alemu A, Mihret A, Negash AA, Abegaz WE, Cadwell KJPo. Colonization with extended spectrum beta-lactamase and carbapenemases producing Enterobacteriaceae among hospitalized patients at the global level: A systematic review and meta-analysis. 2023;**18**(11):e0293528.

389. Flokas ME, Alevizakos M, Shehadeh F, Andreatos N, Mylonakis EJIjoaa. Extended-spectrum β-lactamase-producing Enterobacteriaceae colonisation in long-term care facilities: a systematic review and meta-analysis. 2017;**50**(5):649-56.

390. Khadka C, Shyaula M, Syangtan G, Bista S, Tuladhar R, Singh A, et al. Extended-spectrum β-lactamases producing Enterobacteriaceae (ESBL-PE) prevalence in Nepal: A systematic review and meta-analysis. 2023;**901**:166164.

391. Lewis JM, Lester R, Garner P, Feasey NAJWor. Gut mucosal colonisation with extended-spectrum beta-lactamase producing Enterobacteriaceae in sub-Saharan Africa: a systematic review and meta-analysis. 2020;**4**:160.

392. Musa BM, Imam H, Lendel A, Abdulkadir I, Gumi HS, Aliyu MH, et al. The burden of extended-spectrum β-lactamase-producing Enterobacteriaceae in Nigeria: a systematic review and meta-analysis. 2020;**114**(4):241-8.

393. Pires J, Bernasconi OJ, Hauser C, Tinguely R, Atkinson A, Perreten V, et al. Intestinal colonisation with extended-spectrum cephalosporin- and colistin-resistant Enterobacteriaceae in HIV-positive individuals in Switzerland: molecular features and risk factors. *Int J Antimicrob Agents*. 2017;**49**(4):519-21. 10.1016/j.ijantimicag.2017.02.004

394. Mabeku LBK, EKANGOUO AE, Mewa JEK. Trends in Carriage of Extended Spectrum β-Lactamase and Carbapenemase Producing Enterobacteria Among HIV Infected Patients and Their Antimicrobial Susceptibility Patterns in Western-Cameroon. 2020.

395. Kyerew F. Intestinal Carriage of Extended-Spectrum Betalactamase Producing Enterobacteria in HivAids Patient: University Of Ghana; 2017.

396. Padmavathy K, Krishnan P, Rajasekaran SJBID. Incidence of bla genes among uropathogenic Escherichia coli isolates from HIV and non-HIV patients in South India. 2012;**12**(Suppl 1):P21.

397. Angel Díaz M, Ramón Hernández J, Martínez-Martínez L, Rodríguez-Baño J, Pascual A. [Extended-spectrum beta-lactamase-producing Escherichia coli and Klebsiella pneumoniae in Spanish hospitals: 2nd multicenter study (GEIH-BLEE project, 2006)]. *Enferm Infecc Microbiol Clin*. 2009;**27**(9):503-10. 10.1016/j.eimc.2008.09.006

398. Chaiwarith R, Pasogpakdee P, Salee P, Kanjanaratanakorn K, Sirisanthana T, Supparatpinyo K. Risk factors for extended-spectrum beta-lactamase-producing Klebsiella pneumoniae and Escherichia coli acquisition in a tertiary care teaching hospital in Thailand. *J Hosp Infect*. 2009;**71**(3):285-6. 10.1016/j.jhin.2008.11.021

399. Cotton MF, Wasserman E, Smit J, Whitelaw A, Zar HJJBid. High incidence of antimicrobial resistant organisms including extended spectrum beta-lactamase producing Enterobacteriaceae and methicillin-resistant Staphylococcus aureus in nasopharyngeal and blood isolates of HIV-infected children from Cape Town, South Africa. 2008;**8**:1-10.

400. Fang H, Huang H, Shi Y, Hedin G, Nord CE, Ullberg M. Prevalence of qnr determinants among extended-spectrum beta-lactamase-positive Enterobacteriaceae clinical isolates in southern Stockholm, Sweden. *Int J Antimicrob Agents*. 2009;**34**(3):268-70. 10.1016/j.ijantimicag.2009.03.016

401. Friedmann R, Raveh D, Zartzer E, Rudensky B, Broide E, Attias D, et al. Prospective evaluation of colonization with extended-spectrum beta-lactamase (ESBL)-producing enterobacteriaceae among patients at hospital admission and of subsequent colonization with ESBL-producing enterobacteriaceae among patients during hospitalization. *Infect Control Hosp Epidemiol*. 2009;**30**(6):534-42. 10.1086/597505

402. Mendonça N, Ferreira E, Louro D, Caniça M. Molecular epidemiology and antimicrobial susceptibility of extended- and broad-spectrum beta-lactamase-producing Klebsiella pneumoniae isolated in Portugal. *Int J Antimicrob Agents*. 2009;**34**(1):29-37. 10.1016/j.ijantimicag.2008.11.014

403. Pitout JD, Gregson DB, Campbell L, Laupland KB. Molecular characteristics of extended-spectrum-beta-lactamase-producing Escherichia coli isolates causing bacteremia in the Calgary Health Region from 2000 to 2007: emergence of clone ST131 as a cause of community-acquired infections. *Antimicrob Agents Chemother*. 2009;**53**(7):2846-51. 10.1128/aac.00247-09

404. Reiter KC, Fuchs SC, Barcellos NT, Zavascki AP. Risk factors for community-acquired infections caused by extended-spectrum beta-lactamase-producing Escherichia coli. *Arch Intern Med*. 2009;**169**(8):811; author reply -2. 10.1001/archinternmed.2009.97

405. Vignesh R, Shankar EM, Murugavel KG, Kumarasamy N, Sekar R, Irene P, et al. Urinary infections due to multi-drug-resistant Escherichia coli among persons with HIV disease at a tertiary AIDS care centre in South India. 2008;**110**(1):c55-c7.

406. Abdul-Mutakabbir JC, Griffith NC, Shields RK, Tverdek FP, Escobar ZKJId, therapy. Contemporary perspective on the treatment of Acinetobacter baumannii infections: insights from the Society of Infectious Diseases Pharmacists. 2021:1-26.

407. Alnimr AM, Alamri AM. Antimicrobial activity of cephalosporin-beta-lactamase inhibitor combinations against drug-susceptible and drug-resistant Pseudomonas aeruginosa strains. *J Taibah Univ Med Sci*. 2020;**15**(3):203-10. 10.1016/j.jtumed.2020.04.004

408. Alnimr AM, Alamri AMJJoTUMS. Antimicrobial activity of cephalosporin–beta-lactamase inhibitor combinations against drug-susceptible and drug-resistant Pseudomonas aeruginosa strains. 2020;**15**(3):203-10.

409. Archary M, Adler H, La Russa P, Mahabeer P, Bobat RAJP, Health IC. Bacterial infections in HIV-infected children admitted with severe acute malnutrition in Durban, South Africa. 2017;**37**(1):6-13.

410. Barkowsky G, Abt C, Pöhner I, Bieda A, Hammerschmidt S, Jacob A, et al. Antimicrobial Activity of Peptide-Coupled Antisense Peptide Nucleic Acids in Streptococcus pneumoniae. *Microbiol Spectr*. 2022;**10**(6):e0049722. 10.1128/spectrum.00497-22

411. Basnet A, Chand AB, Pokhrel N, Acharya S, Gurung P, Khanal LK, et al. Antimicrobial Susceptibility Pattern in Opportunistic Pathogens Isolated from Immunocompromised Patients. *J Nepal Health Res Counc*. 2023;**20**(3):664-71. 10.33314/jnhrc.v20i3.4047

412. Bell SFE, Ware RS, Lewis DA, Lahra MM, Whiley DM. Antimicrobial susceptibility assays for Neisseria gonorrhoeae: a proof-of-principle population-based retrospective analysis. *Lancet Microbe*. 2023;**4**(7):e544-e51. 10.1016/s2666-5247(23)00071-x

413. Blanc P, Bonnet F, Leleux O, Perrier A, Bessede E, Pereyre S, et al. Severe Bacterial Non-AIDS Infections in Persons With Human Immunodeficiency Virus: The Epidemiology and Evolution of Antibiotic Resistance Over an 18-Year Period (2000–2017) in the ANRS CO3 AquiVih-Nouvelle-Aquitaine Cohort. 2023;**76**(10):1814-21.

414. Blouin K, Lefebvre B, Trudelle A, Defay F, Perrault Sullivan G, Ezin Aloffan LND, et al. Correlates of Neisseria gonorrhoeae antimicrobial resistance: cross-sectional results from an open cohort sentinel surveillance network in Québec, Canada, 2016-2019. *BMJ Open*. 2023;**13**(8):e073849. 10.1136/bmjopen-2023-073849

415. Černiauskienė K, Dambrauskienė A, Vitkauskienė AJM. Associations between β-lactamase types of Acinetobacter baumannii and antimicrobial resistance. 2023;**59**(8):1386.

416. Correa A, Del Campo R, Escandón-Vargas K, Perenguez M, Rodríguez-Baños M, Hernández-Gómez C, et al. Distinct Genetic Diversity of Carbapenem-Resistant Acinetobacter baumannii from Colombian Hospitals. *Microb Drug Resist*. 2018;**24**(1):48-54. 10.1089/mdr.2016.0190

417. Cu-Uvin S, Hogan JW, Warren D, Klein RS, Peipert J, Schuman P, et al. Prevalence of lower genital tract infections among human immunodeficiency virus (HIV)—seropositive and high-risk HIV-seronegative women. 1999;**29**(5):1145-50.

418. Dumford DM, Skalweit MJIDC. Antibiotic-resistant infections and treatment challenges in the immunocompromised host. 2016;**30**(2):465-89.

419. Falodun OI, Musa IB, Oyelade AAJGR. Prevalence of extended beta-lactamase-producing Pseudomonas aeruginosa isolated from human immunodeficiency virus patients in Ibadan, Nigeria. 2021;**23**:101159.

420. Gebre HA, Wami AA, Kebede ES, Yidnekachew M, Gebre M, Negash AA. Nasopharyngeal Staphylococcus aureus colonization among HIV-infected children in Addis Ababa, Ethiopia: antimicrobial susceptibility pattern and association with Streptococcus pneumoniae colonization. *Access Microbiol*. 2023;**5**(8). 10.1099/acmi.0.000557.v3

421. Glupczynski Y, Bogaerts P, Deplano A, Berhin C, Huang T-D, Van Eldere J, et al. Detection and characterization of class A extended-spectrum-β-lactamase-producing Pseudomonas aeruginosa isolates in Belgian hospitals. 2010;**65**(5):866-71.

422. Golparian D, Cole MJ, Sánchez-Busó L, Day M, Jacobsson S, Uthayakumaran T, et al. Antimicrobial-resistant Neisseria gonorrhoeae in Europe in 2020 compared with in 2013 and 2018: a retrospective genomic surveillance study. *Lancet Microbe*. 2024;**5**(5):e478-e88. 10.1016/s2666-5247(23)00370-1

423. Gomes MZR, de Oliveira RVC, Machado CR, da Conceicao MdS, de Souza CV, da Silva Lourenco MC, et al. Factors associated with epidemic multiresistant Pseudomonas aeruginosa infections in a hospital with AIDS-predominant admissions. 2012;**16**(3):219-25.

424. Gomes MZR, Machado CR, da Conceicao MdS, Ortega JA, Neves SMFM, da Silva Lourenco MC, et al. Outbreaks, persistence, and high mortality rates of multiresistant Pseudomonas aeruginosa infections in a hospital with AIDS-predominant admissions. 2011;**15**(4):312-22.

425. Hernández-Jiménez P, López-Medrano F, Fernández-Ruiz M, Silva JT, Corbella L, San-Juan R, et al. Risk factors and outcomes for multidrug resistant Pseudomonas aeruginosa infection in immunocompromised patients. 2022;**11**(11):1459.

426. Kaase M, Szabados F, Pfennigwerth N, Anders A, Geis G, Pranada AB, et al. Description of the metallo-β-lactamase GIM-1 in Acinetobacter pittii. 2014;**69**(1):81-4.

427. Kahsay AG, Mezgebo TA, Gebrekidan GB, Desta BL, Mihretu HG, Dejene TA. Prevalence, Antibiotic Resistance and Associated Factors of Neisseria gonorrhoeae Among Patients Attending Non-Profitable Private Clinics in Mekelle, Tigrai, Ethiopia. *Infect Drug Resist*. 2023;**16**:4065-72. 10.2147/idr.S416344

428. Kengne MF, Mbaveng AT, Karimo O, Dadjo BS, Tsobeng OD, Marbou WJ, et al. Frequency of Fecal Carriage of ESBL Resistance Genes in Multidrug‐Resistant Pseudomonas aeruginosa Isolates From Cancer Patients at Laquintinie Hospital, Douala, Littoral Region, Cameroon. 2024;**2024**(1):7685878.

429. Lawung R, Prachayasittikul V, Bülow LJPE, Purification. Purification and Characterization of a β-Lactamase from Haemophilus ducreyi in Escherichia coli. 2001;**23**(1):151-8.

430. Manyahi J, Majigo M, Kibwana U, Kamori D, Lyamuya EFJIPiP. Colonization of Extended-spectrum β-lactamase producing Enterobacterales and meticillin-resistant S. aureus in the intensive care unit at a tertiary hospital in Tanzania: Implications for Infection control and prevention. 2022;**4**(2):100212.

431. Mimoz O, Elhelali N, Léotard S, Jacolot A, Laurent F, Samii K, et al. Treatment of experimental pneumonia in rats caused by a PER-1 extended-spectrum β-lactamase-producing strain of Pseudomonas aeruginosa. 1999;**44**(1):91-7.

432. Moghadam MN, Motamedifar M, Sarvari J, Sedigh ES, Mousavi SM, Moghadam FN. Emergence of Multidrug Resistance and Metallo-beta-lactamase Producing Acinetobacter baumannii Isolated from Patients in Shiraz, Iran. *Ann Med Health Sci Res*. 2016;**6**(3):162-7. 10.4103/2141-9248.183946

433. Montaña S, Lazzaro T, Uong S, Place K, Iriarte A, Ocampo C, et al. Genomics helps to decipher the resistance mechanisms present in a Pseudomonas chlororaphis strain recovered in an HIV patient. 2018;**25**:45.

434. Montaña S, Lazzaro T, Uong S, Place K, Iriarte A, Ocampo CV, et al. Genomics helps to decipher the resistance mechanisms present in a Pseudomonas chlororaphis strain recovered in an HIV patient. *New Microbes New Infect*. 2018;**25**:45-7. 10.1016/j.nmni.2018.07.002

435. Moya B, Barcelo IM, Bhagwat S, Patel M, Bou G, Papp-Wallace KM, et al. WCK 5107 (Zidebactam) and WCK 5153 Are Novel Inhibitors of PBP2 Showing Potent "β-Lactam Enhancer" Activity against Pseudomonas aeruginosa, Including Multidrug-Resistant Metallo-β-Lactamase-Producing High-Risk Clones. *Antimicrob Agents Chemother*. 2017;**61**(6). 10.1128/aac.02529-16

436. Naas T, Benaoudia F, Lebrun L, Nordmann PJEJoCM, Diseases I. Molecular identification of TEM-1 β-lactamase in a Pasteurella multocida isolate of human origin. 2001;**20**:210-3.

437. Obritsch MD, Fish DN, MacLaren R, Jung RJPTJoHP, Therapy D. Nosocomial infections due to multidrug‐resistant Pseudomonas aeruginosa: epidemiology and treatment options. 2005;**25**(10):1353-64.

438. Rodrigues DO, Filho PPG, Tavares M, Costa-Pinto RMJIJoM, Sciences M. Risk factors for ventilation associated-pneumonia by multidrug-resistant Pseudomonas aeruginosa producing metallo-β-lactamase. 2010;**2**(9):271-6.

439. Samie A, Shivambu NJAJoB. Biofilm production and antibiotic susceptibility profiles of Staphylococcus aureus isolated from HIV and AIDS patients in the Limpopo Province, South Africa. 2011;**10**(65):14625-36.

440. Sarhaddi N, Soleimanpour S, Farsiani H, Mosavat A, Dolatabadi S, Salimizand H, et al. Elevated prevalence of multidrug-resistant Acinetobacter baumannii with extensive genetic diversity in the largest burn centre of northeast Iran. *J Glob Antimicrob Resist*. 2017;**8**:60-6. 10.1016/j.jgar.2016.10.009

441. Shi J, Mao X, Cheng J, Shao L, Shan X, Zhu YJFiC, et al. Risk factors and predictive model for nosocomial infections by extensively drug-resistant Acinetobacter baumannii. 2024;**14**:1475428.

442. Soltani B, Heidari H, Ebrahim-Saraie HS, Hadi N, Mardaneh J, Motamedifar M. Molecular characteristics of multiple and extensive drug-resistant Acinetobacter baumannii isolates obtained from hospitalized patients in Southwestern Iran. *Infez Med*. 2018;**26**(1):67-76.

443. Tilahun M, Gedefie A, Sahle Z. Asymptomatic Carriage Rate, Multidrug Resistance Level, and Associated Risk Factors of Enterococcus in Clinical Samples among HIV-Positive Patients Attending at Debre Birhan Comprehensive Specialized Hospital, North Showa, Ethiopia. *Biomed Res Int*. 2023;**2023**:7310856. 10.1155/2023/7310856

444. Weldhagen GF, Prinsloo AJIjoaa. Molecular detection of GES-2 extended spectrum β-lactamase producing Pseudomonas aeruginosa in Pretoria, South Africa. 2004;**24**(1):35-8.

445. Adeyemi FM, Ako-nai KA, Adejuyigbe E, Ebhodaghe BI, Osho PO, Oyeniyi TT, et al. Molecular characterization and antibiotic resistance profiles of bacterial isolates cultured from HIV seropositive patients. 2015;**6**(1).

446. Agwu E, Ihongbe JC, Ezeonwumelu JO, Lodhi MMJOSI. Baseline burden and antimicrobial susceptibility of pathogenic bacteria recovered from oral lesions of patients with HIV/AIDS in South-Western Uganda. 2015;**12**(2):59-66.

447. Aina O, Ajayi-Odoko OA. Multidrug resistance profile and extended-spectrum beta-lactamase production in faecal escherichia coli isolated from hiv and tb patients in Ekiti-State, Nigeria. 2019.

448. Arirachakaran P, Luangworakhun S, Charalampakis G, Dahlén G. Non-oral, aerobic, Gram-negative bacilli in the oral cavity of Thai HIV-positive patients on Highly-active anti-retrovirus therapy medication. *J Investig Clin Dent*. 2019;**10**(2):e12387. 10.1111/jicd.12387

449. Badmus AR. Prevalence and Antimicrobial Susceptibility Patterns of Asymptomatic Bacteriuria in HIV Positive Patients in Ilorin, Kwara State, Nigeria: Kwara State University (Nigeria); 2019.

450. Henderson HI, Napravnik S, Gower EW, Aiello AE, Kinlaw AC, Williams B, et al. Resistance in Enterobacterales is higher among people living with human immunodeficiency virus. 2022;**75**(1):28-34.

451. Iyamba J-ML, Wambale JMJTPAMJ. Antimicrobial susceptibility patterns of enterobacteriaceae isolated from HIV-infected patients in Kinshasa. 2014;**17**:179.

452. Kahsay T, Gebrehiwot GT, Gebreyohannes G, Tilahun M, Gessese A, Kahsay A. Antimicrobial susceptibility patterns of urinary tract infections causing bacterial isolates and associated risk factors among HIV patients in Tigray, Northern Ethiopia. *BMC Microbiol*. 2024;**24**(1):148. 10.1186/s12866-024-03297-2

453. Kumar MRR, Arunagirinathan N, Vignesh R, Balakrishnan P, Solomon S, Sunil SSJJoRiMS. Ertapenem for multiple β-lactamases producing Gram-negative bacteria causing urinary tract infections in HIV patients. 2017;**22**(1):69.

454. Kumar V, Murali S, Goldberg J, Alonso B, Moretó-Planas L, Reid A, et al. Antibiotic susceptibility patterns of pathogens isolated from hospitalized patients with advanced HIV disease (AHD) in Bihar, India. *JAC Antimicrob Resist*. 2024;**6**(1):dlad151. 10.1093/jacamr/dlad151

455. Yezli S, Shibl AM, Memish ZAJJomm. The molecular basis of β-lactamase production in Gram-negative bacteria from Saudi Arabia. 2015;**64**(2):127-36.

456. Adeyemi FM, Oyedara OO, Wahab AA, Akinde SBJAJoCM, Infection. ESβL and MβL Production in Gram-Negative Bacteria Isolated From HIV Seropositive Individuals. 2023;**10**(1):1-8.

457. Ali MA, Okojokwu OJ, Augustine UA, Achenbach C, AjeAnejo-Okopi J, MankoLar P, et al. Prevalence and drug-resistance profile of plasmid-borne extended spectrum beta-lactamase (ESBLs) resistance genes in multidrug resistant Escherichia coli from HIV-1 positive individuals in Jos, Nigeria. 2020;**14**(10):564-71.

458. Olaru ID, Ferrand RA, Chisenga M, Yeung S, Macrae B, Chonzi P, et al. Prevalence of ESBL-producing Escherichia coli in adults with and without HIV presenting with urinary tract infections to primary care clinics in Zimbabwe. 2021;**3**(2):dlab082.

459. Omo-Omorodion B, Evurani S, Emencheta S, Okpalanwa C, Eze O, Ozioko AJEAMJ. Extended-spectrum Beta-lactamase and plasmid profiling of uropathogens cultured from HIV seropositive pregnant women and controls in Akure, south-western Nigeria. 2021;**98**(12).

460. Padmavathy K, Padma K, Rajasekaran SJJomm. Extended-spectrum β-lactamase/AmpC-producing uropathogenic Escherichia coli from HIV patients: do they have a low virulence score? 2013;**62**(3):345-51.

461. Udoh DI, Utsalob SJ, Asuquob AJEJoB. Antibiotics resistance profile and extended spectrum beta-lactamases (ESBLs) production by Salmonella species isolated from HIV/AIDS subjects in Akwa Ibom State, Nigeria. 2018;**5**(2):7-13.

462. Bayleyegn B, Fisaha R, Kasew D. Fecal carriage of extended spectrum beta-lactamase producing Enterobacteriaceae among HIV infected children at the University of Gondar Comprehensive Specialized Hospital Gondar, Ethiopia. *AIDS Research and Therapy*. 2021;**18**:1-9.

463. Dimani BD, Founou RC, Zemtsa JR, Mbossi A, Koudoum PL, Founou LL, et al. Faecal carriage of multidrug-resistant and extended-spectrum β-lactamase-producing Enterobacterales in people living with HIV in Yaoundé, Cameroon. *Journal of Global Antimicrobial Resistance*. 2023;**35**:26-34.

464. Endalamaw D, Amsalu A, Biset S, Mekonnen F, Balew M, Eshetie S. Extended-Spectrum β-Lactamases-Producing Enterobacteria and Antimicrobial Resistance Pattern among HIV/AIDS Patients in the University of Gondar Specialized Hospital, Ethiopia. *Recent Advances in Biology and Medicine*. 2020;**6**(1):11909.

465. Falodun OI, Ajayi O, Ademola AE, Bakarey SA. Antibiotic Susceptibility Patterns and Extended Spectrum Beta-Lactamase (ESBL) production in Enterobactericeae Isolated from Stool Samples of HIV and AIDS Patients in Ibadan, Nigeria. *Janaki Medical College Journal of Medical Science*. 2021;**9**(1):5-15.

466. Hosuru Subramanya S, Bairy I, Nayak N, Padukone S, Sathian B, Gokhale S. Low rate of gut colonization by extended-spectrum β-lactamase producing Enterobacteriaceae in HIV infected persons as compared to healthy individuals in Nepal. *PLoS One*. 2019;**14**(2):e0212042.

467. Jerry A, Ebele U, Tersagh S, Mwuese G. Prevalence of ESBL-producing and multi-drug resistant Escherichia coli isolated from urine of HIV patients in General Hospital Gboko, Benue State. *World Journal of Biology Pharmacy Health Sciences*. 2021;**8**(1):029-36.

468. John-Onwe B, Iroha I, Moses I, Onuora A, Nwigwe J, Adimora E, et al. Prevalence and multidrug-resistant ESBL-producing E. coli in urinary tract infection cases of HIV patients attending Federal Teaching Hospital, Abakaliki, Nigeria. *African Journal of Microbiology Research*. 2022;**16**(5):196-201.

469. Maharjan R, Bastola A, Adhikari N, Rijal KR, Banjara MR, Ghimire P, et al. Multidrug-resistant bacteria with ESBL genes: a growing threat among people living with HIV/AIDS in Nepal. *BMC Infectious Diseases*. 2022;**22**(1):526.

470. Manyahi J, Moyo SJ, Tellevik MG, Langeland N, Blomberg B. High prevalence of fecal carriage of extended spectrum β-lactamase-producing Enterobacteriaceae among newly HIV-diagnosed adults in a community setting in Tanzania. *Microbial Drug Resistance*. 2020;**26**(12):1540-5.

471. Nwokolo CJ, Ugwu MC, Ejikeugwu CP, Iroha IR, Esimone CO. Incidence and antibiotic susceptibility profile of uropathogenic Escherichia coli positive for extended spectrum β-lactamase among HIV/AIDS patients in Awka metropolis, Nigeria. *Iranian Journal of Microbiology*. 2022;**14**(3):334.

472. Osazuwa F, Osazuwa E, Imade P, Dirisu J, Omoregie R, Okuonghae P, et al. Occurrence of extended spectrum producing gram negative bacteria in HIV/AIDS infected patients with urinary and gastrointestinal tract infections in Benin metropolis. *Research Journal of Pharmaceutical, Biological and Chemical Sciences*. 2011;**2**(2):230-34.

473. Padmavathy K KPaSR. Detection of Extended-Spectrum β Lactamases and AmpC β-Lactamase Production in Escherichia coli Causing Urinary Tract Infection among HIV and Non-HIV Patients. *American Medical Journal*. 2011;**2**(1):54-8.

474. Rameshkumar MR, Arunagirinathan N, Senthamilselvan B, Swathirajan CR, Solomon SS, Vignesh R, et al. Occurrence of extended-spectrum β-lactamase, AmpC, and carbapenemase-producing genes in gram-negative bacterial isolates from human immunodeficiency virus infected patients. *Journal of Infection and Public Health*. 2021;**14**(12):1881-6.

475. Reinheimer C, Keppler OT, Stephan C, Wichelhaus TA, Friedrichs I, Kempf VA. Elevated prevalence of multidrug-resistant gram-negative organisms in HIV positive men. *BMC Infectious Diseases*. 2017;**17**:1-6.

476. Said MM, Msanga DR, Mtemisika CI, Silago V, Mirambo MM, Mshana SE. Extended spectrum β-lactamase producing lactose fermenting bacteria colonizing children with human immunodeficiency virus, sickle cell disease and diabetes mellitus in Mwanza City, Tanzania: A cross-sectional study. *Tropical Medicine Infectious Disease*. 2022;**7**(8):144.

477. Simeneh E, Gezimu T, Woldemariam M, Alelign D. Magnitude of multidrug-resistant bacterial uropathogens and associated factors in urinary tract infection suspected adult HIV-positive patients in Southern Ethiopia. *The Open Microbiology Journal*. 2022;**16**(1).

478. Singh M, Chakraborty A. Prevalence of Extended Spectrum β-Lactamase Genes among the Oral Gram Negative Rods Isolated from HIV Infected Patients. *Indian Journal of Public Health*. 2020;**11**(6):505.

479. Surgers L, Chiarabini T, Royer G, Rougier H, Mercier-Darty M, Decré D, et al. Evidence of Sexual Transmission of Extended-Spectrum β-Lactamase–Producing Enterobacterales: A Cross-sectional and Prospective Study. *Clinical Infectious Diseases*. 2022;**75**(9):1556-64.

480. Wilmore SS, Kranzer K, Williams A, Makamure B, Nhidza A, Mayini J, et al. Carriage of extended-spectrum beta-lactamase-producing Enterobacteriaceae in HIV-infected children in Zimbabwe. *Journal of medical microbiology*. 2017;**66**(5):609-15.

481. Zemtsa RJ, Noubom M, Founou LL, Dimani BD, Koudoum PL, Mbossi AD, et al. Multidrug-Resistant and Extended-Spectrum β-Lactamase (ESBL)-Producing Enterobacterales Isolated from Carriage Samples among HIV Infected Women in Yaoundé, Cameroon. *Pathogens*. 2022;**11**(5):504.
