# Supplementary material for: Extended-spectrum β-lactamase-producing Enterobacterales among people living with human immunodeficiency virus across the globe: A systematic review and meta-analysis
Source: PLoS One. 2025 Jun 10;20(6):e0321873. doi: 10.1371/journal.pone.0321873 (PMC12151346; doi:10.1371/journal.pone.0321873)
Supplement: Attachment — Submitted filename: Response to Reviewers.docx [file pone.0321873.s008.docx]

PONE-D-24-40800

Extended-spectrum β-lactamase-producing gram-negative bacilli among people living with human immunodeficiency virus across the globe: a systematic review and meta-analysis

PLOS ONE

Dear Dr. Tigabie,

Thank you for submitting your manuscript to PLOS ONE. After careful consideration, we feel that it has merit but does not fully meet PLOS ONE’s publication criteria as it currently stands. Therefore, we invite you to submit a revised version of the manuscript that addresses the points raised during the review process.

Please submit your revised manuscript by May 14 2025 11:59PM. If you will need more time than this to complete your revisions, please reply to this message or contact the journal office at [plosone@plos.org](mailto:plosone@plos.org). When you're ready to submit your revision, log on to <https://www.editorialmanager.com/pone/> and select the 'Submissions Needing Revision' folder to locate your manuscript file.

Please include the following items when submitting your revised manuscript:

- A rebuttal letter that responds to each point raised by the academic editor and reviewer(s). You should upload this letter as a separate file labeled 'Response to Reviewers'.
- A marked-up copy of your manuscript that highlights changes made to the original version. You should upload this as a separate file labeled 'Revised Manuscript with Track Changes'.
- An unmarked version of your revised paper without tracked changes. You should upload this as a separate file labeled 'Manuscript'.

If you would like to make changes to your financial disclosure, please include your updated statement in your cover letter. Guidelines for resubmitting your figure files are available below the reviewer comments at the end of this letter.

If applicable, we recommend that you deposit your laboratory protocols in [protocols.io](http://protocols.io/) to enhance the reproducibility of your results. Protocols.io assigns your protocol its own identifier (DOI) so that it can be cited independently in the future. For instructions see: [https://journals.plos.org/plosone/s/submission-guidelines#loc-laboratory-protocols](https://track.editorialmanager.com/CL0/https:%2F%2Fjournals.plos.org%2Fplosone%2Fs%2Fsubmission-guidelines%23loc-laboratory-protocols/1/010f0195ea50aad2-f6acb741-8afa-40cb-8327-845344575b58-000000/_UfQDlwePJP5TbxLODNiCYU1nCbA8QdzxD6s89JXXfo=204). Additionally, PLOS ONE offers an option for publishing peer-reviewed Lab Protocol articles, which describe protocols hosted on [protocols.io](http://protocols.io/). Read more information on sharing protocols at [https://plos.org/protocols?utm_medium=editorial-email&utm_source=authorletters&utm_campaign=protocols](https://track.editorialmanager.com/CL0/https:%2F%2Fplos.org%2Fprotocols%3Futm_medium=editorial-email%26utm_source=authorletters%26utm_campaign=protocols/1/010f0195ea50aad2-f6acb741-8afa-40cb-8327-845344575b58-000000/tAF5XyQAjLNJM7g636avnmD7nN18mNa9UK8T0wI1uw4=204).

We look forward to receiving your revised manuscript.

Kind regards,

Amir Nutman
Academic Editor
PLOS ONE

**Response to Academic Editor, Reviewers as well as Journal Requirements:**

**Authors: We appreciate for spending your precious time and forwarding your valuable comments, which have significantly improved our manuscript. We are also grateful for this positive feedback. Please see below, bold, for a point-by-point response to the reviewers. We've copied your comments and responses below to make things easier for you. All line numbers refer to the revised manuscript file.**

Journal Requirements:

When submitting your revision, we need you to address these additional requirements.

1.Please ensure that your manuscript meets PLOS ONE's style requirements, including those for file naming. The PLOS ONE style templates can be found at

<https://journals.plos.org/plosone/s/file?id=wjVg/PLOSOne_formatting_sample_main_body.pdf> and

[https://journals.plos.org/plosone/s/file?id=ba62/PLOSOne_formatting_sample_title_authors_affiliations.pdf](https://track.editorialmanager.com/CL0/https:%2F%2Fjournals.plos.org%2Fplosone%2Fs%2Ffile%3Fid=ba62%2FPLOSOne_formatting_sample_title_authors_affiliations.pdf/1/010f0195ea50aad2-f6acb741-8afa-40cb-8327-845344575b58-000000/o16F5NXkFz9tSQ8IoG81xun3Lna1tcb3A-jFLtpBtRM=204)

**Authors: we have prepared the manuscript based on PLOS ONE's style requirements.**

2. We note that your Data Availability Statement is currently as follows: All relevant data are within the manuscript and in Supporting Information files.

Please confirm at this time whether or not your submission contains all raw data required to replicate the results of your study. Authors must share the “minimal data set” for their submission. PLOS defines the minimal data set to consist of the data required to replicate all study findings reported in the article, as well as related metadata and methods (<https://journals.plos.org/plosone/s/data-availability#loc-minimal-data-set-definition>).

For example, authors should submit the following data:

- The values behind the means, standard deviations and other measures reported;

- The values used to build graphs;

- The points extracted from images for analysis.

Authors do not need to submit their entire data set if only a portion of the data was used in the reported study.

If your submission does not contain these data, please either upload them as Supporting Information files or deposit them to a stable, public repository and provide us with the relevant URLs, DOIs, or accession numbers. For a list of recommended repositories, please see <https://journals.plos.org/plosone/s/recommended-repositories>.

If there are ethical or legal restrictions on sharing a de-identified data set, please explain them in detail (e.g., data contain potentially sensitive information, data are owned by a third-party organization, etc.) and who has imposed them (e.g., an ethics committee). Please also provide contact information for a data access committee, ethics committee, or other institutional body to which data requests may be sent. If data are owned by a third party, please indicate how others may request data access.

**Authors: we have summited all in Supporting Information files.**

3. When completing the data availability statement of the submission form, you indicated that you will make your data available on acceptance. We strongly recommend all authors decide on a data sharing plan before acceptance, as the process can be lengthy and hold up publication timelines. Please note that, though access restrictions are acceptable now, your entire data will need to be made freely accessible if your manuscript is accepted for publication. This policy applies to all data except where public deposition would breach compliance with the protocol approved by your research ethics board. If you are unable to adhere to our open data policy, please kindly revise your statement to explain your reasoning and we will seek the editor's input on an exemption. Please be assured that, once you have provided your new statement, the assessment of your exemption will not hold up the peer review process.

**Authors: we all authors decide and agreed on PLOS ONE's data sharing plan. All authors decide and agreed data availability statement.**

4. Your ethics statement should only appear in the Methods section of your manuscript. If your ethics statement is written in any section besides the Methods, please move it to the Methods section and delete it from any other section. Please ensure that your ethics statement is included in your manuscript, as the ethics statement entered into the online submission form will not be published alongside your manuscript.

**Authors: we provided ethics statement in the Methods section of the manuscript.**

5. Please include captions for your Supporting Information files at the end of your manuscript, and update any in-text citations to match accordingly. Please see our Supporting Information guidelines for more information: [http://journals.plos.org/plosone/s/supporting-information](http://track.editorialmanager.com/CL0/http:%2F%2Fjournals.plos.org%2Fplosone%2Fs%2Fsupporting-information/1/010f0195ea50aad2-f6acb741-8afa-40cb-8327-845344575b58-000000/lKczvLPAXYvoVwhiO9WiVJ3vqvJKjiPBfOSXLXwRi6Y=204).

**Authors: we provided captions for the Supporting Information files at the end of the manuscript**

6. As required by our policy on Data Availability, please ensure your manuscript or supplementary information includes the following:

A numbered table of all studies identified in the literature search, including those that were excluded from the analyses.

For every excluded study, the table should list the reason(s) for exclusion.

If any of the included studies are unpublished, include a link (URL) to the primary source or detailed information about how the content can be accessed.

A table of all data extracted from the primary research sources for the systematic review and/or meta-analysis. The table must include the following information for each study:

Name of data extractors and date of data extraction

Confirmation that the study was eligible to be included in the review.

All data extracted from each study for the reported systematic review and/or meta-analysis that would be needed to replicate your analyses.

If data or supporting information were obtained from another source (e.g. correspondence with the author of the original research article), please provide the source of data and dates on which the data/information were obtained by your research group.

If applicable for your analysis, a table showing the completed risk of bias and quality/certainty assessments for each study or outcome.  Please ensure this is provided for each domain or parameter assessed. For example, if you used the Cochrane risk-of-bias tool for randomized trials, provide answers to each of the signalling questions for each study. If you used GRADE to assess certainty of evidence, provide judgements about each of the quality of evidence factor. This should be provided for each outcome.

An explanation of how missing data were handled.

This information can be included in the main text, supplementary information, or relevant data repository. Please note that providing these underlying data is a requirement for publication in this journal, and if these data are not provided your manuscript might be rejected.

**Authors: we provided a table that included all studies identified in the literature search, along with reason(s) for exclusion for every excluded study. We uploaded this table in the Supporting Information files.**

**We also, provided a table showing the completed risk of bias as well as Name of data extractors and date of data extraction for included studies.**

Additional Editor Comments:

Dear Authors,

Thank you for submitting your manuscript, “Extended-spectrum β-lactamase-producing gram-negative bacilli among people living with human immunodeficiency virus across the globe: a systematic review and meta-analysis” to PLOS ONE. Based on the reviewers’ comments, I invite you to submit a revised version.

Please address all reviewer points in a point-by-point response and highlight changes in the manuscript. This decision does not guarantee acceptance, but I believe the work has potential pending revision.

I look forward to your resubmission.

Best regards,
Amir

[Note: HTML markup is below. Please do not edit.]

**Response to Academic Editor**

**Authors: Thank you for your positive feedback; we appreciate your feedback. We have revised the entire manuscript as necessary and have attempted to address the comments from the reviewers.**

Reviewers' comments:

Reviewer's Responses to Questions

**Comments to the Author**

1. Is the manuscript technically sound, and do the data support the conclusions?

The manuscript must describe a technically sound piece of scientific research with data that supports the conclusions. Experiments must have been conducted rigorously, with appropriate controls, replication, and sample sizes. The conclusions must be drawn appropriately based on the data presented.

Reviewer #1: No

Reviewer #2: Partly

2. Has the statistical analysis been performed appropriately and rigorously?

Reviewer #1: Yes

Reviewer #2: Yes

3. Have the authors made all data underlying the findings in their manuscript fully available?

The [PLOS Data policy](http://track.editorialmanager.com/CL0/http:%2F%2Fwww.plosone.org%2Fstatic%2Fpolicies.action%23sharing/1/010f0195ea50aad2-f6acb741-8afa-40cb-8327-845344575b58-000000/9FpY3-IZoSGvYyLj7WSSTcldRBW7ER-TfANFqQG_JwE=204) requires authors to make all data underlying the findings described in their manuscript fully available without restriction, with rare exception (please refer to the Data Availability Statement in the manuscript PDF file). The data should be provided as part of the manuscript or its supporting information, or deposited to a public repository. For example, in addition to summary statistics, the data points behind means, medians and variance measures should be available. If there are restrictions on publicly sharing data—e.g. participant privacy or use of data from a third party—those must be specified.

Reviewer #1: Yes

Reviewer #2: Yes

4. Is the manuscript presented in an intelligible fashion and written in standard English?

PLOS ONE does not copyedit accepted manuscripts, so the language in submitted articles must be clear, correct, and unambiguous. Any typographical or grammatical errors should be corrected at revision, so please note any specific errors here.

Reviewer #1: Yes

Reviewer #2: Yes

5. Review Comments to the Author

Please use the space provided to explain your answers to the questions above. You may also include additional comments for the author, including concerns about dual publication, research ethics, or publication ethics. (Please upload your review as an attachment if it exceeds 20,000 characters)

**Response to Reviewers**

**Authors: we would like to say thank you for reviewing our work and making insightful suggestions and comments that helped to strengthen our manuscript. We have revised the manuscript as necessary.**

**Reviewer #1:** The authors conducted a systematic review and meta-analysis on the prevalence of ESBL in Gram negative bacteria among people with HIV. While the methods used for the analysis are appropriate, I have several concerns with regards to how the data are interpreted and reported.

**Authors: Thank you for your positive feedback.**

**Reviewer #1:** Firstly, the main results on the prevalence of ESBL should be reported according to bacterial species and type (colonisation vs. infection). It is known that there are differences in ESBL prevalence between common Gram negatives such as E. coli and K. pneumoniae and pooling results across organisms is less meaningful. Further the authors should present as a main analysis (instead of a subgroup) the prevalence of ESBL in colonisation and infection samples.

**Authors: Thank you for bringing this issue to our intention. We have revised the analysis according to bacterial species and type (colonisation vs. infection). (Please refer to the revised manuscript's result section line # 248-272 and** Supplementary File 4, Fig**).**

**Reviewer #1:** Secondly the authors should note that for A. baumanii and P. aeruginosa, there are other mechanisms that play a more important role in beta-lactam resistance than ESBL-production. For this reason, the authors should restrict the focus of the manuscript on Enterobacterales and exclude A baumanii and P. aeruginosa.

**Authors: Thank you for your positive feedback. We have reanalyzed the data after excluding *A baumanii* and *P. aeruginosa, now* the manuscript focused on only about Enterobacterales (Please refer to the revised manuscript).**

**Reviewer #1:** Lastly, the authors should revise the manuscript to improve clarity and avoid repetitions. More specific comments are added below.

**Authors: Thank you for your input. We have accepted your comment and we tried to amend to improve clarity and avoid repetitions (Please refer to the revised manuscript).**

**Reviewer #1:** Lines 62-69: use concordant referencing. Currently you are reporting on data from various sources and the figures are somewhat conflicting. The O’Neill report which is almost 10 years old may have overestimated the number of deaths due to AMR. The paragraph would be easier to read if only one set of figures per point made would be used. Also in line 66 the sentence moves from economic losses in the US to global economic losses which is confusing.

**Authors: Thank you for raising this interesting point. We have changed the whole paragraph with updated data. (Please refer to the revised manuscript line # 65-72).**
**Reviewer #1:** Line 72: “Furthermore, the surge of infections that cause immune suppression, such as human immunodeficiency virus “consider rephrasing as the infection you are referring to is HIV and there is no surge of multiple infections associated with immunosuppression. (unless you mean conditions associated with immunosuppression e.g. long-term steroids etc.

**Authors: We appreciate your feedback. We have rephrased and corrected a typing error (Please refer to the revised manuscript line # 75-79).**

**Reviewer #1:** Lines 82-84: introduce the epidemiology of HIV before introducing how it relates to resistance development

**Authors: Thank you for raising this interesting point. We incorporated the epidemiology of HIV before introducing how it relates to resistance development. (Please refer to the revised manuscript line # 84-90).**

**Reviewer #1:** Line 90: “Among bacterial pathogens, gram negative bacilli are the most dangerous …” while it is true that gram-negative organisms pose more problems because of their resistance to antibiotics, they are not necessarily more dangerous (unless a reference is provided to support that statement e.g. that bacterial infections due to gram-negatives cause more deaths).

**Authors: Thank you for your comment. We have removed the phrase to avoid confusion. (Please refer to the revised manuscript line # 95-96).**

**Reviewer #1:** Line 95: “cephalosporins, and aztreonam, which are easily available” remove aztreonam from the listing as it is not easily available and commonly used

**Authors: We appreciate your feedback. We have removed aztreonam from the listing based on your recommendation. (Please refer to the revised manuscript line # 99-101).**

**Reviewer #1:** Line 97: “Enterobacteriaceae, Pseudomonas aeruginosa (P. aeruginosa), and Acinetobacter baumannii (A. baumannii) are the predominant gram-negative bacilli that produce ESBL enzymes “ please revise the sentence – the main mechanisms associated with resistance for P aeruginosa and A. baumanii are not ESBL-production.

**Authors: Thank you for your input. We have revised the sentence via focusing on Enterobacteriaceae. (Please refer to the revised manuscript line # 102&103).**

**Reviewer #1:** Lines 105-107 – “the number of ESBL-producing gram-negative bacilli among HIV-positive individuals varies from 2.3% to 65.6%” – these are proportions not numbers

**Authors: Thank you for your positive feedback. We have amended to proportions.**
**Reviewer #1:** Lines 109-110: “On the one hand, various studies in the past reported higher ESBLs among HIV-positive individuals [20-29]. On the other hand, some studies reported that ESBL producers were more common among HIV-negative patients than among HIV-positive individuals [30-32].” – please consolidate these sentences to avoid repetitions

**Authors: Thank you for your input. We have modified to avoid repetitions. (Please refer to the revised manuscript line # 111&113).**
**Reviewer #1:** Line 139: please clarify in the methods whether the review included studies reporting exclusively on people living with HIV or it also included studies with mixed populations which reported on AMR prevalence by HIV status

**Authors: Thank you for reflecting on your concern. We have included studies with mixed populations which reported on ESBL-producing *Enterobacterales* prevalence by HIV status. We have written this sentence from eligibility criteria. Please refer to the revised manuscript line # 150 &151).**

**Reviewer #1:** Line 157: “The three reviewers listed above, MT, KT, and AB, were independently assessed to assess the quality of the included studies.” Please revise to avoid repetition

**Authors: We have accepted your comment and modified it avoid repetition.**
**Reviewer #1:** Figure 1: conventionally in the PRISMA diagram, the studies are reported separately as to how many were excluded in the title and abstract screening (reason for exclusion not necessary) and how many were excluded in the full text phase (with reason for exclusion).

**Authors: Thank you for your comment. We have reconstructed the diagram and revised the text to provide a clearer explanation and resolve any ambiguity. We have provided how many were excluded in the title and abstract screening (without reason for exclusion) and how many were excluded in the full text phase (with reason for exclusion) based on your comment. (Please refer to the revised manuscript line Fig. 1).**

**Reviewer #1:** Line 214 and above: be specific in the methods on how the prevalence was calculated for case-control studies (depending on the study design, might only be possible to calculate prevalence in the cases)

**Authors: Thank you for your comments and questions. For case-control studies, the prevalence was calculated only among the cases. We have included this sentence from method section. Please refer to the revised manuscript line # 140 &141).**
**Reviewer #1:** Table 1: please check and/or comment on including studies on infection which reported on stool samples. In this setting ESBL-producing organisms are also likely to represent colonisation (e.g. the study by Falodun reference #23 et al is clearly reporting on colonisation). Also check as there is an error with reference #22 which is a different study by Falodun et al. (not Israel)

**Authors: Thank you for bringing this issue to our intention. We have revised the analysis after removing the study by Falodun reference #23 et al, because this study focused on only Pseudomonas that was why we considered as infection. We have replaced study reference #22 by Falodun et al. because Israel is the middle name for Falodun. Thank you again for raising this interesting point. Please refer to the revised manuscript table 1).**

**Reviewer #1:** Line 231: please note that it is not appropriate to pool prevalence across very different bacterial species and sample types. At least the pooling should be done separately for colonisation and infection and ideally separate for the main Enterobacterales species. This should not be treated as a subgroup analysis but rather the main analysis. I would suggest that you omit Pseudomonas and Acinetobacter completely from the analysis and manuscript as these organisms have additional mechanisms causing beta-lactam resistance, other than ESBL.

**Authors: Thank you for your input. We have removed Pseudomonas and Acinetobacter completely from the analysis and manuscript. We have modified the whole analysis and re-written manuscript.**
**Reviewer #1:** Table 2: “Rate of ESBL…” heading – please rename as this is not a rate (prevalence).

**Authors: Thank you for reflecting on your concern. We have removed the table and provided the results with a forest plot for each isolate, which has been uploaded as Supplementary File 4. Please refer to the Supplementary File 4).**

**Reviewer #1:** Table 2: Comment separately in the manuscript text on the prevalence of ESBL in Salmonella – 20% is quite high and concerning for clinical practice.

**Authors: Thank you for your positive feedback. We have written separately in the manuscript text on the prevalence of ESBL in *Salmonella* spp. Please refer to the revised manuscript line # 261 &263).**
Table 3: can be omitted

**Authors: Thank you for reflecting on your concern. We have omitted Table 3 and the other publication bias analysis results from the main manuscript; instead, we have provided them in Supplementary File 3. (Please refer to the Supplementary File 3).**

**Reviewer #1:** Figure 3 should be redrawn as it is difficult to read with a black background. Also the numbers on the x-axis are overlapping. The figure can be included in the supplement.

**Authors: We appreciate your feedback. We have redrawn Figure 3 and the table for sensitivity analysis and we have provided them in Supplementary File 3. (Please refer to the Supplementary File 3).**

**Reviewer #1:** Line 279: the subgroup analysis on method used can be omitted (or included in the supplement). For the other subgroup analyses please see my comment above. The authors could also add a subgroup analysis by organism and type (colonisation vs. infection) for community-acquired and hospital associated infections

**Authors: Thank you for reflecting on your concern. We included a subgroup analysis by age group; however, subgroup analysis by community-acquired and hospital associated infections not performed because there was no data from primary studies. Subgroup analysis by organism and type (colonisation vs. infection) are less important, as it may be cumbersome and the pooled prevalence for each strain and type have already been performed (Please refer to the supplementary file 5)**.
**Reviewer #1:** Table 5 and Supplement: the genes should be reported according to the bacterial species in which they were identified (consider reporting for E. coli and K. pneumoniae only). Please also specify which SHV/CTX-M/TEM/OXA genes were reported as not all of them are ESBL

**Authors: Thank you for bringing this issue to our intention. We have reported the genes according to the bacterial species in which they were identified. Please refer to the revised manuscript line table 3).**
**Reviewer #1:** Line 322: please rephrase. The organisms are not multidrug resistant because they are ESBL-producers (there is a somewhat old definition of MDR and being ESBL does not suffice).

**Authors: Thank you for your input. We have accepted your comment and rephrase this paragraph. (Please refer to the discussion portion of revised manuscript)**

**Reviewer #1:** Line 339: please revise as E. coli is a common cause of community-acquired infections

**Authors: Thank you for bringing this issue to our intention. We have revised the sentence based on your comment.**
**Reviewer #1:** Line 377: the higher prevalence may be explained by the methods used to report on colonisation (denominators, method of detection – culture media).

**Authors: Thank you for your positive feedback. We attempted to discuss according to your suggestion.**
**Reviewer #1:** Lines 401-410: paragraph has repetitive sentences please revise

**Authors: Thank you for your input. We have accepted your comment and we tried to amend it to avoid repetition.**

**Reviewer #2:** This systematic review and meta-analysis provide valuable insights into ESBL prevalence among HIV-positive individuals. While the study is methodologically strong, improvements in data selection, bias reduction, and clinical interpretation could enhance its impact. Future research should focus on treatment outcomes, stewardship interventions, and emerging resistance mechanisms to inform better infection control policies in HIV care settings.The study adheres to PRISMA guidelines, ensuring methodological transparency. It addresses a critical intersection of HIV and antimicrobial resistance (AMR), a growing concern for global health.

**Authors**: **we would like to say thank you for reviewing our work and making insightful suggestions and comments that helped to strengthen our manuscript. We have revised the manuscript as necessary.**
