# Supplementary material for: Extended-spectrum β-lactamase-producing Enterobacterales among people living with human immunodeficiency virus across the globe: A systematic review and meta-analysis
Source: PLoS One. 2025 Jun 10;20(6):e0321873. doi: 10.1371/journal.pone.0321873 (PMC12151346; doi:10.1371/journal.pone.0321873)
Supplement: Attachment — Submitted filename: Response_to_Reviewers_auresp_2.docx [file pone.0321873.s009.docx]

**Response to Academic Editor and Reviewers**

**Authors: We appreciate for spending your precious time and forwarding your valuable comments, which have significantly improved our manuscript. We are also grateful for this positive feedback. Please see below, bold, for a point-by-point response to Academic Editor and the reviewers. We've copied your comments and responses below to make things easier for you.**

**Academic Editor**

1. The manuscript would benefit from language editing by a fluent English speaker to improve overall readability.

**Authors: Thank you for your positive feedback. We have revised the entire manuscript with the assistance of a local editor and a professor to ensure appropriate language editing**

2. When reporting prevalence percentages, please also indicate the numerator and denominator (e.g., x/y, z%) to enhance clarity.

**Authors: Thank you for bringing this issue to our intention. We have provided the numerator and denominator of the prevalence result along with percentages to enhance clarity. Please do not be confused by the fact that the pooled prevalence is not identical to the crude proportion calculated. For example, the pooled prevalence of 20.30% is not identical to the crude proportion calculated as 931/5305. In meta-analysis, the pooled prevalence (20.30%) generated by STATA is not a simple average or direct proportion. Rather, it is weighted summary estimates derived from a meta-analysis model specifically, a random-effects model which accounts for variation across studies, differential weighting, and heterogeneity. Therefore, we kindly request that the prevalence results to be interpreted within this context.**

3. In Supplementary Table 2, only one author is listed as performing data extraction, which does not align with the description in lines 212–213 of the main text. Please correct this inconsistency.

**Authors: Thank you for bringing this to our attention. We have reviewed and corrected the inconsistency in the number of authors between Supplementary Table 2 and the main text.**

**Reviewer #1:** All my previous comments have been addressed.
However I am slightly confused by the different prevalences reported - perhaps because of numerators and denominators "The predominant ESBL producers were K. pneumoniae, with a pooled prevalence of 40.84% (95% CI: 26.87–54.81%), followed closely by E. coli at 40.14% (95% CI: 27.83–52.45%). In the subgroup analysis, the highest magnitude of ESBL producing pathogens was observed in Asia (28.5534.97%), followed by Africa (19.1220.75%). Additionally, the highest pooled prevalence of ESBL-producing pathogens among HIV-positive individuals was reported to be colonization 23.78% (95% CI: 15.36–32.19, I² = 96.78%, p <0.001), followed by infection 15.77% (95% CI: 10.06–21.49, I² = 97.45%, p < 0.001). "

**Authors: We appreciate your feedback. We have checked and updated prevalence results via provided the numerator and denominator of the prevalence result along with percentages to enhance clarity. However, Please do not be confused by the fact that the pooled prevalence is not identical to the crude proportion calculated. For example, the pooled prevalence of 20.30% is not identical to the crude proportion calculated as 931/5305. In meta-analysis, the pooled prevalence (20.30%) generated by STATA is not a simple average or direct proportion. Rather, it is weighted summary estimates derived from a meta-analysis model specifically, a random-effects model which accounts for variation across studies, differential weighting, and heterogeneity. Therefore, we kindly request that the prevalence results to be interpreted within this context.**

**Reviewer #1:** The pooled prevalence of ESBL E coli is reported above at 40% and below at 15-23%. Also for the subgroup analyses by country there are prevalences around 20% by continent while overall it is 40%.

**Authors: Thank you for reflecting on your concern. The overall pooled prevalence of ESBL-producing E. coli (e.g., 40%) represents a weighted average from all included studies, regardless of location, time, or population. Subgroup analyses by continent show averages within smaller groups, often with different numbers of studies, sample sizes, and local epidemiology. It's entirely possible for subgroups to show lower prevalence rates like 15–23% while the overall prevalence is higher, especially when High-prevalence studies (e.g., from specific countries) had large sample sizes or more weight in the analysis. Furthermore, there are fewer studies from low-prevalence areas, giving them less impact on the pooled estimate.**
